# The reliability and heritability of cortical folds and their genetic correlations across hemispheres

**DOI:** 10.1038/s42003-020-01163-1

**Published:** 2020-09-15

**Authors:** Fabrizio Pizzagalli, Guillaume Auzias, Qifan Yang, Samuel R. Mathias, Joshua Faskowitz, Joshua D. Boyd, Armand Amini, Denis Rivière, Katie L. McMahon, Greig I. de Zubicaray, Nicholas G. Martin, Jean-François Mangin, David C. Glahn, John Blangero, Margaret J. Wright, Paul M. Thompson, Peter Kochunov, Neda Jahanshad

**Affiliations:** 1 Imaging Genetics Center, Mark and Mary Stevens Neuroimaging and Informatics Institute, Keck School of Medicine of USC, Marina del Rey, CA USA; 2 Institut de Neurosciences de la Timone, UMR7289, Aix-Marseille Université & CNRS, Marseille, France; 3 Department of Psychiatry, Boston Children’s Hospital and Harvard Medical School, Boston, MA USA; 4 Yale University School of Medicine, New Haven, CT USA; 5 Université Paris-Saclay, CEA, CNRS, Neurospin, Baobab, Gif-sur-Yvette, France; 6 CATI, Multicenter Neuroimaging Platform, Paris, France; 7 School of Clinical Sciences and Institute of Health and Biomedical Innovation, Queensland University of Technology, Brisbane, QLD 4000 Australia; 8 Faculty of Health, Queensland University of Technology (QUT), Brisbane, QLD 4000 Australia; 9 QIMR Berghofer Medical Research Institute, Brisbane, QLD Australia; 10 South Texas Diabetes and Obesity Institute, University of Texas Rio Grande Valley School of Medicine, Brownsville, TX USA; 11 Queensland Brain Institute, University of Queensland, Brisbane, QLD 4072 Australia; 12 Centre for Advanced Imaging, University of Queensland, Brisbane, QLD 4072 Australia; 13 Maryland Psychiatric Research Center, Department of Psychiatry, University of Maryland School of Medicine, Baltimore, MD USA

**Keywords:** Heritable quantitative trait, Neuroscience

## Abstract

Cortical folds help drive the parcellation of the human cortex into functionally specific regions. Variations in the length, depth, width, and surface area of these sulcal landmarks have been associated with disease, and may be genetically mediated. Before estimating the heritability of sulcal variation, the extent to which these metrics can be reliably extracted from in-vivo MRI must be established. Using four independent test-retest datasets, we found high reliability across the brain (intraclass correlation interquartile range: 0.65–0.85). Heritability estimates were derived for three family-based cohorts using variance components analysis and pooled (total N > 3000); the overall sulcal heritability pattern was correlated to that derived for a large population cohort (N > 9000) calculated using genomic complex trait analysis. Overall, sulcal width was the most heritable metric, and earlier forming sulci showed higher heritability. The inter-hemispheric genetic correlations were high, yet select sulci showed incomplete pleiotropy, suggesting hemisphere-specific genetic influences.

## Abstract (web summary)

Fabrizio Pizzagalli et al. study the genetics factors that regulate cortical folding patterning in the brain. They use in vivo brain MRI datasets from around the world and report the reliability and heritability of sulcal morphometric characteristics for 61 sulci per hemisphere of the human brain. They uncover hemisphere-specific genetic influences on the cerebral cortex.

## Introduction

Genetic drivers of brain structural and functional differences are important to identify as potential risk factors for heritable brain diseases, and targets for their treatment. Large-scale neuroimaging consortia, including the ENIGMA^1^ consortium, have identified common genetic variants that have small but significant associations with variations in brain morphology^2^. Studies have even identified genetic correlations between human brain structure and risk for disease^3,4^.

Enriched in neuronal cell bodies, the cortical gray matter plays an important role in human cognitive functions and behavior, including sensory perception and motor control^5^. Macroscale anatomical features of the human cortex can be reliably extracted from structural magnetic resonance imaging (MRI) scans, and among the most common are regional thickness and surface area measures. These MRI-based features show robust alterations in several neurological, neurodevelopmental, and psychiatric disorders^6^, and are influenced by both environmental and genetic variation^7^.

Gyrification of the cortical surface occurs in an orchestrated pattern^8^ during fetal life and into adolescence^9^, forming sulci (fissures) and gyri (ridges) in the cortical gray matter. The mechanisms of brain folding are not fully understood^10,11^, but the process is largely preserved among humans and nonhuman primates. The brain sulci delimit cortical areas with specific functionalities and are generally consistent across subjects^12–15^. The complexity and intersubject variability of brain gyrification are influenced by developmental, aging, and pathological processes, all of which are genetically and environmentally influenced^16,17^.

Large-scale neuroimaging studies have begun to discover common and rare genetic variants that contribute to brain variability as estimated using in vivo brain scans, such as MRI^18^; genome-wide association studies (GWAS) find that, as with other complex traits, individual common variants typically explain <1% of the population variance in MRI derived measures; still, common genetic factors account for a large fraction of the variance in aggregate^2,19–21^. Successful efforts to discover common variants that affect cortical structure require tens of thousands of scans, as well as independent samples for replication and generalization. Large-scale biobanks have amassed tens of thousands of MRI scans^22^. Even so, to replicate effects and ensure the generalizability of findings to other scanned populations, we must first assess that the brain measures are reliably extracted across a variety of possible MRI scanning paradigms. This reliability is the basis for pooling statistical effects across individual studies in multisite consortia such as ENIGMA^1^ and CHARGE^23^.

Sulcal-based morphometry provides in-depth analyses of the cortical fissures, or folds, as seen on MRI. Measures of sulcal morphometry—including length, depth, width, and surface area—among others—have been associated with brain maturation in adolescents^24^, neurodegenerative changes in the elderly^24,25^, and neuropsychiatric disorders such as schizophrenia^26,27^, bipolar disorder^28^, and autism spectrum disorder^29^; altered fissuration is also found in several genetic disorders, such as Williams syndrome^30,31^. Effects on sulcal patterns have been reported as being partially independent of those on cortical thickness or surface area^24,32^.

Effects on sulcal patterns have been reported being partially independent of those on cortical thickness or surface area^24,32^. Previous studies have investigated the genetics of cortical folds, but not across the full brain, and without ensuring the reliability of the measures themselves, or the heritability estimates. Kochunov et al.^33^ analyzed the effects of age on sulcal shape descriptors in a subset of 14 sulci finding wider sulci with older age  in the adult human brain. The central sulcus has been the focus of many earlier publications^34–37^. Its depth has been reported to be highly heritable, with the degree of heritability varying along its profile^37^. In recent works, La Guen et al.^38^ studied the heritability of sulcal pits in the Human Connectome Project (HCP) and the genetic correlation of sulcal width across ten sulci in the UK Biobank^39^. The heritability of the depth, length, and surface area of primary sulci has been studied in baboons^40^. It has been suggested that deeper, earlier forming, sulci have higher heritability^41^, although this hypothesis has not been confirmed. The reliability of the findings across populations, and the extent to which heritability depends on the reliability of the measures, has not been investigated.

Here we: (a) estimate the reliability of four shape descriptors extracted from sulci across the whole brain; (b) evaluate heritability of these measures across four independent cohorts (three family-based cohorts and one cohort of unrelated participants); (c) determine the extent to which the heritability estimates depend on reliability; and (d) provide insights into the relationship between early forming sulci and higher heritability as well as cortical lateralization.

We performed an extensive reliability (*N* = 110) and heritability (*N* = 13,113) analysis. Reliability was estimated from four cohorts, totaling 110 participants (19–61 years of age, 47% females) who underwent two T1-weighted brain MRI scans across different brain imaging sessions. We included data sets for which we would expect minimal or no structural changes between scans, so we limited the analysis to healthy individuals aged 18–65, with an inter-scan interval < 90 days. See Table 1 for more details.

**Table 1:** Cohorts analyzed for the test–retest study.

Cohorts	Age range (mean)	No. of subjects (%F)	Inter-scan interval (days)	Field strength [*T*]	Voxel size [mm]^3^
KKI	22–61 (31.8)	21 (48%)	14	3	[1 × 1 × 1.2]
HCP	24–35 (30.1)	35 (44%)	90	3	[0.7 × 0.7 × 0.7]
OASIS	19–34 (23.3)	20 (60%)	90	1.5	[1.0 × 1.0 × 1.25]
QTIM	21–28 (23.2)	34 (37%)	90	4	[0.94 × 0.90 × 0.94]

HCP and QTIM were used for the reproducibility analysis as they were representative of subjects examined in the genetic analysis. Among publicly available data sets we selected KKI and OASIS, as in ref. ^52^, based on age (18 < age < 65) and inter-scan interval (<90 days).

We analyzed heritability in four independent cohorts, three with a family-based design and one using single-nucleotide polymorphism (SNP)-based heritability estimates. The cohorts included two twin-based samples (Queensland Twin Imaging study (QTIM) and HCP), one cohort of extended pedigrees (the Genetics of Brain Structure and Function; GOBS), and another of over 9000 largely unrelated individuals (the UK Biobank) (Table 2). Heritability estimates are population specific, but here our aim was to understand the heritability pattern across populations and estimate the degree to which genetic effects are consistently observed. We pooled information from all twim and family-based cohorts to estimate the generalized heritability values using meta- and mega-analytic methods^42,43^.

**Table 2:** Genetic analysis: demographics for the four cohorts analyzed in this study.

Cohort	*N* (%F)	Race/Ethnicity/Ancestry	Age in years (mean ± stdev [range])	Relatedness
QTIM	1008 (37%)	European ancestry	22.7 ± 2.7 [18–30]	376 DZ 528 MZ 104 siblings
HCP	816 (44%)	US population with multiple racial and ethnic groups represented	29.1 ± 3.5 [22–36]	205 DZ 199 MZ and triples 412 siblings
GOBS	1205 (64%)	Mexican-American ancestry	47.1 ± 14.2 [18–97]	71 families/pedigrees
UK Biobank	10,083 (47%)	British White	62.4 ± 7.3 [45–79]	Unrelated

We estimated reliability and heritability for measures of each sulcus in the left and right hemispheres, separately. As there is limited evidence for genetic lateralization across most of the human brain^44–46^, we also evaluated the heritability estimates of the measures for each sulcus averaged across the two hemispheres. This may lead to more stable measurements and, if the bilateral measures are influenced by similar genetic factors, then more stable measures could lead to better powered genetic studies. We also assessed the genetic correlation between the measures across hemispheres. Sulci with limited genetic correlations between hemispheres may reveal novel insight into the brain’s lateralization and identify key biomarkers for relating lateralized traits, such as language and handedness, to brain structure^47^.

## Results

### Measurement reliability and its relationship to heritability

Supplementary Data 1 reports the sulcal nomenclature, including the abbreviation and full name for each sulcus. Reliability estimates may be found in Supplementary Data 2–4 for intraclass correlation (ICC) and Supplementary Data 5–8 for the bias evaluation; heritability estimates are reported in Supplementary Data 9–20 for the univariate analysis and Supplementary Data 21–24 for the bivariate analysis. We summarize the results below.

### Intraclass correlation (ICC)

**Fig. 1:**
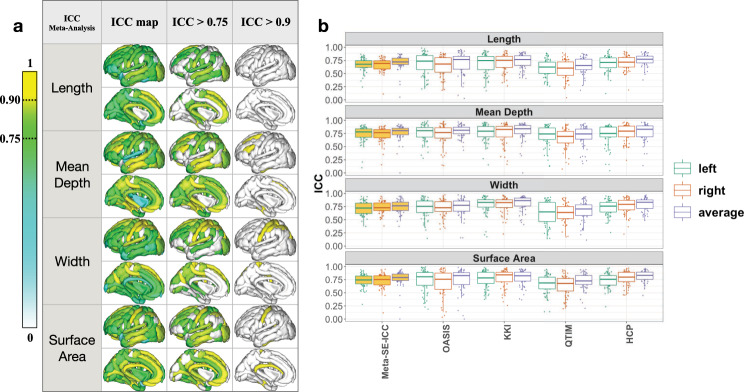
Intraclass correlation reliability estimates for sulcal length, depth, width and surface area. **a** Sulcal-based meta-analysis of intraclass correlation (ICC) for bilaterally averaged sulcal measures (*N* = 110). Sulcal length showed generally “good” reproducibility, although no regions had ICC > 0.9^59^. Mean depth showed “excellent” reproducibility (ICC > 0.9) for: the inferior frontal sulcus (S.F.inf.) and the superior frontal sulcus (S.F.sup.); sulcal width showed “excellent” reproducibility for: intraparietal sulcus (F.I.P.), superior postcentral intraparietal superior sulcus (F.I.P.Po.C.inf.), central sulcus (S.C.), superior postcentral sulcus (S.Po.C.sup.). Surface area showed “excellent” reproducibility for the central sulcus (S.C.), subcallosal sulcus (S.Call.), and the anterior occipito-temporal lateral sulcus (S.O.T.lat.ant.). **b** The intraclass correlation (ICC) for left, right, and bilaterally averaged sulcal length, mean depth, width, and surface area across the whole brain is plotted for the four test–retest cohorts. KKI showed the highest ICC across sulci.

The ICC meta-analysis resulted in an ICC interquartile range of 0.59–0.82 Sulcal mean depth, width, and surface area showed similar reliability estimates, while the length showed lowest ICC (Table 3 and Fig. 1). For all descriptors other than mean depth, a higher fraction of sulci had “good” reliability, defined as ICC > 0.75^48^, after averaging metrics across corresponding left and right hemispheres, for all the descriptors (Fig. 1b) The fraction of sulci reaching ICC > 0.75 went from 24 (before averaging) to 39% (after averaging) for sulcal length, from 37 to 48% for the width, and from 48 to 59% for the surface area; mean depth remained consistant at 57%.

**Table 3:** Meta-analysis of ICC estimated from four independent cohorts for sulcal length, mean depth, width, and surface area.

Meta-analysis	Length	Mean depth	Width	Surface area
Left	0.67 ± 0.12 [0.62–0.74]	0.74 ± 0.15 [0.68–0.84]	0.71 ± 0.12 [0.62–0.81]	0.73 ± 0.12 [0.67–0.82]
Right	0.66 ± 0.12 [0.59–0.74]	0.73 ± 0.14 [0.66–0.82]	0.73 ± 0.11 [0.64–0.81]	0.73 ± 0.13 [0.67–0.81]
Average	0.71 ± 0.14 [0.59–0.74]	0.78 ± 0.11 [0.66–0.82]	0.76 ± 0.12 [0.67–0.82]	0.78 ± 0.11 [0.65–0.83]

Left and right hemisphere and bilaterally averaged mean ± standard deviation (SD) are reported with ICC interquartile range [25–75%] across sulci.

The meta-analysis of ICC captures the consensus  in the reliability across cohorts for each sulcus. Reliability measures depend to some extent on the cohort examined, or the scanning acquisition parameters. For example, for QTIM, which was collected at 4 T, the ICC is classified as “good” (ICC > 0.75) for the left sulcal surface area of the *collateral sulcus* (F.Coll.), but “poor or moderate” (ICC < 0.75) in OASIS for the same trait. Figure 1a shows the meta-analysis of ICC across the four cohorts, and highlights patterns for “good” and “excellent” (ICC > 0.9) reliability.

For a detailed breakdown of the ICC for measures of sulci morphometry per cohort, please see Supplementary Fig. 1 for the left hemisphere, Supplementary Fig. 2 for the right, and Supplementary Fig. 3 for bilaterally averaged measures.

For the complete meta-analyzed ICC results, please see Supplementary Figs. 4–7 for length, depth, width, and surface area respectively, all of which are tabulated in Supplementary Data 4.

For each sulcus, we averaged the reliability estimates across all four sulcal descriptors to find the most reliable sulci overall. The *central sulcus* (S.C.) gave the most reliable sulcal measures, followed by the *median frontal sulcus* (S.F.median), the *intraparietal sulcus* (F.I.P.), the *occipito-temporal lateral sulcus* (S.O.T.lat.ant.), the Sylvian sulcus (S.C.Sylvian), the *sub-parietal sulcus* (S.s.P.), the *occipital lobe*, and the *superior temporal sulcus* (S.T.s.) (Supplementary Fig. 8).

### Bias (*b*)

We explored test–retest (TRT) consistency in terms of the “bias” (*b*, Eq. (4)), with Bland–Altman analyses. As in ref. ^49^, the generally low bias values showed high TRT consistency of sulcal shape measures (Supplementary Data 5). Bias values ≥ 0.1 are considered high, and were noted mainly for length estimates—e.g., for the length of the left and right *anterior/posterior sub-central ramus of the lateral fissure* (F.C.L.r.sc.ant./post.), and the length of the left and right insula (See Supplementary Data 6–8 for bias estimates across the left, right and bilaterally averaged sulcal metrics). Paralleling the higher ICC in bilaterally averaged measures, lower “bias” estimates were obtained with individual sulcal measures averaged across the left and right hemispheres (Supplementary Data 8).

ICC and bias (*b*) of bilaterally averaged sulcal metrics were significantly negatively correlated for all metrics except for length, in particular *r*_length_ = −0.11 [*pval* = 0.07], *r*_mean-depth_ = −0.14 [*pval* = 0.02], *r*_width_ = −0.25 [*pval* = 4.6 × 10^−5^], *r*_surface-area_ = −0.25 [*pval* = 1.2 × 10^−5^], suggesting, as expected, that a lower bias between test and retest measurements relates to higher reproducibility as estimated by ICC.

### Heritability estimates for the cortical folding patterns

Across descriptors and sulci, heritability estimates (*h*^2^) showed a similar pattern across the three family-based cohorts, QTIM, HCP, GOBS (Supplementary Figs. 9–11); the GOBS cohort shows lower heritability, (*h*^2^ = 0.3 ± 0.1), compared to QTIM (*h*^2^ = 0.4 ± 0.1) and HCP (*h*^2^ = 0.4 ± 0.1); GOBS is a cohort with an extended pedigree design and a wide age range (18–85 years of age), while both HCP and QTIM are twin-based cohorts of young adults aged 25–35 years and 20–30 years, respectively.

**Fig. 2:**
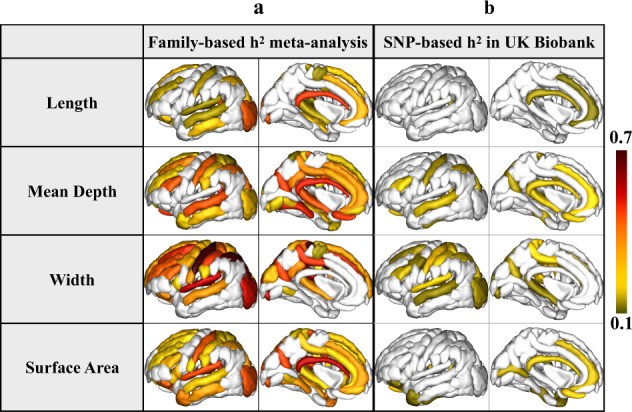
Heritability estimates. Heritability estimates (*h*^2^) are mapped, for each bilaterally averaged sulcal descriptor. **a** The results of the inverse-variance weighted meta-analysis of the heritability estimates across three family-based cohorts QTIM, HCP, and GOBS highlight an overall heritability profile across 3030 individuals. **b** Heritability estimates (*h*^2^) calculated from sulcal features extracted from MRI scans of 10,083 unrelated individuals scanned as part of the UK Biobank were calculated using the genome-wide complex trait analysis (GCTA) package. The regional sulcal metrics that were found to be significantly heritable in the large population sample largely overlap with those found to be most highly heritable across the family-based studies. We highlight only regions that had significant heritability estimates in sulci that had an ICC > 0.75 (see Supplementary Data 2–4 for sulcal-based values of ICC). Significant regions survived Bonferroni correction for multiple comparisons across all bilateral traits and regions (*p* < 0.05/(61 × 4)); darker red colors indicate higher heritability estimates. The left hemisphere was used for visualization purposes.

The generalized heritability profile of cortical folding was obtained by meta-analyzing the estimates across these three independent family-design cohorts, and is highlighted in Fig. 2a. Aggregate heritability estimates were also calculated in a mega-analytic manner, where 3030 subjects from the family-based cohorts (QTIM, HCP, and GOBS) were pooled (after adjusting for covariates within cohort and normalizing across cohorts) before computing heritability estimates as in prior work^42,43^. As expected, we found similarities between meta- and mega-analysis derived heritability estimates as indicated by a significant Pearson’s correlation between these two approaches (*r* ∼ 0.84, *p* = 10^−3^–10^−7^; see Supplementary Fig. 13 for more details). Individual heritability estimates, standard errors (SE), and *p* values for bilaterally averaged sulcal length, mean depth, width, and surface area are tabulated in Supplementary Data 9–18 for each cohort, and in Supplementary Data 19–20 for the meta- and mega-analyses.

For many sulcal features in the UK Biobank, the SNP-based heritability estimates were ~25% of the estimates derived from the family-based studies (*h*^2^ = 0.2 ± 0.1; Fig. 2b). The heritability estimates for the UK Biobank are reported in Supplementary Data 18.

Across the cortex, the global sulcal descriptors were significantly heritable for all cohorts. The patterns of heritability estimates were largely coherent between the family-based and large-scale population studies. The width was the most heritable measurement, while the length was the least, showing significant heritability estimates for only sparse regions of the cortex. The heritability of sulcal length was more frequently significant when not adjusting for ICV; we find minimal differences in the overall *h*^2^ estimates for sulcal depth and width before and after covarying for ICV (Supplementary Fig. 14).

The overall meta-analyzed reliability was significantly correlated with the heritability estimates meta-analyzed across the family-based cohorts: *r* = 0.36 (*pval* = 1 × 10^−7^) for sulcal length, *r* = 0.31 (*pval* = 4.1 × 10^−6^) for mean depth, *r* = 0.26 (*pval* = 7 × 10^−5^) for sulcal width, and *r* = 0.25 (*pval* = 1 × 10^−4^) for surface area (Supplementary Fig. 15); the reliability estimates were also correlated with heritability estimates in the UK Biobank for mean depth (*r* = 0.43, *pval* = 2 × 10^−3^) and sulcal width (*r* = 0.38, *pval* = 4 × 10^−3^) (Supplementary Fig. 16).

A few bilaterally averaged sulcal regions and metrics with “poor” reliability (ICC < 0.75) showed significant heritability estimates. These included the length of the *parieto-occipital fissur*e (F.P.O.) [ICC = 0.66, *h*^2^ = 0.18 (*pval* = 1 × 10^−5^)], the mean depth of the *ascending ramus of the lateral fissure* (F.C.L.r.asc.) [ICC = 0.74, *h*^2^ = 0.2 (*pval* = 2.2 × 10^−6^)], the surface area of the *anterior inferior frontal sulcus* (S.F.inf.ant.) [ICC = 0.65, *h*^2^ = 0.17 (*pval* = 4.7 × 10^−6^)], and the width of the *calloso-marginal ramus of the lateral fissure* (F.C.M.ant.) [ICC = 0.63, *h*^2^ = 0.34 (*pval* = 1 × 10^−16^)] (Supplementary Data 4 and 19). For UK Biobank, the length of S.T.pol. [ICC = 0.70, *h*^2^ = 0.14 (*pval* = 6 × 10^−5^)], the width and the surface area for the *insula* [ICC = 0.65, *h*^2^ = 0.14 (*pval* = 2.6 × 10^−5^)] and [ICC = 0.65, *h*^2^ = 0.16 (*pval* = 3.8 × 10^−6^)], respectively (Supplementary Data 18).

The heritability estimates for the global measures (i.e., the sum across sulci) of sulcal length, mean depth, width, and surface area (covarying for ICV, age, and sex variables) are also reported in Supplementary Fig. 17. QTIM, HCP, and GOBS showed similar trends across descriptors and hemispheres; only QTIM had generally higher heritability estimates for sulci in the right hemisphere compared to those in the left (paired *t*-test: *pval* = 1.5 × 10^−10^).

Thirty-three percent (36% for mega-analysis) of the total number of bilaterally averaged sulci showed significant *h*^2^ for sulcal length, 57% (59% for mega-analysis) for mean depth, 67% (65% for mega-analysis) for width, and 62% (60% for mega-analysis) for the surface area. Six sulci were significantly heritable for only one of the four descriptors (one for mega-analysis). No sulcus show significant heritability for length only. Sulci that were significantly heritable across descriptors included the *intraparietal sulcus*, *occipital lobe*, *subcallosal sulcus*, *internal frontal sulcus*, *orbital sulcus*, *anterior inferior temporal sulcu*s, and the *polar temporal sulcus*, among others; in total 15 sulci were significantly heritable across all four descriptors in the meta-analysis, and 19 for the mega-analysis (see Supplementary Data 19–20).

A significant Pearson’s correlation was identified between heritability estimates averaged across sulcal descriptors and the approximate appearance of sulci (in weeks) during development^50^ (Supplementary Fig. 17, *r* = −0.62, *p* = 0.0025).

### Genetic correlations between sulcal shape descriptors of the left and right cortical hemispheres

*Genetic correlation across the hemispheres*: Averaging brain-imaging derived traits across the left and right hemispheres, as above, has been shown to reduce noise due to measurement error in large scale, multi-cohort efforts^2,20,51,52^. Improvements in the signal-to-noise ratio may be essential for discovering single common genetic variants that explain < 1% of the overall population variability in a trait. However, by assessing left and right separately, we may be able to discover lateralized genetic effects, if they exist.

Bivariate variance components models confirmed that the genetic correlations between the same global sulcal descriptor on the left and right hemispheres of the brain were significant (*ρ*_*G*_ ∼ 0.92 ± 0.10) (Supplementary Data 21–24).

**Fig. 3:**
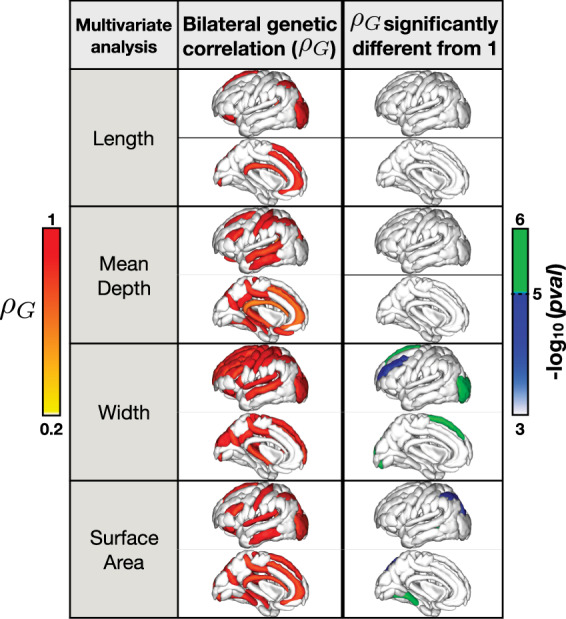
Genetic correlations between sulcal shape descriptors of the left and right cortical hemispheres. Left: the genetic correlations (*ρG*) between corresponding sulcal descriptors on the left and right hemispheres were assessed in three family based cohorts and meta-analyzed correlation values are mapped onto the brain. Right: the −log10 of the *p* value comparing the resulting genetic correlation to a perfect overlap (*ρG* = 1) are mapped. Significant values here suggest that the genetic components of variance may be partially unique across the left and right homologous sulcal metrics; i.e, despite a genetic correlation between hemispheres, lateralized genetic effects may be detectable. Sulci are mapped to the left hemisphere for visualization purposes.

The genetic correlation (*ρG*) between left and right homologous regions was computed for sulcal metrics that showed both “good” reliability estimates (ICC > 0.75) and significant univariate heritability estimates for both the left and right metrics; (Fig. 3, Supplementary Data 21–24). The genetic correlations between the left and right sulcal metrics was generally highest for the sulcal width metric. The width of the central sulcus, the inferior frontal sulcus, intermediate frontal sulcus, superior frontal sulcus, posterior lateral sulcus, the superior postcentral intraparietal superior sulcus, and the intraparietal sulcus, and the surface of the occipital lobe showed significant genetic correlations across all tested cohorts.

Two sets of *p* values are obtained when performing genetic correlations with bivariate variance components models: a more traditional *p* value comparing the correlation to the null hypothesis of no correlation (*ρG* = 0), and another *p* value comparing the genetic correlation to the hypothesis of a perfect overlap (*ρG* = 1), assessing the difference between the genetic correlation obtained and a correlation of 1. Bonferroni correction for the more traditional *p* values was conducted by correcting for the number of traits tested  so *p* < 0.05/[*N*], where *N* = 11 heritable sulci with ICC > 0.75 for length, +31 for mean depth +36 for width +37 for surface area, for a total of 115 traits. The second set of *p* values comparing the genetic correlation (*ρ*_*G*_) between hemisphere homologs to the indistinguishable value of 1 are listed in Supplementary Data 24, and the −log10(*p* values) of those significantly different than 1 are mapped in Fig. 3. For these regions, the 95% confidence interval surrounding the correlation estimate did not contain 1. These sulci represent regions and descriptors that may have diverging genetic influences across hemispheres. The meta-analysis revealed evidence for lateralized genetic effects in sulcal width or surface area of the occipital lobe, the intraparietal lobe, the median frontal sulcus, the intermediate left frontal sulcus, and the collateral sulcus; no evidence for lateralization of genetic influences was detected with either sulcal length or depth metrics.

Phenotypic correlations (*ρ*_*P*_) between the left and right indices were on average less than 0.5 in each cohort (Supplementary Fig. 18). Sulcal width showed the highest (*ρ*_*P*_ = 0.38 ± 0.15) meta-analyzed correlation between left and right homologs compared to the other sulcal descriptors (0.29 ± 0.07 for sulcal length, 0.30 ± 0.11 for mean depth, and 0.33 ± 0.12 for surface area).

## Discussion

Our study has four main findings: (1) many of the sulci common across individuals were reliably extracted across a variety of MRI acquisition parameters, with some sulcal shape descriptors being more reliable than others; (2) cortical folding patterns were highly heritable and sulcal shape descriptors such as sulcal width may be promising phenotypes for genetic analysis of cortical gyrification; (3) the proportion of variance attributed to additive genetic factors  varied regionally, with the earlier forming sulci having higher heritability estimates than later forming sulci; (4) incomplete pleotropy was identified between select left and right sulcal descriptors, suggesting sulcal analyses may provide insights into genetic factors underlying the lateralization of brain structure.

Cortical sulci may serve as prominent landmarks for identifying homologous functional regions across individuals^35,36^. BrainVISA offers the ability to automatically extract and characterize the sulci at high spatial resolutions, by segmenting and labeling 123 sulci across the cerebral cortex. Here, we analyzed four sulcal shape descriptors: length, mean depth, width, and surface area. Sulcal length has been associated with neurodevelopmental processes^29,53,54^, while sulcal depth and width have been correlated with aging and neurodegenerative processes^33,55–57^. Sulcal surface area represents a combination of depth, width and length features.

A primary goal of this work was to identify the sulci and the corresponding shape metrics that may be reliably extracted irrespective of the specific MRI scanner or  scan acquisition protocol, to ensure a globally viable trait for disease related biomarker and genetic association analyses. Poor reliability may be attributable to measurement errors, which could lead to a ceiling effect on heritability estimates. This is because highly heritable traits can only be detected if the traits are robustly measured^58^ and low reliability could lead to an underestimation of the true heritability^59^. While heritability is a population-specific estimate, one main goal of *imaging genetics* consortia such as ENIGMA^1^ and CHARGE^23^ is to identify genetic variants that affect brain structure and function in populations around the world. Therefore, it is of utmost importance to ensure that measures are reliably extracted across different data sets, and furthermore, are heritable  across different populations. Even beyond imaging genetics, the reliability of the measurements and the reproducibility of any set of results are essential for reproducible science at large.

Here we identified the most reliable sulcal regions using test-retest (TRT) data from four cohorts with independent samples and different scanning protocols to ensure the robustness of results. We assessed bias, a subject-based index of consistency^49^ as well as ICC, which compares the within-subject variance to the between-subjects variance. ICC may be affected by the homogeneity of the population under study; when variability in the population is low, for example, if age range is limited, then lower ICC values may be expected, while bias would be unaffected. Our results show high consistency between test and retest (“bias” < 0.1^49^ on average). Furthermore, in considering the number of sulci that had ICC estimates  greater than 0.9, sulcal width was the most reliable metric among the descriptors analyzed. Although some visual quality control was conducted on individual sulcal extractions, we did not ensure the anatomical validity of the entire set of sulcal labels for each of the individual MRI scans used in this study.  Our reliability results are therefore more a reflection of methodological consistency, rather than anatomical accuracy.

A study examining the relationship between reproducibility and heritability of different brain structures in the QTIM cohort^60^ found a correlation between ICC and heritability, with a large percentage of traits showing low reliability (ICC < 0.75)^60^. Here we showed that most of the reliable sulcal shape descriptors were also highly heritable. This trend might be due to the lower variance across subjects for more robust anatomical regions, such as the central sulcus, which are easier to identify with automated image processing pipelines and less prone to segmentation errors. However, even in regions with “excellent” reliability (ICC > 0.9), we identified a range of heritability estimates, suggesting  that not all reliable traits are necessarily highly heritable^59^.

Many earlier works have focused exclusively on the central sulcus^37,40,61^. We have replicated findings of significant heritability in the central sulcus and further, showed that it is indeed the sulcus  with the highest heritability estimate across the entire cortex. However, out of 61 total bilateral cortical sulci, it is only one of 34 that showed significant heritability estimates across all four shape descriptors.

Our results indicated significant heritability estimates for sulcal surface area and width in several medial frontal regions, partially confirming findings in ref. ^40^. Our results also confirmed prior findings of sulcal heritability in the temporal lobe^62^ and the corpus callosum area^63^ and are also in line with studies showing high estimated heritability in prefrontal and temporal lobes for cortical thickness and surface area^64–70^, especially for sulcal mean depth and width.

The sulcal descriptors identified as being heritable in this work may serve as phenotypes for large-scale genome-wide association studies, or GWAS, enhancing  our ability to identify specific genomic variants that influence brain structure and disease risk. These reliable and heritable sulcal measures may also serve as biomarkers for understanding genetically mediated brain disorders. The significant correlation identified between heritability estimates averaged across sulcal descriptors and the appearance of sulci (in weeks) during development^50^ implies that sulci appearing early in brain development^71,72^, including the central sulcus, Sylvian fissure, parieto-occipital lobes, and superior temporal sulcus^50^ may be under stronger genetic control. However, some regions including the frontal lobe and the temporal sulcus also had high heritability, even though these regions are reported to develop later^72^, suggesting more work is needed to identify the developmental role in the regional genetic architecture.

Across three independent family-based cohorts, QTIM—an Australian cohort of young adult twins and siblings—HCP, a North American cohort of twins and siblings, and GOBS—a Mexican-American cohort of extended pedigrees, we found similar patterns of heritability for four descriptors of sulcal morphometry. Globally, we found sulcal heritability estimates of ~0.3–0.4, similar to estimates in other species, including *Papio* baboons^40^. Heritability estimates from GOBS were lower than for QTIM or HCP, as may be expected for an extended pedigree design when compared to twin designs^73^. It has also been proposed that higher image quality, and therefore lower measurement error, could lead to higher heritability estimates^74^. GOBS and HCP MRI volumes were acquired with a 3T scanner and HCP has higher spatial resolution compared to GOBS. QTIM was acquired with slightly lower spatial resolution but at higher magnetic field strength (4T). Further analyses will be needed to investigate how the signal-to-noise estimates (SNR) vary across cohorts and how this affects heritability estimation^74^. SNP-based heritability estimated in the UK Biobank showed a similar *h*^2^ pattern (Supplementary Fig. 20) across the brain, but with lower *h*^2^ values compared to the family-based cohorts. This may be partially due to the “missing heritability” effect in the SNP-based heritability estimation^75^. We note that recent work has found less discrepancy between twin-based heritability estimates and those derived from large-scale population studies of approximately 20,000 individuals^76^, therefore, larger population samples may be required to better power our SNP-based heritability estimates and help determine the true extent of the missing heritability.

Apart from work by the ENIGMA Laterality group^77^, many published ENIGMA studies^2,20,43,78^ performed analyses on pooled bilateral measures of brain structure, averaging data from the left and right hemispheres. We indeed found that for most sulcal descriptors, averaging the sulcal measures across hemispheres provided more regions with reliable estimates, and more consistent heritability estimates across cohorts. The bivariate genetic analysis used to estimate the genetic correlation between left and right sulcal measures, further confirmed strong and significant genetic correlations between hemispheres.

A genetic correlation between measures across the right and left hemispheres indicates pleiotropy, suggesting that genetic influences underlying the structure and variability in the measures tends to overlap. In family-based studies, bivariate variance components analysis may be used to determine the genetic correlation between traits as in this work. When a significant genetic correlation is identified, the confidence interval around the genetic correlation often includes one, suggesting the underlying genetic influences of the measures were not statistically distinguished from each other. Incomplete pleiotropy is suggested when genetic correlations are significant, but the confidence intervals around the correlations do not include one. While in SNP-based genetic correlation models, incomplete pleiotropy may be suggested over complete pleiotropy in the presence of measurement error, in a bivariate polygenic model, measurement error falls into the environmental component of variance and the environmental correlation, and therefore, does not influence the maximum-likelihood estimate of the genetic correlation; i.e, measurement error makes it *more* difficult to reject the null hypothesis that the genetic correlation is one. Features that exhibit unique genetic influences in one hemisphere may reveal insights into the biological causes of brain lateralization that may play an important role in neurodevelopmental or psychiatric disorders. Evidence of less genetic control in the left hemisphere has been found in refs. ^50,62^, where the authors found higher cortical gyrification complexity in the right hemisphere at an early development stage.

Here we found incomplete pleiotropy, or suggested asymmetrical genetic influences, in the frontal lobe (width). This may relate to disorder-specific abnormalities seen in brain folding patterns, for example, as reported in a postmortem study on schizophrenia^79^. Incomplete pleiotropy was also detected in sulci of the occipital lobe, a highly polygenic region^80^; structural abnormalities in this region have been associated with Parkinson’s disease^81,82^, posterior cortical atrophy, a disorder causing visual dysfunction, and logopenic aphasia^83^.

Some regions that showed this suggested lateralization of genetic effects for sulcal descriptors, showed the same effect for other measures extracted from the cortex; for example, the effect seen with the sulcal surface area of the collateral fissure was also detected with the corresponding gyral  surface area (Supplementary Fig. 21). However, for the occipital lobe, we found evidence for lateralization of genetic effects with sulcal width, but not with either cortical thickness or surface area of corresponding gyri. This suggests that sulcal descriptors may offer additional insights into cortical development and lateralization, beyond more commonly analyzed metrics of gyral morphometry. As a larger than expected portion of our study population was right-handed, our findings may be biased towards right handed individuals and may not be fully representive; the degree of cerebral volume asymmetry has been shown to be lower for non-right-handed twins than right-handed pairs^62^ and future investigations focusing on the genetics of brain gyrification and lateralization across handedness are needed to confirm these findings.

The genetic influences on brain cortical structure are regionally dependent, and differ according to the metric, or descriptor, being evaluated. For example, the genetic correlation between average cortical thickness and total surface area has been shown to be weak and negative, with largely different genetic compositions^32^. Different metrics are often used to describe and quantify different biological processes such as those such as length and surface area with potentially more developmental orgins, and others including sulcal width that may capture more degenerative processes. In nonhuman primates, brain cortical folding was also found to be influenced by genetic factors largely independent of those underlying brain volume^84,85^. Measuring cortical folding through sulcal-based morphometry could therefore highlight brain metrics beyond thickness and surface area, and may complement these more traditional measures to reveal a deeper understanding of the processes underlying variation in human brain structure, its association with disease and the underlying genetic risk factors. Our findings suggest that conducting a GWAS of sulcal features may be particularly informative for the sulcal width—the most heritable of the four tested metrics. Although for most sulci, the genetic components of variances were largely indistinguishable (i.e., highly correlated) across the two hemispheres, our results suggest that conducting a separate GWAS of sulcal measures in select frontal, temporal, and occipital regions may provide added insight into the biological mechanisms that drive hemispheric specialization. The discovery and replication of specific genetic influences on brain structure require very highly powered analyses, achievable through large-scale studies and collaboration. Harmonized imaging and genetic analysis protocols, rigorous quality assurance, reproducibility assessments, along with statistical rigor are vital in the collaborative endeavors such as those proposed by the ENIGMA consortium.  To allow for a variety of such international collaborations, the customized MRI image processing protocol using and extending the BrainVISA toolkit as in this work, has been made freely available at: http://enigma.ini.usc.edu/protocols/imaging-protocols/.

## Methods

### Participants and MRI imaging

*Queensland Twin Imaging study (QTIM)*: Brain MRI from 1008 right-handed participants^86^, 370 females and 638 males, were used in this study. This included 376 dizygotic (DZ) and 528 monozygotic (MZ) twins (one set of DZ triplets) and 104 siblings, with an average age of 22.7 ± 2.7 years [range: 18–30]. T1-weighted images were acquired on a 4T Bruker Medspec scanner with an inversion recovery rapid gradient echo sequence. Acquisition parameters were inversion/repetition/echo time (TI/TR/TE) = 700/1500/3.35 ms; flip angle = 8°; with an acquisition matrix of 256 × 256; voxel size = 0.94 × 0.90 × 0.94 mm^3^.

*Human Connectome Project (HCP)*: 816 participants^87^, 362 females and 454 males, average age 29.1 ± 3.5 years [range: 22–36]. These included 412 siblings, 205 DZ and 199 MZ twins, including triplets. T1-weighted images were acquired using a 3T Siemens scanner. MRI parameters: (TI/TR/TE) = 1000/2400/2.14 ms; flip angle = 8°; voxel size = 0.7 mm isotropic; acquisition matrix = 224 × 224. The subset of TRT scans includes all right-handed subjects.

*Genetics of Brain Structure and Function (GOBS)*: A total of 1205 individuals of Mexican-American ancestry from extended pedigrees (71 families, average size 14.9 [1–87] people) were included in the analysis. Sixty-four percent of the participants were female and ranged in age from 18 to 97 (mean ± SD: 47.1 ± 14.2) years. Individuals in this cohort have actively participated in research for over 18 years and were randomly selected from the community with the constraints that they are of Mexican-American ancestry, part of a large family, and live within the San Antonio, Texas region. Imaging data were acquired at the UTHSCSA Research Imaging Center on a Siemens 3T Trio scanner (Siemens, Erlangen, Germany). Isotropic (800 µm) 3D Turbo-flash T1-weighted images were acquired with the following parameters: TE/TR/TI = 3.04/2100/785 ms, flip angle = 13°. Seven images were acquired consecutively using this protocol for each subject and the images were then co-registered and averaged to increase the signal-to-noise ratio and reduce motion artifacts^88^.

*UK Biobank:* Analyses were conducted on the 2017 imputed genotypes restricted to variants present in the Haplotype Reference Consortium^89,90^. UK Biobank bulk imaging data were made available under application #11559 in July 2017. We analyzed 10,083 participants (4807 females), mean age = 62.4 ± 7.3 years [range: 45–79]. Voxel matrix: 1.0 × 1.0 × 1.0 mm—acquisition matrix: 208 × 256 × 256. 3D MP-RAGE, TI/TR = 880/2000 ms, sagittal orientation, in-plane acceleration factor = 2. Raw MRI data were processed using the ENIGMA FreeSurfer and sulcal analysis protocols. Following processing, all images were visually inspected for quality control of FreeSurfer gray/white matter classifications. For all subjects, the central sulcus segmented and labeled by BrainVISA was visually assessed for labeling accuracy.

*KKI (Kennedy Krieger Institute—Multi-Modal MRI Reproducibility Resource):* 21 healthy volunteers with no history of neurological conditions (10 F, 22–61 years old) were recruited. All data were acquired using a 3T MRI scanner (Achieva, Philips Healthcare, Best, The Netherlands) with body coil excitation and an eight-channel phased array SENSitivity Encoding (SENSE) head-coil for reception. All scans were completed during a 2-week interval. The resulting data set consisted of 42 “1-h” sessions of 21 individuals. MP-RAGE T1-weighted scans were acquired with a 3D inversion recovery sequence: (TR/TE/TI = 6.7/3.1/842 ms) with a 1.0 × 1.0 × 1.2 mm^3^ resolution over a field of view of 240 × 204 × 256 mm acquired in the sagittal plane. The SENSE acceleration factor was 2 in the right–left direction. Multi-shot fast gradient echo (TFE factor = 240) was used with a 3-s shot interval and the turbo direction being in the slice direction (right–left). The flip angle was 8°. No fat saturation was employed^91^, https://www.nitrc.org/projects/multimodal/.

*OASIS:* This TRT reliability data set contains 20 right-handed subjects (19–34 years old) without dementia imaged on a subsequent visit within 90 days of their initial session. MP-RAGE T1-weighted scans were acquired on a 1.5-T Vision scanner (Siemens, Erlangen, Germany): (TR/TE/TI = 9.7/4.0/20 ms) with an in-plane resolution of 1.0 × 1.0 × mm^2^ resolution over a FOV of 256 × 256 mm acquired in the sagittal plane. Thickness/gap = 1.25/0 mm; flip angle = 10° (https://www.oasis-brains.org/)^92^.

*MRI image processing and sulcal extraction*. Anatomical images (T1-weighted) were corrected for intensity inhomogeneities and segmented into gray and white matter tissues using FreeSurfer (http://surfer.nmr.mgh.harvard.edu/); segmentations and regional labels were quality controlled using ENIGMA protocols for outlier detection and visual inspection (http://enigma.ini.usc.edu/protocols/imaging-protocols/). BrainVISA (http://brainvisa.info) was run for sulcal extraction, identification, and sulcal-based morphometry. Morphologist 2015, an image processing pipeline included in BrainVISA, was used to quantify sulcal parameters. Briefly, the Morphologist 2015 segmentation pipeline computes left and right hemisphere masks, performs gray and white matter classification, reconstructs a gray/white surface and a spherical triangulation of the external cortical surface, independently for both hemispheres. Sulcal labeling has been performed using BrainVISA suite which implements the algorithm fully described in the cited paper by Perrot et al.^93^. It relies on a probabilistic atlas of sulci. The sulcal recognition is made by combining localization and shape information. The atlas is described in detail and freely accessible here: http://brainvisa.info/web/morphologist.html and can be visualized online here: http://brainvisa.info/web/webgl_demo/webgl.html.

To improve sulcal extraction and build on current protocols used by hundreds of collaborators within ENIGMA, quality controlled FreeSurfer outputs (*orig.mgz, ribbon.mgz, and talairach.auto*) were directly imported into the pipeline to avoid recomputing several steps, including intensity inhomogeneity correction and gray/white matter classification. Sulci were then automatically labeled according to a predefined anatomical nomenclature of 62 sulcal labels for the left hemisphere and 61 sulcal labels for the right hemisphere^94,95^. The protocol developed for this work is available at http://enigma.ini.usc.edu/protocols/imaging-protocols/ (ENIGMA-Sulci).

*Sulci descriptors and quality control.* Analyzing the shape of the cortex through sulcal-based morphometry allows us to quantify the geometry of a sulcus in terms of several distinct and complementary descriptors, consisting of length, mean depth, surface area, and width (or fold opening) of all extracted and labeled sulci. Cortical thickness and surface area have both been found to be moderately to highly heritable, yet with largely independent and even negatively correlated genetic influences^7,80,96^. Cortical thickness, surface area, and folding tend to exhibit different age-related trajectories^97,98^. In particular, cortical thickness represents the laminar organization of the cerebral cortex, which contains about 14 billion neurons^99^. Each of the layers forming the cortex^100^ has a different cellular organization, mostly distinguished on the basis of pyramidal cells in the various laminae^100^. Surface area may reflect the number of radial columns perpendicular to the pial surface^98^ and sulcal morphometry may additionally relate to the microstructure of the neuronal sheets and to the local axonal connectivity within a cortical region, which may influence the degree of folding^84^.

The length of a sulcus is measured in millimeters as the geodesic length of the junction between a sulcus and the hull of the brain. The mean depth corresponds to the average of the depth across all the vertices along the bottom of a sulcus (the depth of a vertex located at the bottom of a sulcus is defined as the geodesic distance along the sulcus to the brain hull). The surface area is the total area of the sulcal surface. The enclosed cerebrospinal fluid (CSF) volume divided by the sulcal surface area gives the width, a gross approximation of the average width of the CSF in the fold^61^ (see Supplementary Fig. 32 for a representation of sulcal shape descriptors).

To further quality control the extracted sulcal measures and identify subjects whose sulci were not optimally identified, we consider as outliers those subjects showing abnormal values for at least one of the descriptors for each sulcus. That is, for a given sulcus, the *z*-score across subjects is computed for each descriptor. The set of subjects showing an absolute *z*-score > 2.5 for one or more descriptors was discarded from further analysis^101^. Therefore, if the length of the central sulcus for a given subject was an outlier but width, depth, and surface area were not, that subject’s central sulcus was removed from further evaluation; this ensured that the same set of subjects were used for all analyses across descriptors. This led to discarding ∼3% of subjects for each sulcus.

### Statistics and reproducibility

*Univariate and bivariate quantitative genetic analyses:* The relative influences of genetic and environmental factors on human traits can be estimated by modeling the known genetic relationship between individuals and relating it to observed covariance in measured traits; in twin studies, MZ twin pairs—who typically share all their common genetic variants—are compared to DZ twin pairs, who share, on average, 50%. The same principle can be used for extended pedigrees, in which many individuals have varying degrees of relatedness. Here, we used both twins and extended pedigrees to estimate the heritability of these in-depth cortical sulcal measures. For a given cohort of participants, the narrow-sense heritability (*h*^2^) is defined as the proportion of the observed variance in a trait (*σ*^2^_*p*_) that can be attributed to additive genetic factors (*σ*^2^_*g*_):

$$h^2 = {{\sigma _g^2} \over {\sigma _p^2}}.$$

Variance components methods, implemented in the Sequential Oligogenic Linkage Analysis Routines (SOLAR) software package^102^, were used for all genetic analyses. Heritability (*h*^2^) is the proportion of total phenotypic variance accounted for by additive genetic factors and is assessed by contrasting the observed phenotypic covariance matrix with the covariance matrix predicted by kinship. High heritability indicates that the covariance of a trait is greater among more closely related (genetically similar) individuals; here, for example, MZ twins as compared to DZ twins and siblings. Using SOLAR-ECLIPSE imaging genetics tools (http://www.nitrc.org/projects/se_linux)^102^, we investigated the heritability profile of four sulcal descriptors for sulci across the whole brain: 62 on the left and 61 on the right hemisphere.

Prior to testing for the significance of heritability, sulcal descriptor values for each individual are adjusted for a series of covariates. We estimated the influence of specific variables (additive genetic variation and covariates including intracranial volume, sex, age, age^2^, age × sex interaction, age^2^ × sex interaction) to calculate the sulcal trait heritability and its significance (*p* value) for accounting for a component of each trait’s variance within this population.

The significance threshold for heritability analysis of individual sulci was set to be *p* ≤ (0.05/m*4), where *m* = 61 (number of bilateral sulci), and the times 4 corresponding to the number of shape descriptors assessed. We set *m* = 123 when left and right sulcal heritability were estimated separately. This reduced the probability of Type 1 errors associated with multiple measurements.

For bivariate genetic correlation estimates, classical quantitative genetic models were used to partition the phenotypic correlation (*ρ*_*P*_) between the left and the corresponding right sulcal measures into the genetic (*ρ*_*G*_), and a unique environmental (*ρ*_*E*_) components, for each pair of traits. Just as with the univariate model, the bivariate phenotype of an individual is modeled as a linear function of kinship coefficients that express relatedness among all individuals within the cohort (MZ twins share all their additive genetic information and DZ twins and siblings share on average 50%). The significance of *ρ*_*G*_ and *ρ*_*E*_ was estimated from the likelihood ratio test when comparing the model to ones where the correlation components are constrained to be 0^102–104^. This estimates *ρ*_*G*_ and *ρ*_*E*_ and their standard error (SE). The significance of these coefficients is determined by a *z*-test of their difference from 0. If *ρ*_*G*_ differs significantly from 0, then a significant proportion of the traits’ covariance is influenced by shared genetic factors.

In this case, we tested another model where the genetic correlation factor *ρ*_*G*_ is fixed to 1. Fixing *ρ*_*G*_ to 1 suggests that the additive genetic components comprising the two traits overlap completely, and there is no detectable unique genetic composition for the individual traits. Once again, the log-likelihood of this model is compared to one where the parameters are freely optimized. If *ρ*_*G*_ is not found to significantly differ from 1, then we cannot reject the hypothesis that both heritable traits are driven by the same set of genetic factors. If *ρ*_*G*_ is significantly different from 0 and significantly different from 1, then the traits share a significant portion of their variance, however, each is also likely to be partially driven by a unique set of genetic factors.

Some considerations should be made regarding the measurement error of the traits analyzed here: *ρ*_*G*_ is the correlation between the latent genetic effects on the two traits irrespective of the proportion of phenotypic variance these latent effects explain (i.e., heritability). Measurement error, which is uncorrelated between individuals regardless of their relatedness, falls into the environmental component and environmental correlations. Measurement error therefore influences *h*^2^, *ρ*_*E*_, *ρ*_*P*_, but not *ρ*_*G*_.

In practice, measurement error does make *ρ*_*G*_ harder to estimate, because low heritability means that the underlying genetic effects cannot be estimated with precision. This causes the SE of the *ρ*_*G*_ estimate to increase, but critically, does not change its maximum-likelihood estimate systematically. So measurement error makes it harder to reject the null hypothesis that *ρ*_*G*_ = 1.

Moreover, the bivariate polygenic model used here to estimate the left–right genetic correlation is a linear function of laterality (*L*–*R*). Indeed, the genetic variance of *L*–*R* is:

$$\sigma _g^2\left( L \right) + \sigma _g^2\left( R \right) - 2 \times \rho _g \times \sqrt {\sigma _g^2\left( L \right) \times \sigma _g^2\left( R \right)},$$

where $\sigma _g^2\left( L \right)$ and $\sigma _g^2\left( R \right)$ are the genetic variance for the left and right traits. The phenotypic variance is similarly defined so that the heritability of *L*–*R* can be obtained. But if *L*–*R* shows significant heritability, it could be because: (1) genetic overlap is incomplete and/or (2) *L* and *R* have unequal genetic variances. So studying laterality is not recommended here because (1) and (2) are confounded.

### Meta-analysis of additive genetic variance

Meta-analysis calculates weighted mean heritability (*h*^2^) and SE estimates based on measurements from individual cohorts^42,43^. We weighted the heritability estimate from each cohort by the heritability SE, as extracted from the variance component model of SOLAR. The heritability weighted by SE^42,43^ is:

1 $$h_{{\rm{MA - SE}}}^2\left( S \right) = \frac{{\mathop {\sum }\nolimits_j se_j^{ - 2} \times h_j^2(S)}}{{\mathop {\sum }\nolimits_j se_j^{ - 2}}},$$

where *S* = 1 to *N*_*s*_ indexes the sulci and *j* = 1,2,3 indexes the cohorts.

### Mega-analysis of additive genetic variance

While meta-analyses compute first the heritability independently for each cohort and then combine the results, mega-analyses combine first different cohorts and then run a single computation for heritability evaluation. We use a program (*polyclass*), developed for SOLAR^105^ for mega-analysis of heritability on sulci descriptors^43,106^. This function fits the model after combining the pedigrees of QTIM, HCP, and GOBS into a single pedigree (for more details see refs. ^42,43^).

### Meta-analysis of genetic correlation

A meta-analysis of genetic correlation is calculated weighting the genetic correlation computed for each cohort by its sample size:

2 $$\rho _{G - {\rm{MA}}}\left( S \right) = \frac{{\mathop {\sum }\nolimits_j \rho _{G_j}^2(S) \times N_{\rm{sub}}}}{{\mathop {\sum }\nolimits_j N_{{\rm{sub}}(j)}}},$$

where *S* = 1 to *N*_*s*_ indexes the sulci, *j* = 1, 2, 3 indexes the cohorts, and $N_{{\rm{sub}}(j)}$ is the sample size of cohort *j*.

To combine *p* values in a meta-analysis, we used the Edgington’s method that represents a compromise between methods more sensitive to largest *p* values (e.g., Pearson’s method) and methods more sensitive to smallest *p* values (e.g., Fisher’s method)^107,108^:

3 $${\rm{Meta}}\,p {\hbox{-}} {\rm{value}} = \frac{{S^k}}{{k!}} - \left( {k - 1} \right)C1\frac{{\left( {S - 1} \right)^k}}{{k!}} + \left( {k - 2} \right)C2\frac{{\left( {S - 2} \right)^k}}{{k!}},$$

where *S* is the sum of *o* values and *k* the number of tests (i.e., *k* = 3 cohorts in our study). The corrective additional terms are used if the number subtracted from *S* in the numerator is *<S*. All the *p* values in the meta-analyses estimated were computed using this method.

### SNP-based heritability analysis

We used genome-wide complex trait analysis (GCTA)^109^ to estimate the heritability from the individual genotypes. Genotypes on the autosomal chromosomes were used to calculate the genetic relationship matrix with GCTA^109^. Heritability was calculated using a linear mixed model, with age, sex, ICV, and the first four genetic components from multidimensional scaling analysis as fixed covariates. We also covaried for the presence of any diagnosed neurological or psychiatric disorder. In our analysis, we excluded participants with non-European ancestry, missing genotypes, or phenotypes, and mismatched sex information.

### Reliability analysis

*Sulcal measurement reliability*: To evaluate the reliability of the sulcal shape descriptors, we analyzed their variability, or reproducibility error, across the TRT sessions for each of the four TRT cohorts. For each MRI scan there are several sources of variability, including variability from hydration status, variability due to slightly different acquisitions in the two sessions (head position change in the scanner, motion artifacts, scanner instability, etc.), and finally variability due to the imaging processing methods themselves.

There could also be variability in the reliability estimates depending on the type of MRI system used (vendor, model, and acquisition parameters), so it is important to address the issue of reliability across a variety of platforms. We used two indices of reliability: (1) the dimensionless measure of absolute percent bias of descriptor, *b* (sulcal length, mean depth, width, and surface area) of a sulcus with respect to its average and (2) the ICC coefficient. *b* is computed as follows:

4 $$b = 100 \times \frac{{{\rm{test - retest}}}}{{\left( {{\rm{test + retest}}} \right)/2}}.$$

The estimation of the means is more robust than the estimation of the variance from the signed differences, in particular for smaller sets of subjects. The distributions of sulcal measurement differences plotted the mean across sessions were examined with a Bland–Altman analysis^110^. These plots show the spread of data, the bias (i.e., mean difference), and the limits of agreement (±1.96 SD), and were used to confirm that the distributions were approximately symmetric around 0 and to check for possible outliers. While the ICC estimates the relation between within-subject variance and between-subjects variance, *b* offers a subject-based index that might be used to find outliers. If scan and rescan are perfectly reliable, *b* should be equal to 0. The cases where *b* is >0.1, as in ref. ^49^, are considered unreliable.

The ICC coefficient was computed to quantify the reproducibility for sulcal-based measurements. ICC is defined as follows:

5 $${\rm{ICC}} = \frac{{\sigma _{\rm{BS}}^2}}{{\sigma _{\rm{BS}}^2 + \sigma _{\rm{WS}}^2}},$$

providing an adequate relation of within-subject (*σ*^2^_WS_) and between-subject (*σ*^2^_BS_) variability^111–113^.

The ICC estimates the proportion of total variance that is accounted for by the *σ*^2^_BS_. Values below 0.4 are typically classified as “poor” reproducibility, between 0.4 and 0.75 as “fair to good,” and higher values as “excellent” reproducibility^48^.

Equation (5) was used to estimate the ICC for each sulcal descriptor, independently for each cohort. The four cohorts were then combined into a meta-analysis (ICC_MA−SE_), similar to Eq. (1), in order to account for intra-site variability end to better estimate the sulcal reliability:

6 $${{\rm{ICC}}_{{\rm{MA}} - {\rm{SE}}}}(S) = \frac{{\mathop {\sum }\nolimits_j se_j^{ - 2} \times {\rm{ICC}}(S)}}{{\mathop {\sum }\nolimits_j se_j^{ - 2}}},$$

where *j* = 1, 2, 3, 4 indexes the cohorts. The SE was computed like SE = ICC/*Z*, where *Z* is obtained from a normal distribution knowing the *p* value. ${{\rm{ICC}}_{{\rm{MA}} - {\rm{SE}}}}$ was computed only if the cohort-based ICC computed with Eq. (5) was estimated for at least 3/4 cohorts.

### Reporting summary

Further information on research design is available in the Nature Research Reporting Summary linked to this article.

## Supplementary information

Supplementary Information

Supplementary Data

Peer Review File

Reporting Summary

Description of Additional Supplementary Files

## Data Availability

OASIS: the OASIS data are distributed to the greater scientific community under the Creative Commons Attribution 4.0 license. All data are available via www.oasis-brains.org^92^. KKI (Kennedy Krieger Institute—Multimodal MRI Reproducibility Resource): open access: https://www.nitrc.org/projects/multimodal/^91^. QTIM: data from the QTIM cohort used in this paper can be applied for by contacting M.J.W. (margie.wright@uq.edu.au). Access to data by qualified investigators are subject to scientific and ethical review. Summary results from cohort QTIM are available as part of the supplementary data^52^. HCP: family status and other potentially sensitive information are part of the Restricted Data that is available only to qualified investigators after signing the Restricted Data Use Terms. Open access data (all imaging data and most of the behavioral data) are available to those who register and agree to the Open Access Data Use Terms. Restricted data elements that could be potentially used to identify subjects include family structure (twin or non-twin status and number of siblings); birth order; age by year; handedness; ethnicity and race; body height, weight, and BMI; and a number of other categories. Each qualified investigator wanting to use restricted data must apply for access and agree to the Restricted Data Use Terms (https://humanconnectome.org/study/hcp-young-adult/data-use-terms)^87^. GOBS: data from the GOBS cohort used in this paper can be applied for by contacting D.C.G. (david.glahn@childrens.harvard.edu) or J. Blangero (John.Blangero@utrgv.edu). Access to data by qualified investigators are subject to scientific and ethical review and must comply with the European Union General Data Protection Regulations (GDPR)/all relevant guidelines. The completion of a material transfer agreement (MTA) signed by an institutional official will be required. Summary results from cohort GOBS are available as part of the supplementary data. UK Biobank: access to data from the UK Biobank can be obtained by approved scientists through application with UK Biobank (www.ukbiobank.ac.uk/researchers)^90^.

## Code availability

The image processing protocol developed for this work is available at http://enigma.ini.usc.edu/protocols/imaging-protocols/ (ENIGMA-Sulci).
