# Supplementary material for: The reliability and heritability of cortical folds and their genetic correlations across hemispheres
Source: Commun Biol. 2020 Sep 15;3:510. doi: 10.1038/s42003-020-01163-1 (PMC7493906; doi:10.1038/s42003-020-01163-1)
Supplement: Supplementary file 1 — Supplementary Information [file 42003_2020_1163_MOESM1_ESM.pdf]

Supplementary Figures

Supplementary Figure 1: Left Hemisphere: ICC across **left** sulci for the 4 test-retest cohorts analyzed here. The red line corresponds to the meta-analysis (see Supplementary Data 2). Missing values correspond to descriptors that were to be measured by BrianVISA for more than half of the subjects in a cohort, such as the length of the left *insula* and the left posterior sub-central ramus of the lateral fissure (*F.C.L.r.sc.post.).* Measures for the anterior sub-central ramus of the lateral fissure (*F.C.L.r.sc.ant.*) and the diagonal ramus of the lateral fissure (*F.C.L.r.diag*) were missing for all the descriptors.

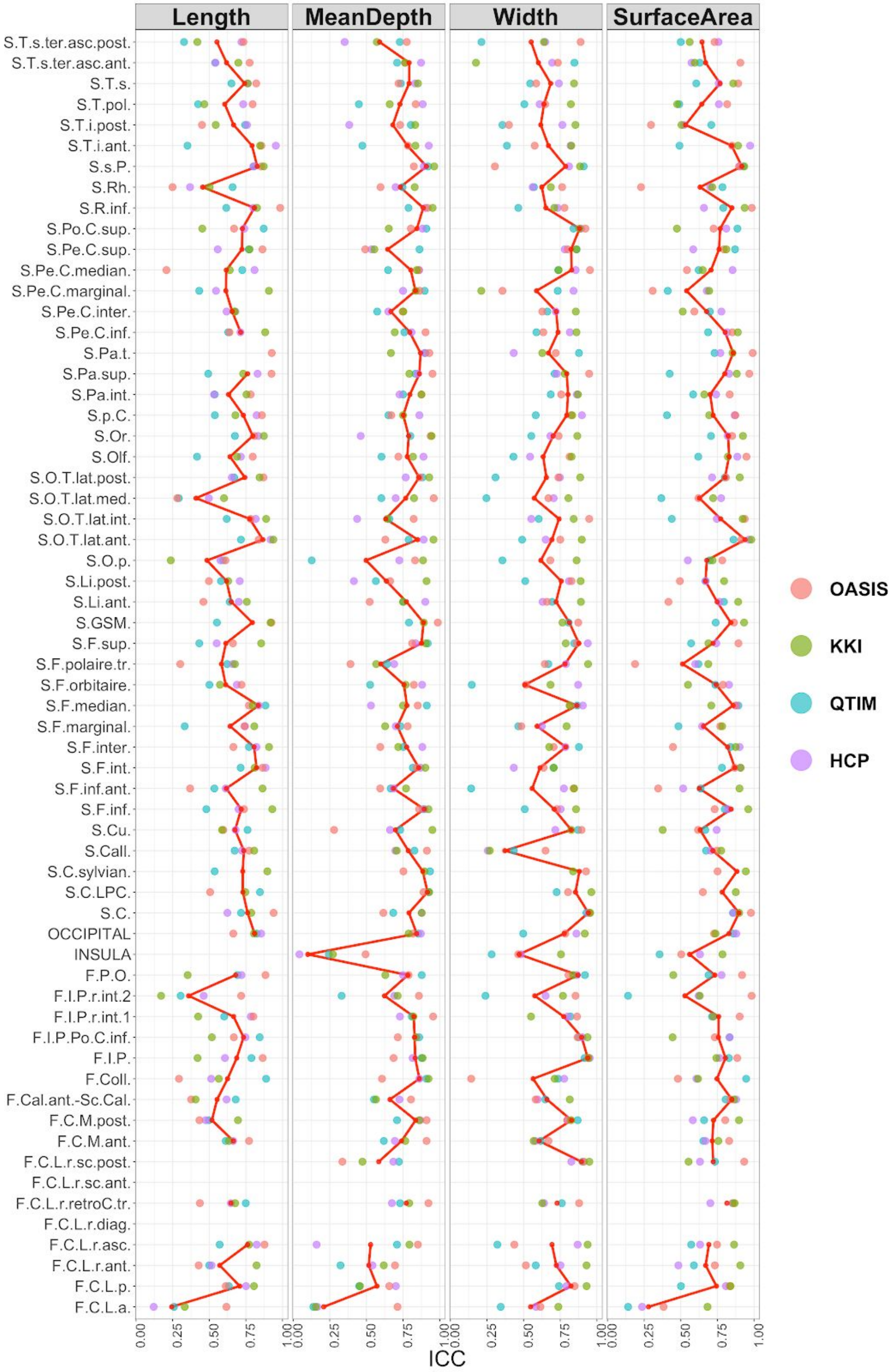

Supplementary Figure 2: Right Hemisphere: ICC across right sulci for the 4 test-retest cohorts analyzed here. The red line corresponds to the meta-analysis (see Supplementary Data 3). Missing values correspond to descriptors that were to be measured by BrianVISA for more than half of the subjects in a cohort, such as the length of the left *insula* and the left posterior sub-central ramus of the lateral fissure (*F.C.L.r.sc.post.)*. Measures for the anterior sub-central ramus of the lateral fissure (*F.C.L.r.sc.ant.*), and the paracentral lobule central sulcus (SC.LPC.). For the diagonal ramus of lateral fissure (*F.C.L.r.diag*) measures were missing for 4/5 of the cohorts and thus discarded for meta-analysis and left/right average, for all the descriptors.

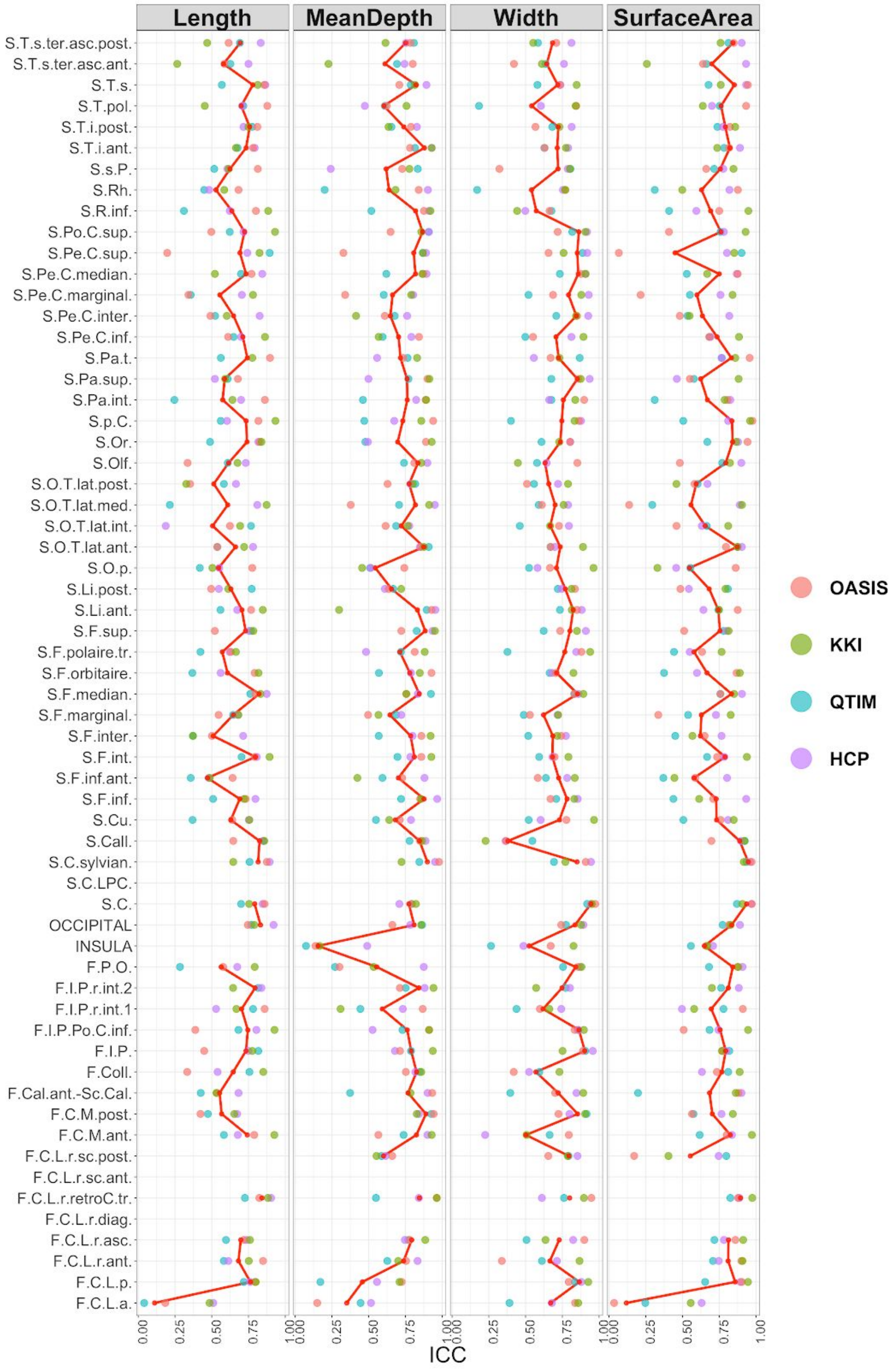

Supplementary Figure 3: Bilaterally averaged sulcal measure: ICC across sulci for the 4 test-retest cohorts analyzed here. The red line corresponds to the meta-analysis (see Supplementary Data 4). Missing values correspond to descriptors that were to be measured by BrianVISA for more than half of the subjects in a cohort, such as the length of the right *insula,* the paracentral lobule central sulcus (SC.LPC.), the anterior sub-central ramus of the lateral fissure (*F.C.L.r.sc.ant.*) and the diagonal ramus of lateral fissure (*F.C.L.r.diag*) for all the descriptors.

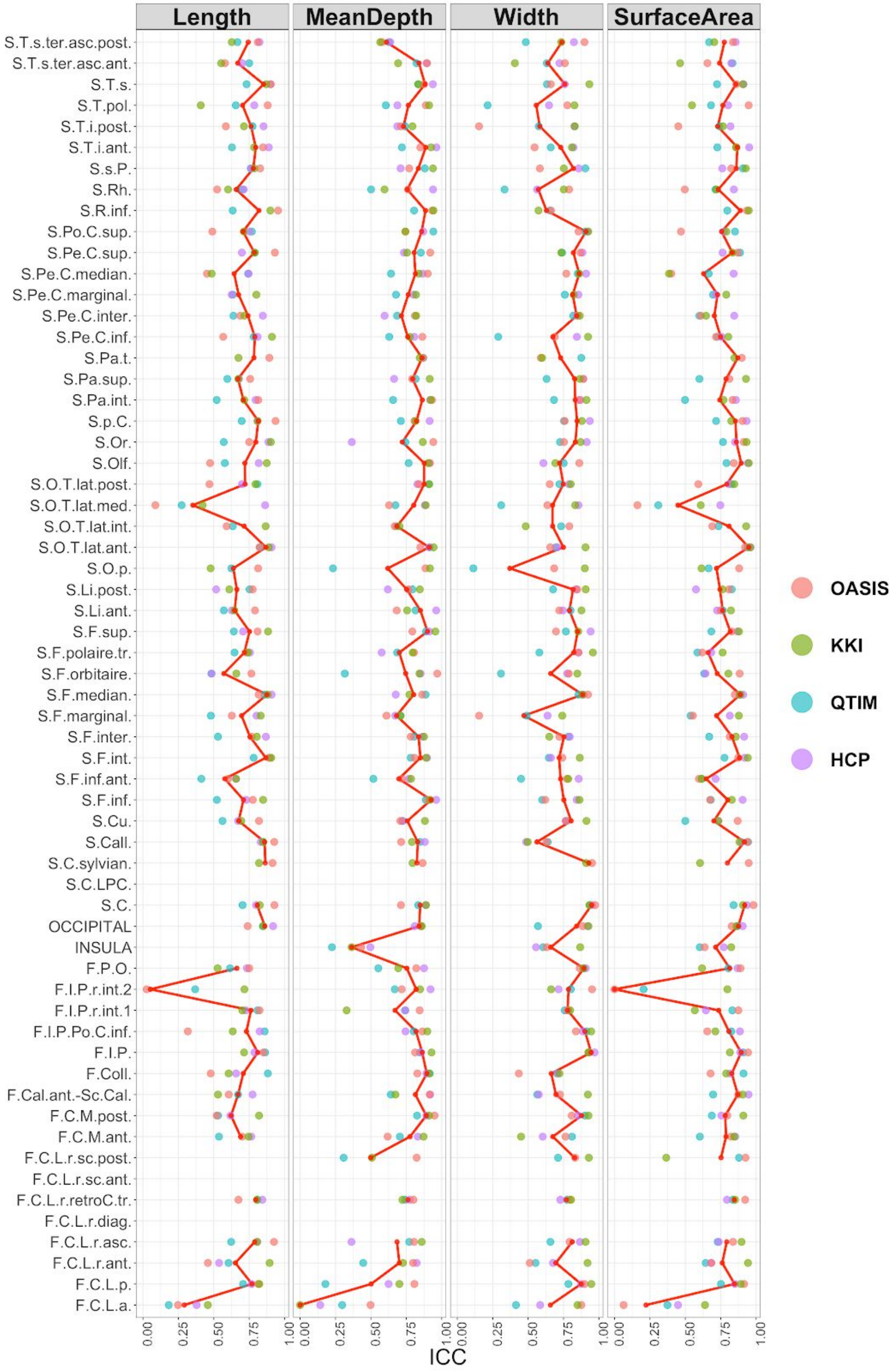

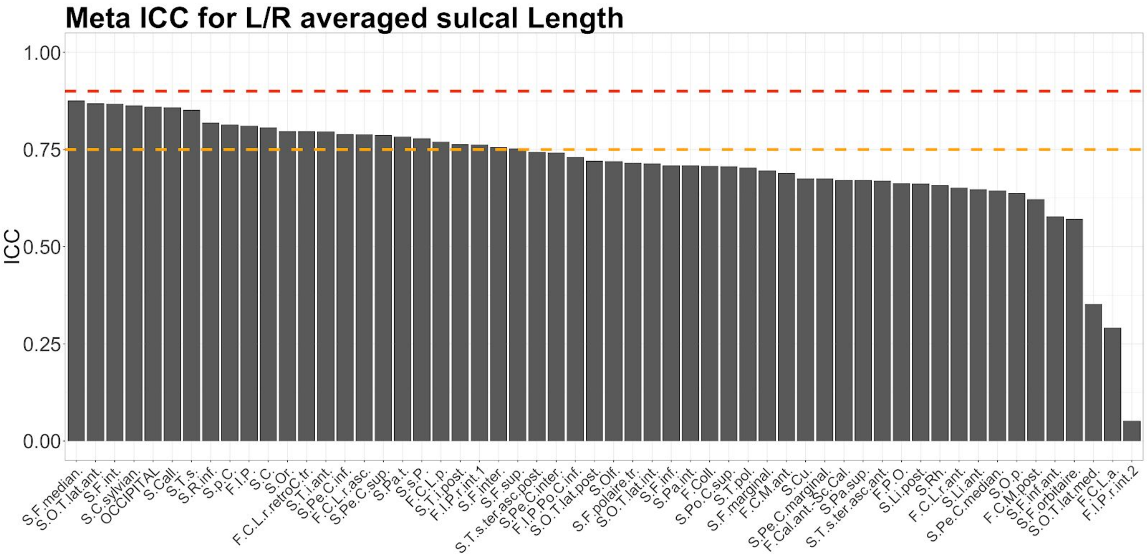

Supplementary Figure 4: Meta-Analysis: Ranked ICC for Left/Right averaged sulcal **Length**. The dashed orange and red lines highlight the 'good' (ICC=0.75) and 'excellent' (ICC=0.9) reliability thresholds [1]. (see Figure 1 and Supplementary Data 4).

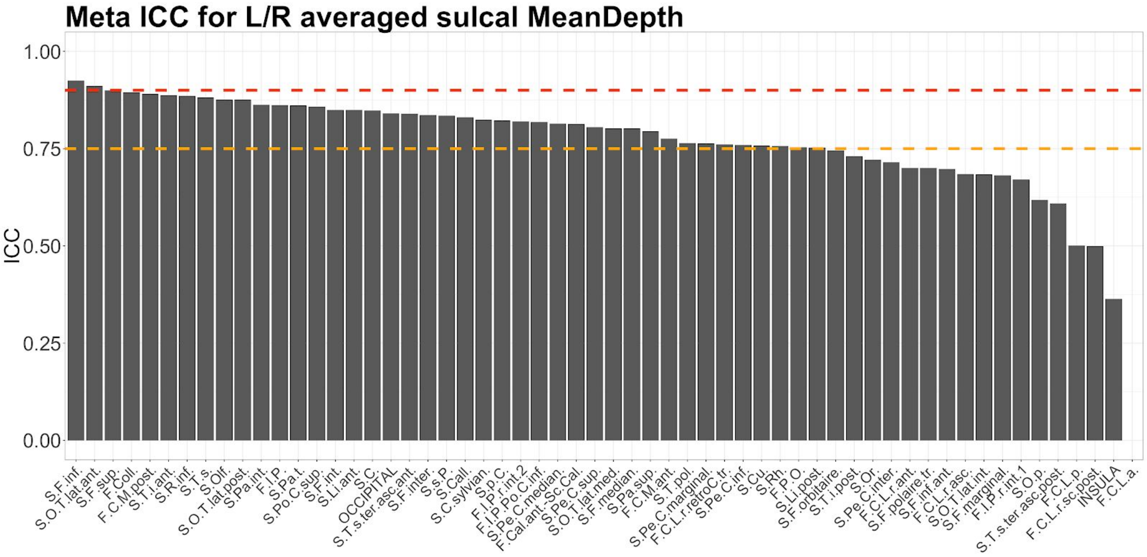

Supplementary Figure 5: Meta-Analysis: Ranked ICC for Left/Right averaged sulcal **Mean Depth**. The dashed orange and red lines highlight the 'good' (ICC=0.75) and 'excellent' (ICC=0.9) reliability thresholds [1]. (see Supplementary Data 4).

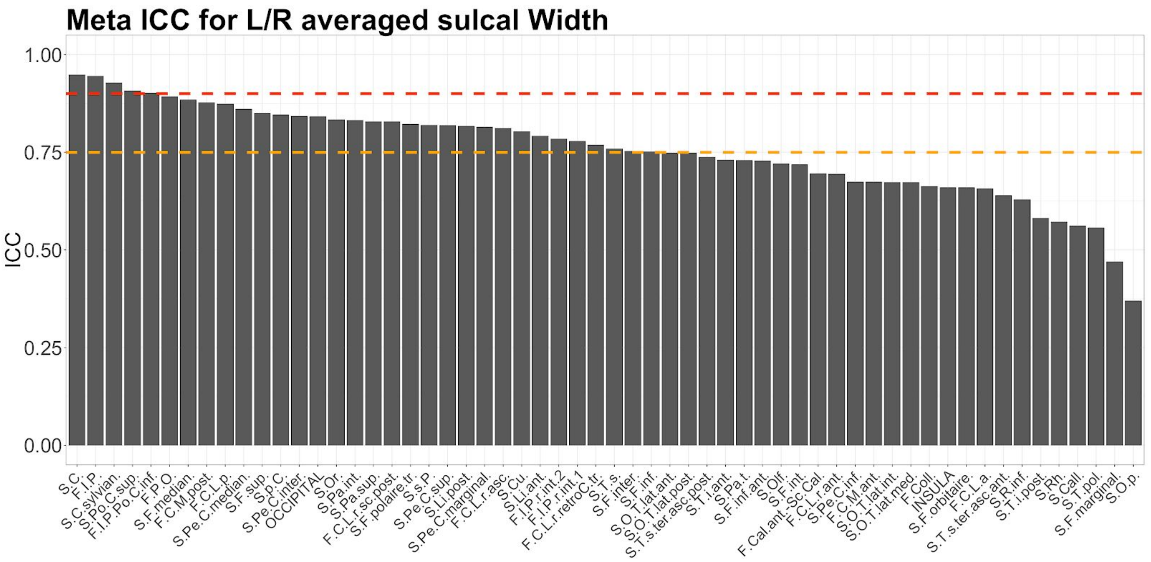

Supplementary Figure 6: Meta-Analysis: Ranked ICC for Left/Right averaged sulcal **Width**. The dashed orange and red lines highlight the ‘good’ (ICC=0.75) and ‘excellent’ (ICC=0.9) reliability thresholds [1]. (see Supplementary Data 4).

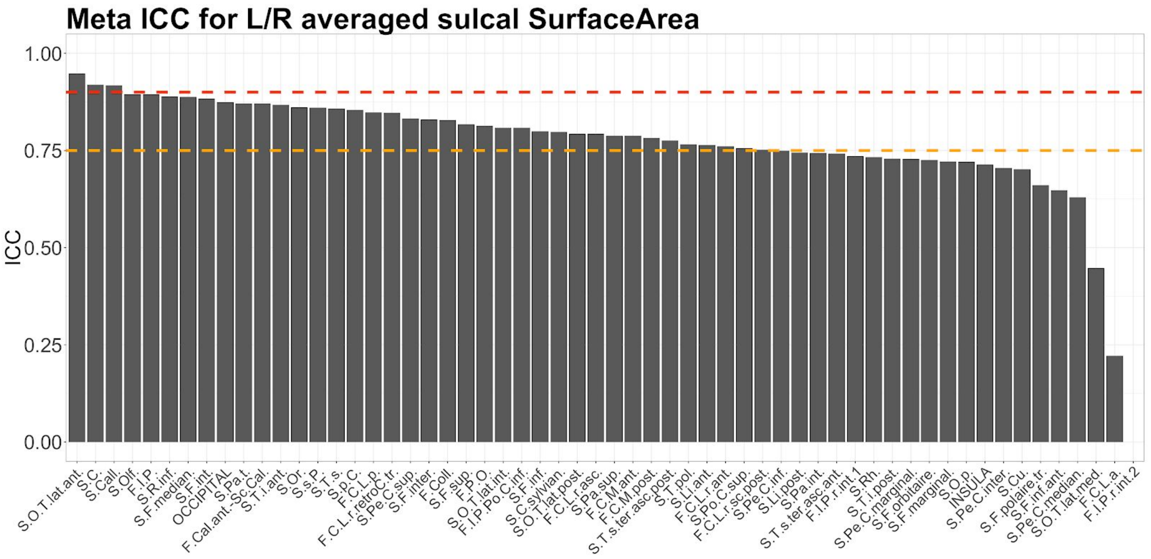

Supplementary Figure 7: Meta-Analysis: Ranked ICC for Left/Right averaged sulcal **Surface Area**. The dashed orange and red lines highlight the ‘good’ (ICC=0.75) and ‘excellent’ (ICC=0.9) reliability thresholds [1]. (see Supplementary Data 4).

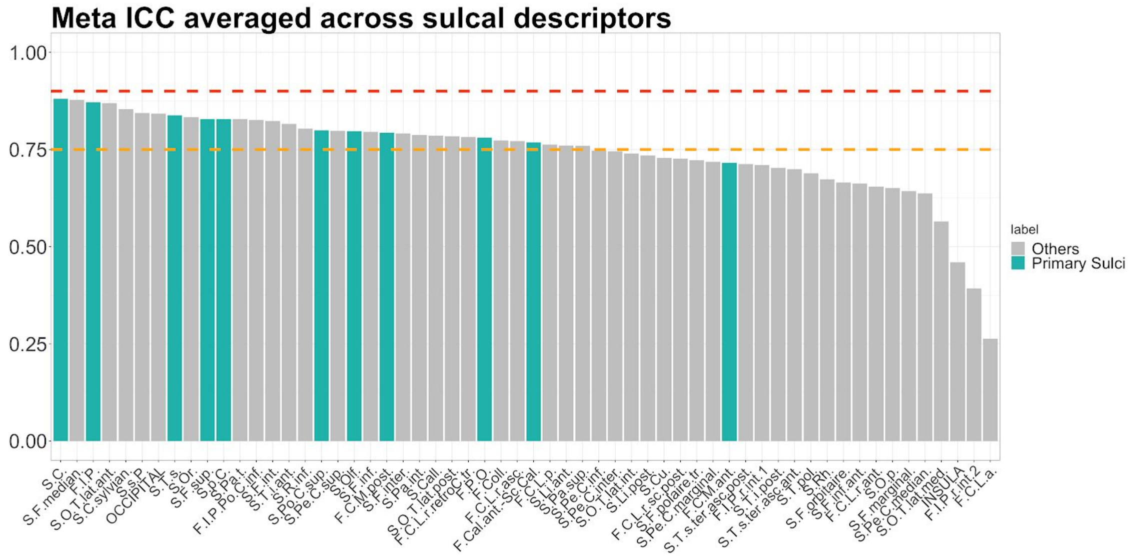

Supplementary Figure 8: Meta-Analysis: Average across sulcal shape descriptors of meta-ICC computed for bilaterally averaged sulcal measures. Primary sulci are highlighted in green. The dashed orange and red lines highlight the 'good' (ICC=0.75) and 'excellent' (ICC=0.9) reliability thresholds [1]. On average the central sulcus (*S.C.*) seems the more reliable and the anterior lateral sulcus (*F.C.L.a.*) the less reliable.

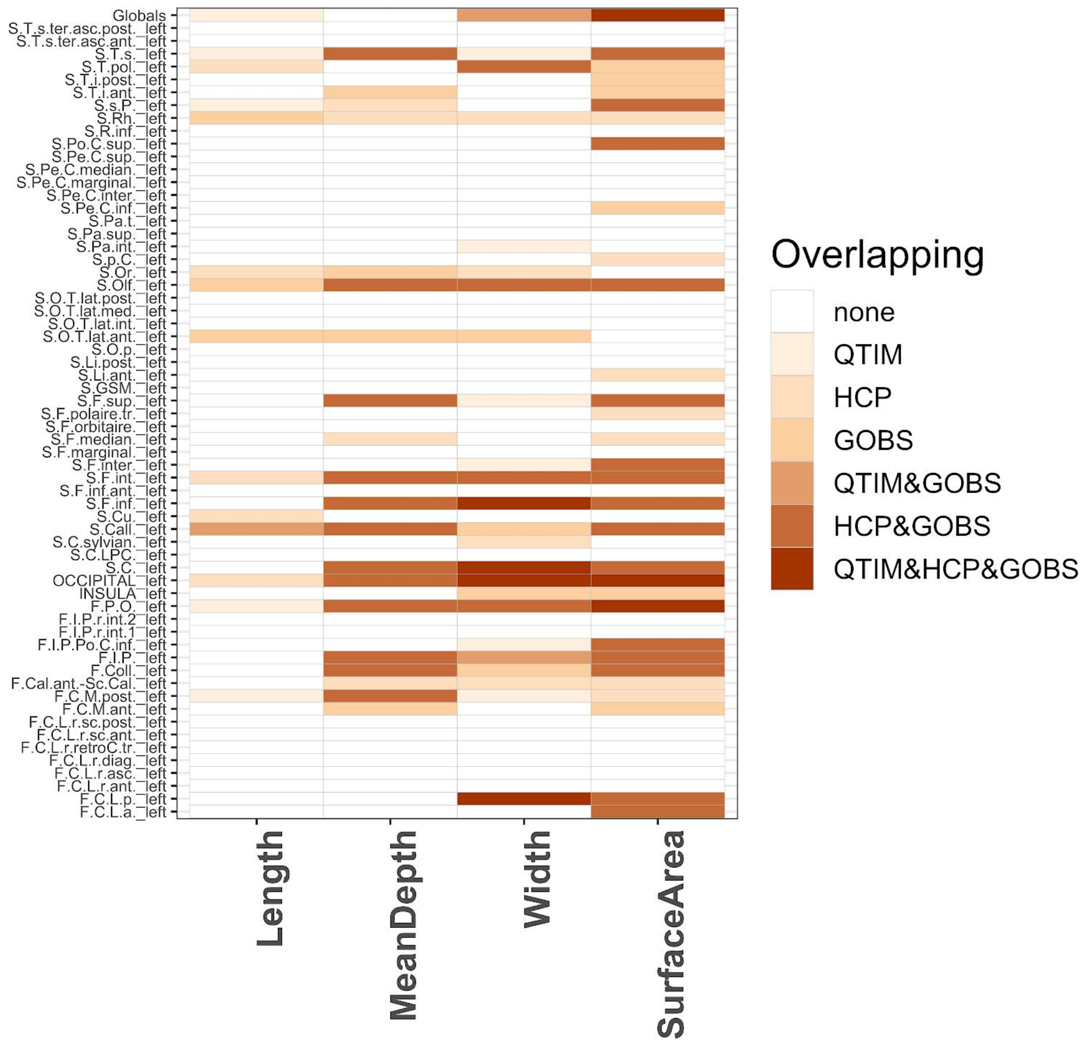

Supplementary Figure 9: Sulci showing univariate h^2 overlap between QTIM HCP and GOBS **univariate h2** for the **left** hemisphere. Only the *Bonferroni* corrected results are reported. No overlap was found between QTIM and HCP only. Among others, regions like the *left central sulcus* (S.C._left) and the *left occipital area* (OCCIPITAL_left) show significant heritability for sulcal width for the three cohorts.

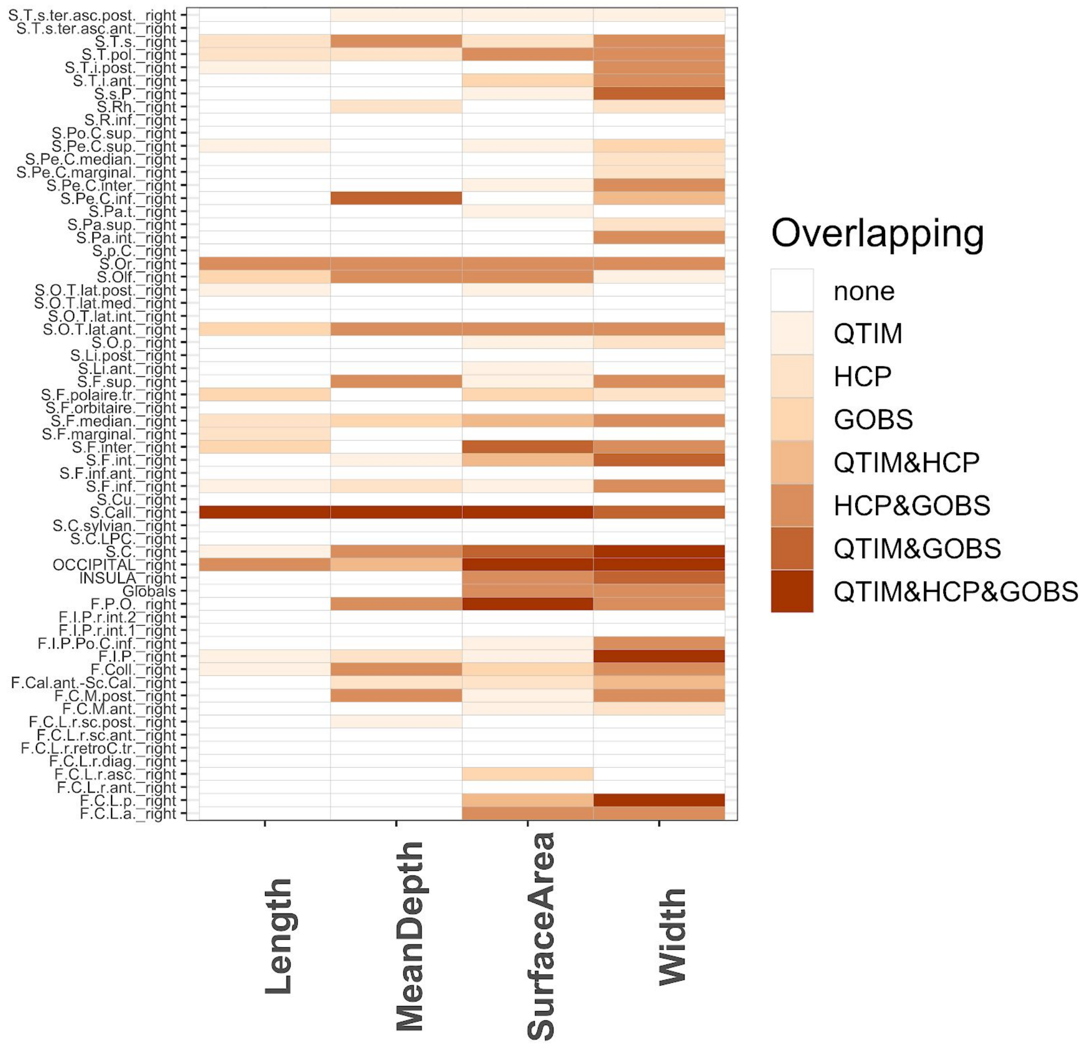

Supplementary Figure 10: Sulci showing univariate h^2 overlap between QTIM HCP and GOBS **univariate h2** for the **right** hemisphere. Only the *Bonferroni* corrected results are reported. Among others, regions like the *right subcallosal sulcus* (S.Call._right) and the *right parieto-occipital fissure* (F.P.O._right) show significant heritability for sulcal length/mean depth and surface area the former, and the surfer area the latter, for the three cohorts.

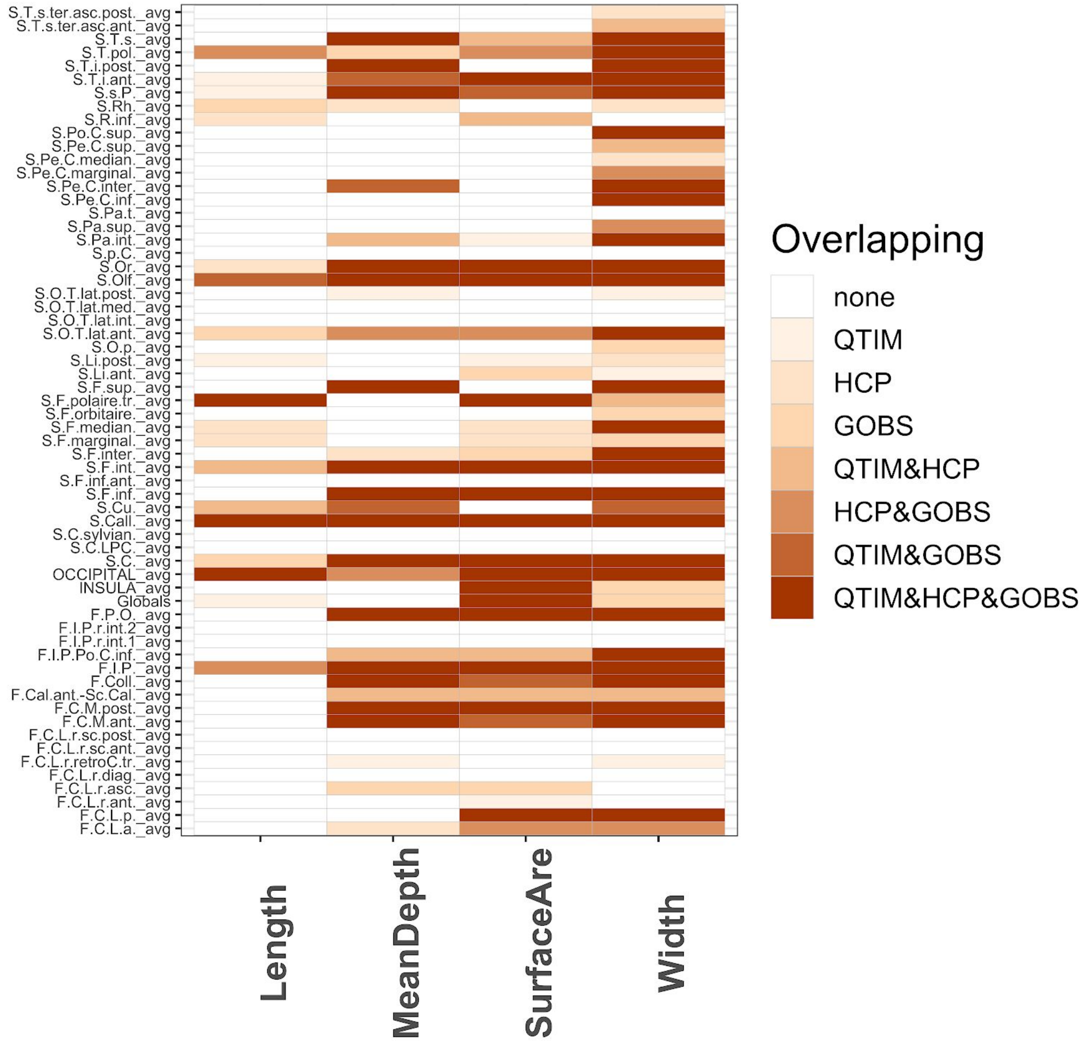

Supplementary Figure 11: Sulci showing univariate h^2 overlap between QTIM HCP and GOBS **univariate h2** for the **bilaterally averaged sulci.** Only the *Bonferroni* corrected results are reported. Among others, regions like the *right subcallosal sulcus* (S.Call._right) and the *right parieto-occipital fissure* (F.P.O._right) show significant heritability for sulcal length/mean depth and surface area the former, and the surfer area the latter, for the three cohorts.

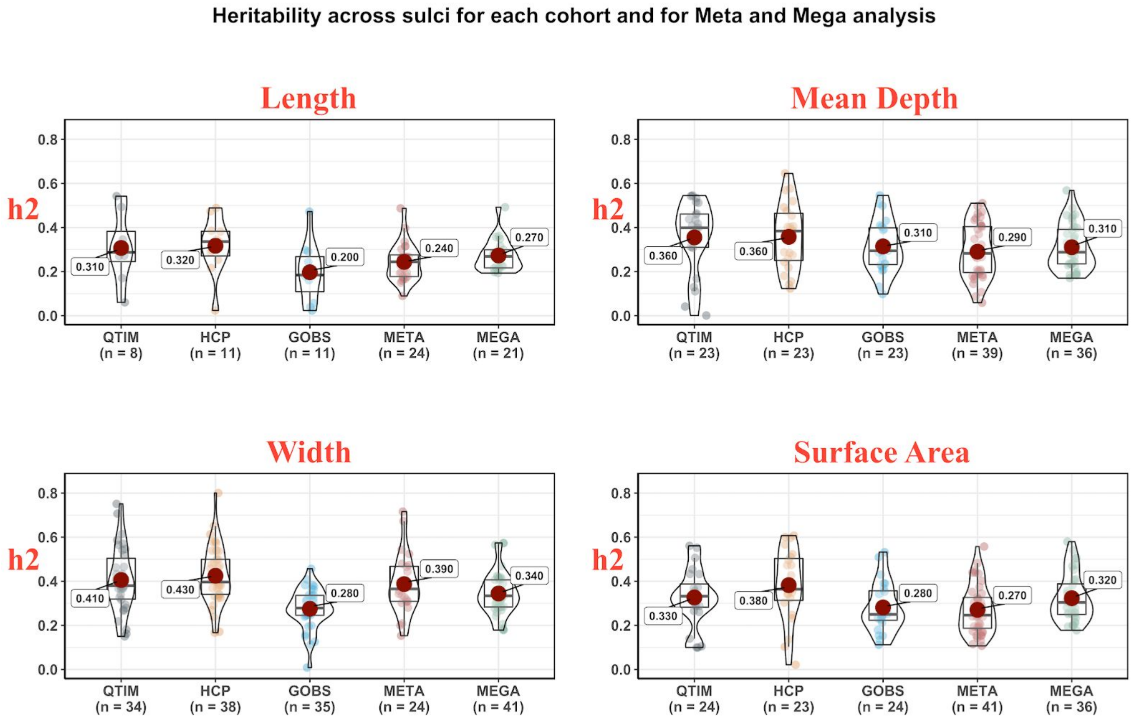

Supplementary Figure 12: Violin/box plots of sulcal-based heritability (h2) for the bilaterally average sulcal descriptors, in QTIM, HCP, GOBS and for the meta- and mega-analyses. The average h^2 value is reported with the number of sulci surviving *Bonferroni* correction between parentheses.

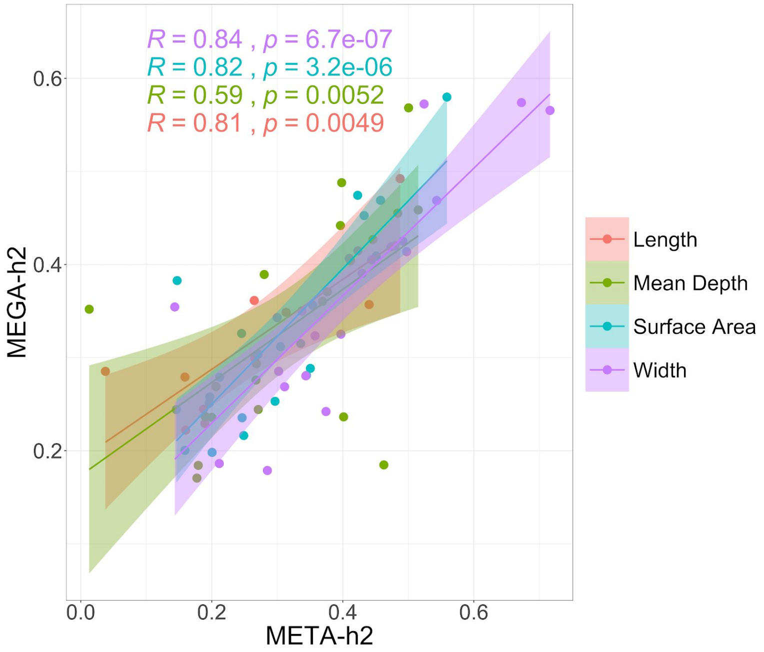

Supplementary Figure 13: Scatter plots with Pearson's correlation coefficients estimated between Meta and Mega analysis, for sulcal length, mean depth, surface area, and width. The correlation has been computed for the measures surviving *Bonferroni* correction [pval ≤ 0.05/(4x61)] and ICC > 0.75. Dof: 8, 19, 20, 21 for length, mean depth, surface area and width, respectively.

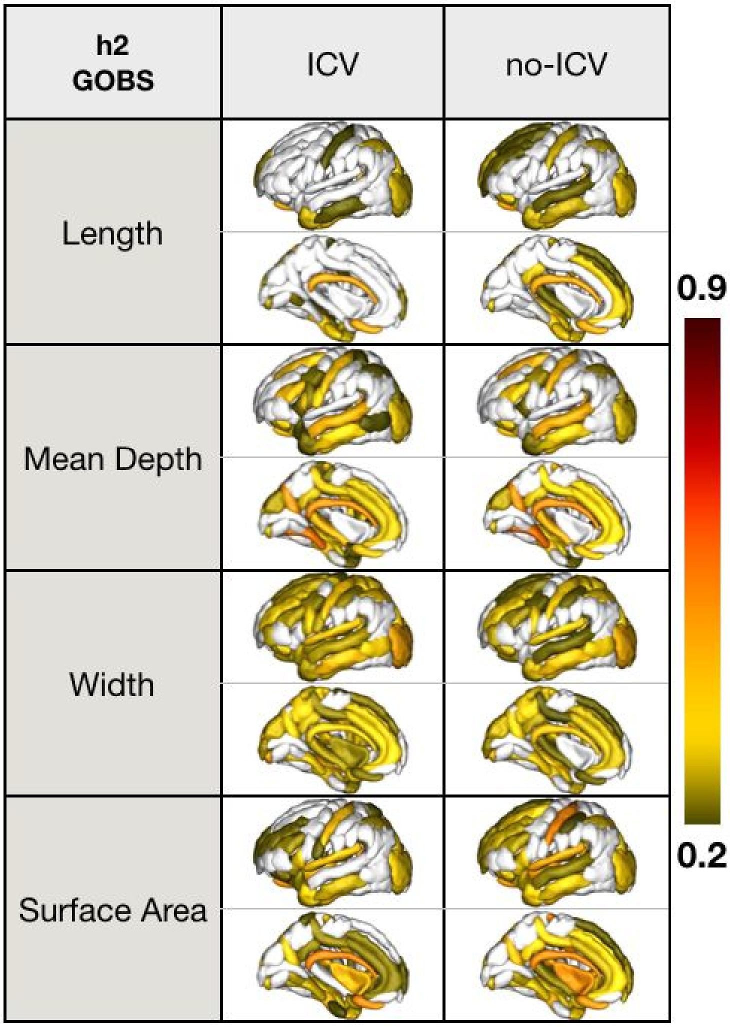

Supplementary Figure 14: Sulcal-based h2 for length, mean depth, surface area and width, for GOBS, controlling for ICV (left) and without ICV (right). The genetic influence over many sulcal lengths appears to be driven by ICV; as surface area is also a function of the length, this pattern is also seen there. Covarying for ICV has relatively no effect on average sulcal depth or width.

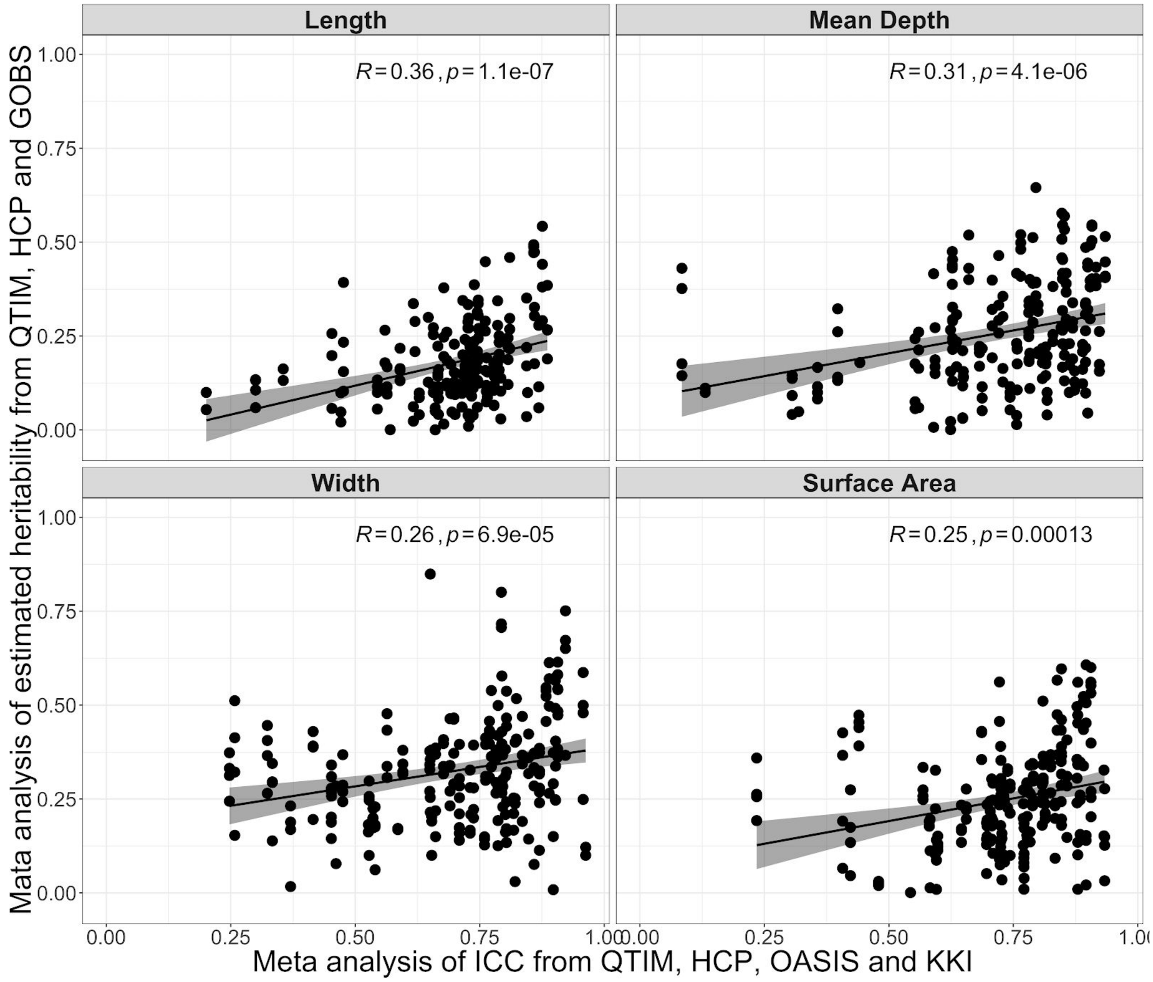

Supplementary Figure 15: Scatter plots with Pearson's correlation coefficients estimated between Meta-h2 and Meta-ICC analysis, for bilaterally averaged measures of sulcal length, mean depth, surface area, and width.

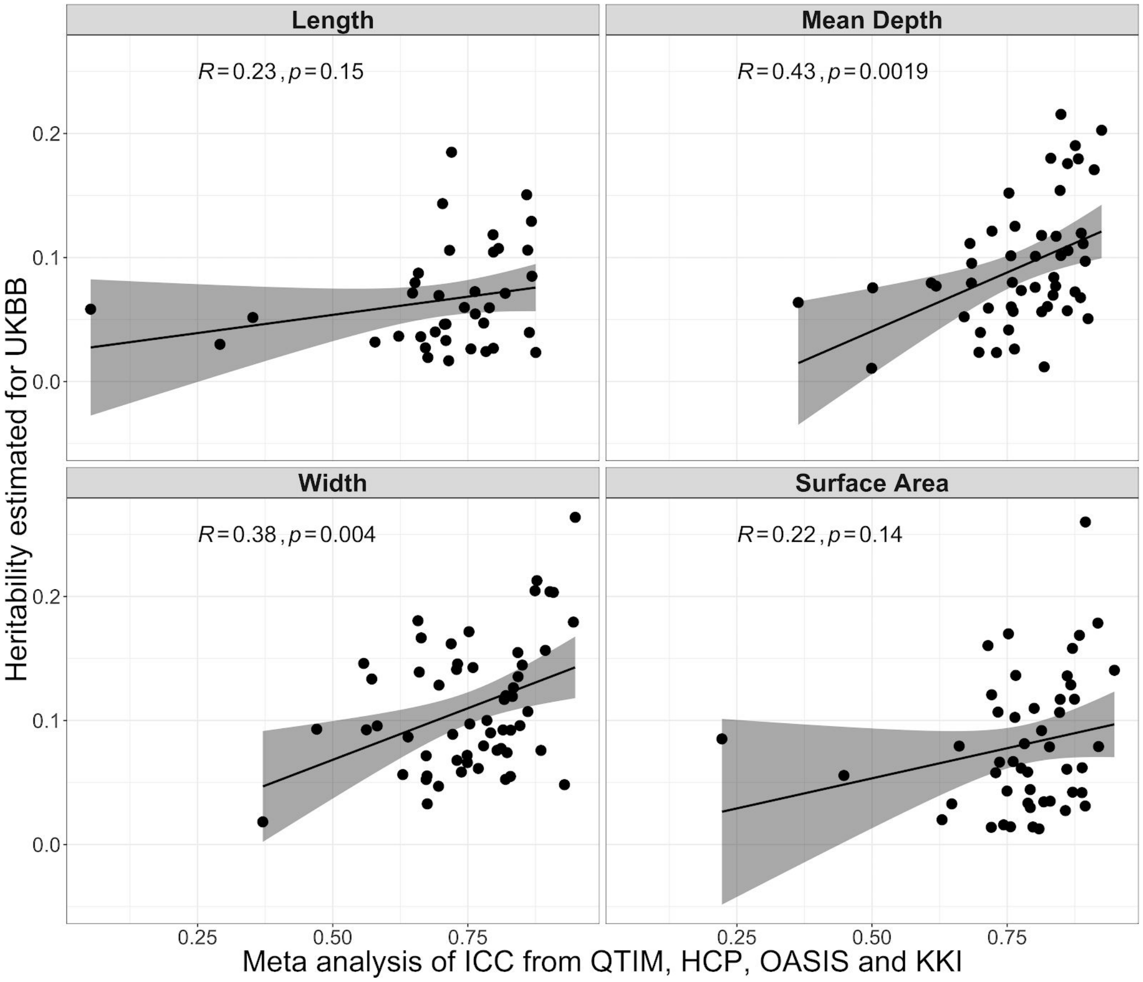

Supplementary Figure 16: Scatter plots with Pearson's correlation coefficients estimated between h2 estimated in UKBB and Meta-ICC analysis, for bilaterally averaged measures of sulcal length, mean depth, surface area, and width.

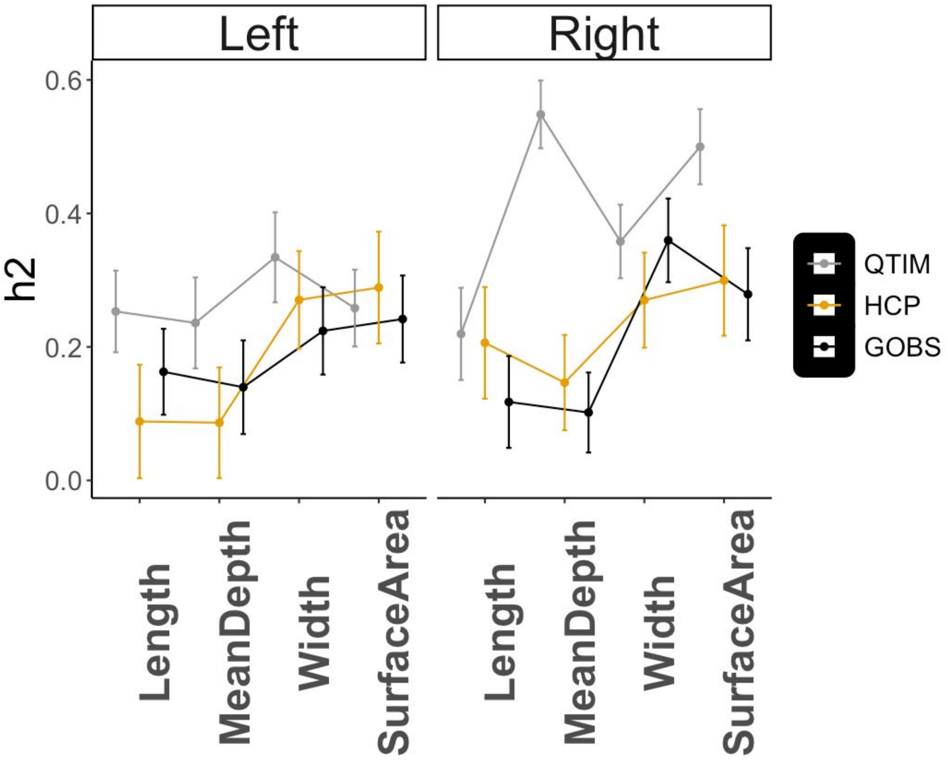

Supplementary Figure 17: Univariate heritability (h^2) with standard error of global sulcal shape descriptor, for left and right hemisphere. The heritability was computed on the sum of sulcal length, mean depth, width and surface area across left and right sulci. QTIM, HCP and GOBS show similar trends across descriptor and hemispheres, except QTIM which seems to have higher heritability for the right hemisphere comparing to the left one.

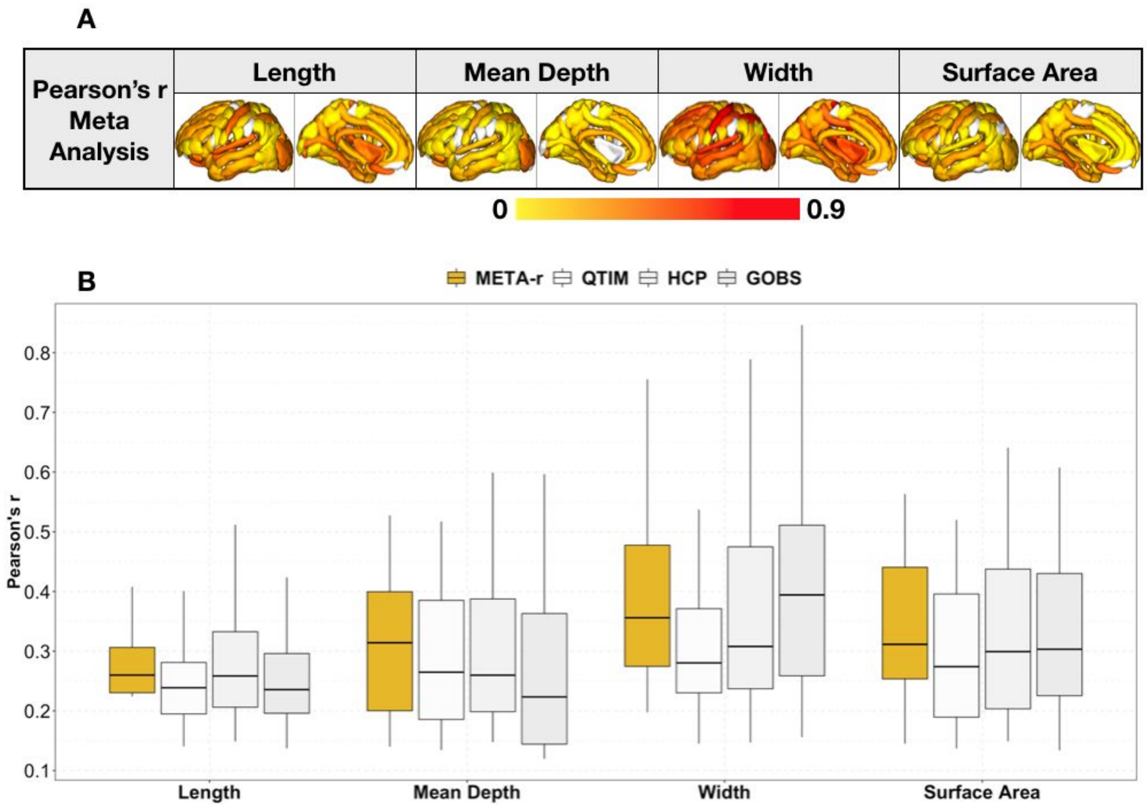

Supplementary Figure 18: A) **Meta-analysis** of Pearson's correlation between left and right sulcal length, mean depth, surface area and width. B) Boxplots of Pearson's correlation between left and right sulcal measures for HCP, QTIM, GOBS and for the meta analysis (META-r) as mapped in A).

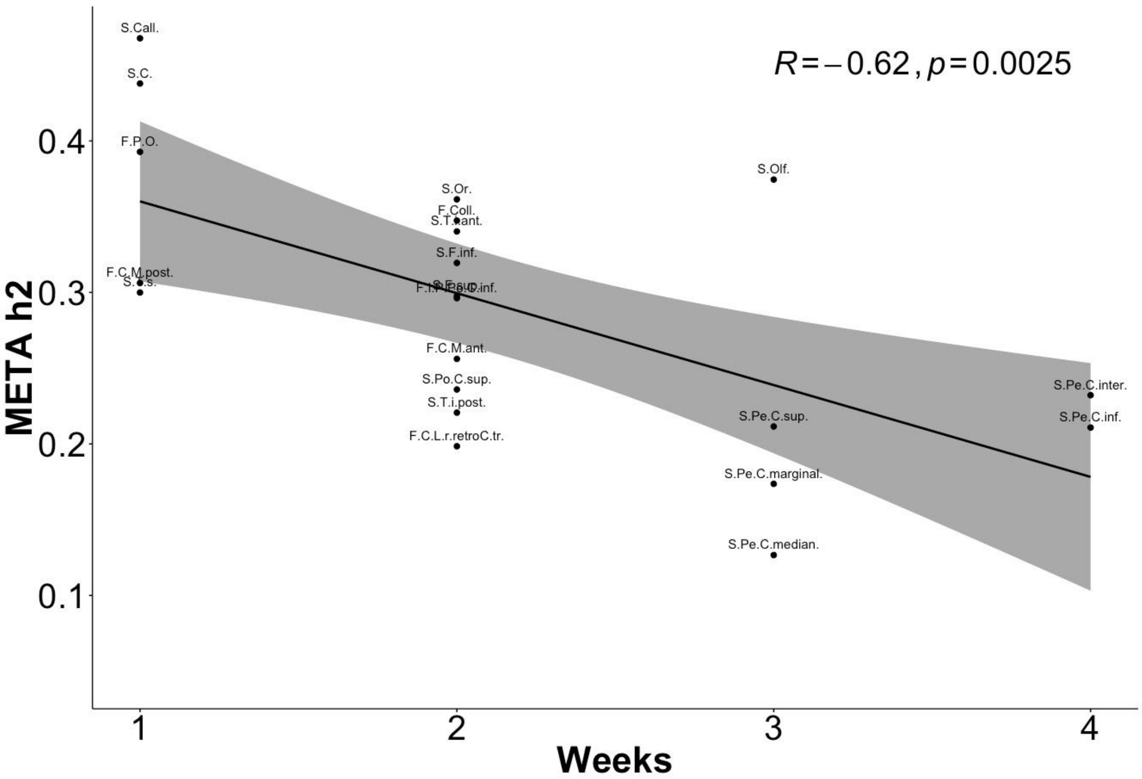

Supplementary Figure 19: Pearson's correlation between heritability (h^2) and the appearance of sulci (in weeks). From Dubois et al. 2018 we grouped the sulci in four groups, 26.7w ("1"), 31.0w ("2"), 34.0w ("3") and 35.7w ("4") [2]. h^2 here has been computed as the average of the heritability of sulca length, mean depth, width and surface area, as estimated by the meta analysis of the bilaterally averaged shape measures. The negative correlation (r=-0.62, p=0.0025) suggests that sulci appearing early in brain development are those showing higher estimated heritability.

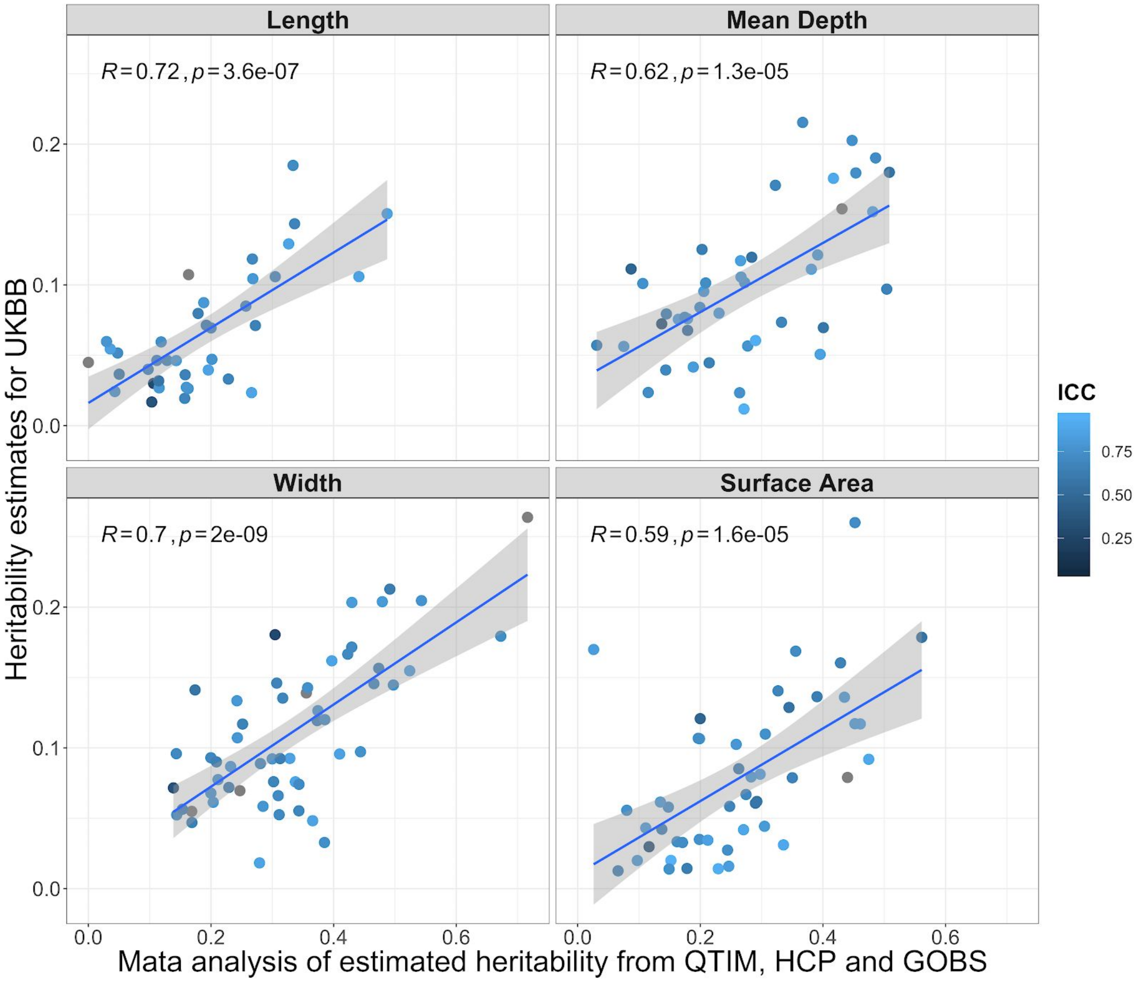

Supplementary Figure 20: Pearson's correlation between heritability (h^2) estimated from UKBB sulcal shape descriptors and the meta analysis of heritability estimated from QTIM, HCP and GOBS. The scatter points are colored based on the ICC computed pooling together 4 test-retests. The results reported here refer to the bilaterally averaged measures of sulcal length, mean depth, width and surface area.

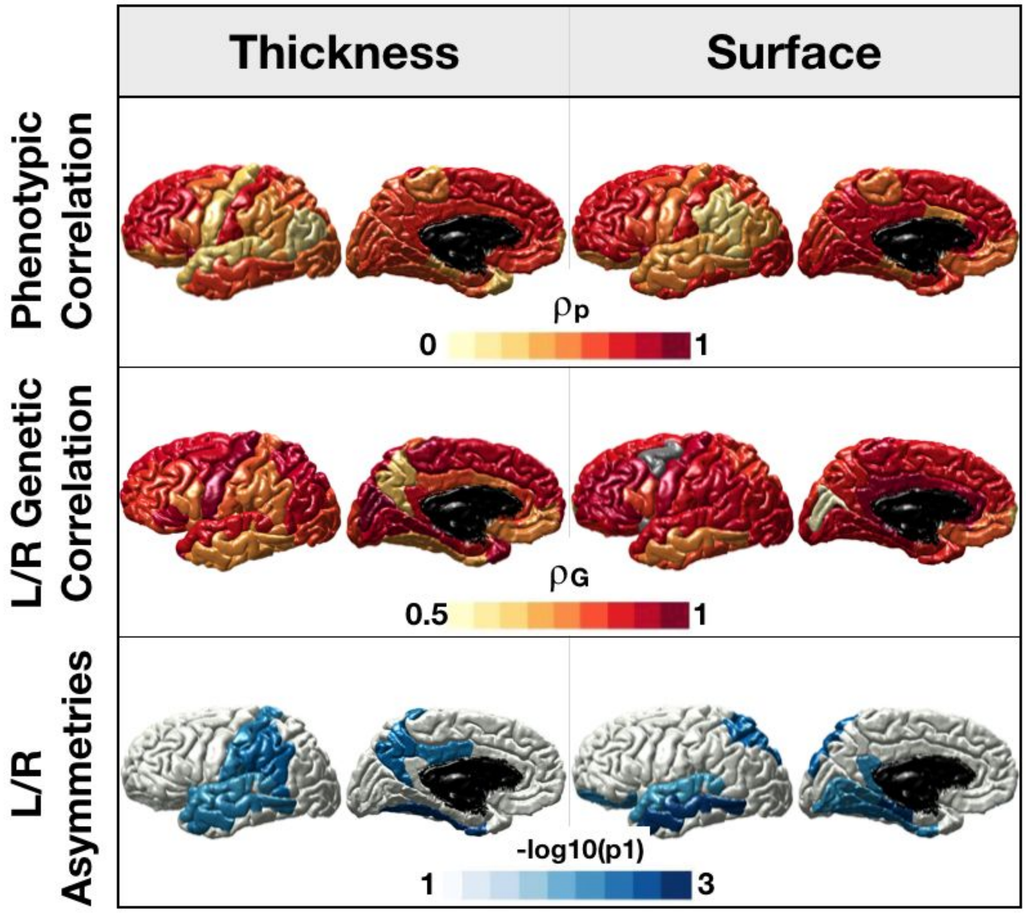

Supplementary Figure 21: Top and middle rows show respectively the meta-analysis of bilaterally phenotypic correlation and the genetic correlation (equation 1 in main text) between left and right thickness and surface values extracted from FreeSurfer ROIs (Desikan-Killiany atlas [3]). For the meta-analysis only QTIM and HCP datasets have been used. The bottom row shows those regions for which the 95% confidence interval for genetic correlation did not include 1, identifying regions that show potential genetic asymmetries (p1 is the p-value when testing for differences from 1); our results may extend previous findings for cortical thickness of temporal lobe and postcentral gyrus and cortical surface of the temporal lobe and superior parietal lobe [4,5]. The results reported are *Bonferroni* corrected.

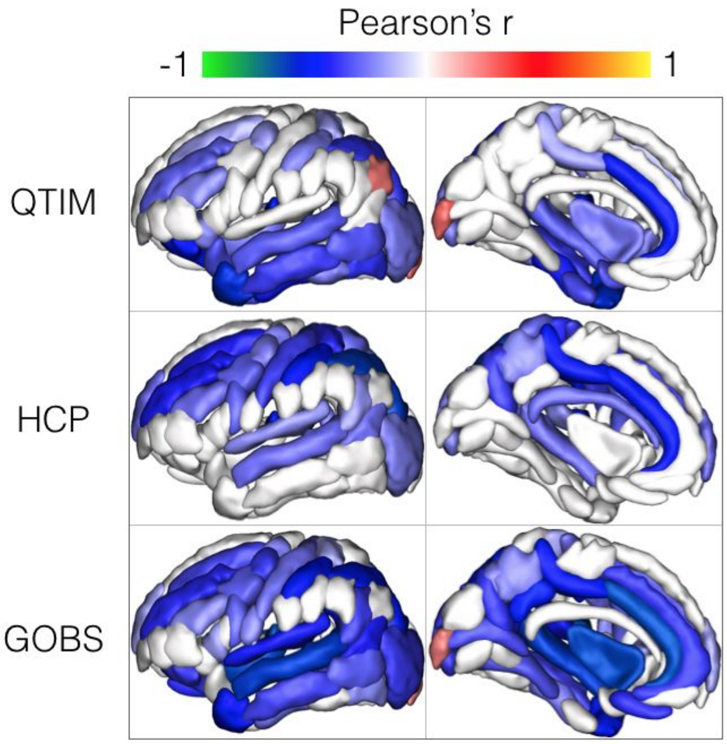

Supplementary Figure 22: Pearson's correlation between sulcal width and grey matter thickness (GM), for QTIM, HCP and GOBS. For each sulcus the surrounding grey matter thickness has been estimated using BrainVISA Morphologist pipeline. A negative correlation between GM thickness and sulcal width suggests a differential trajectories of these two measures in brain development. For the cohorts analyzed here most of the sulci show a negative correlation (colorbar blue to green) between -0.2 and -0.6. Further investigation and longitudinal designs may better disentangle GM and sulcal with trajectories.

Supplementary Notes

Analysis of failing subjects for sulcal segmentation

We have estimated how many subjects failed for 2 or more sulci, separately for QTIM, HCP, GOBS and UKBB. If BrainVISA fails to identify a sulcus, no values are output for the sulcus, and we consider them "missing". On average, without considering the z-score filtering, four sulci are missing for the majority of subjects.. QTIM had a higher percent of sulcal segmentation errors (missing sulci) than either HCP,GOBS or UKBB, possibly due to the fact that it was collected at 4 tesla. This is consistent with previous work showing lower reliability for Freesurfer cortical measures in QTIM than HCP[6].

Here the details:

For QTIM, we analyzed data from 1,009 subjects, none of which failed entirely (no BrainVISA output). On average 4 sulci (st dev. 2) failed for each subject in QTIM, (**Supplementary Figure 23**). **Supplementary Figure 24** reports the number of subjects failing (in percentage of the total population) for left and right hemispheres.

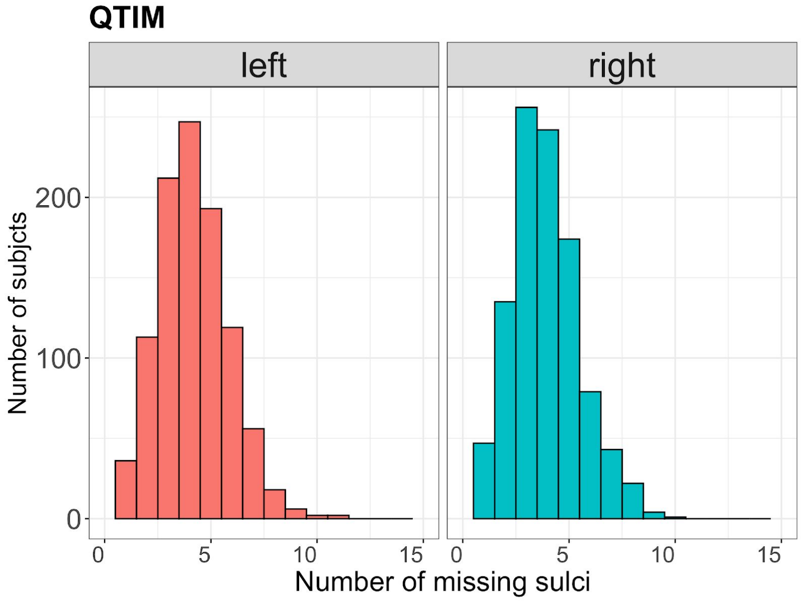

Supplementary ***Figure 23:*** *Histogram of number of subjects counted for the missing left and right sulci, in QTIM. The missing sulci are removed before outlier detection.*

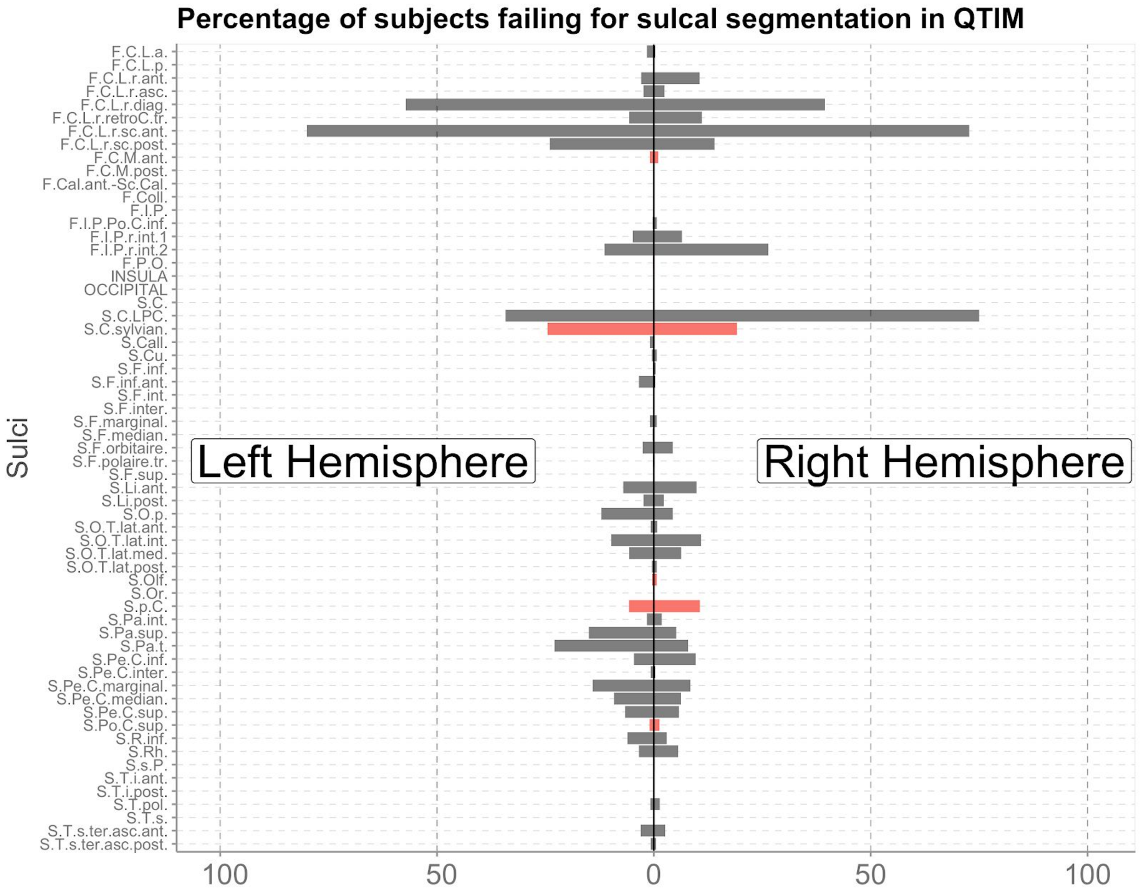

Supplementary ***Figure 24:*** *Percentage of subjects failing for each sulcus in QTIM. The primary sulci are highlighted in red.*

For HCP, 816 subjects were analyzed and no subject failed for the whole set of sulci. On average, HCP subjects failed for 4 sulci (st dev. 2) (**Supplementary Figure 25**). **Supplementary Figure 26** reports the number of subjects failing (in percentage of the total population) for left and right hemispheres.

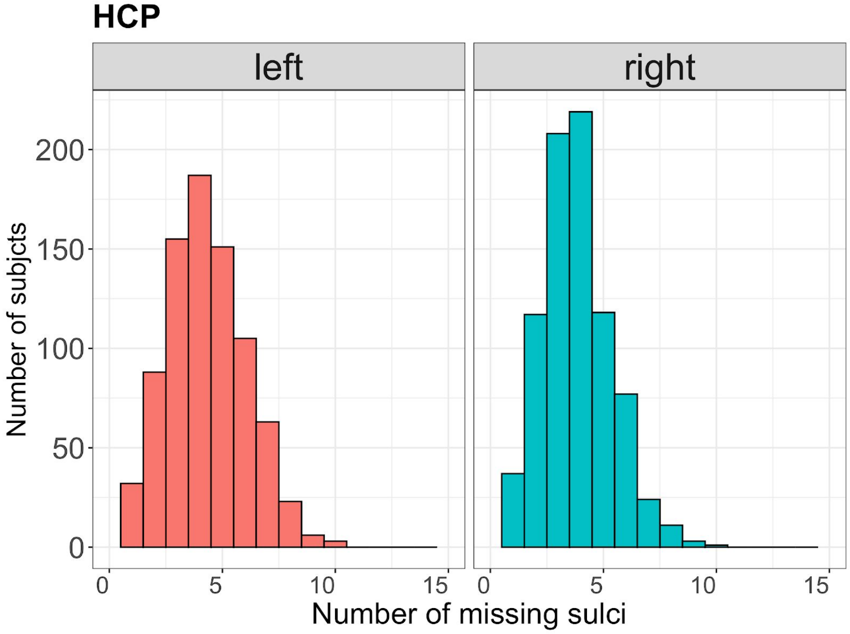

Supplementary *Figure 25:* *Histogram of number of subjects counted for missing left and right sulci, in the HCP dataset. The missing sulci are removed before outlier detection.*

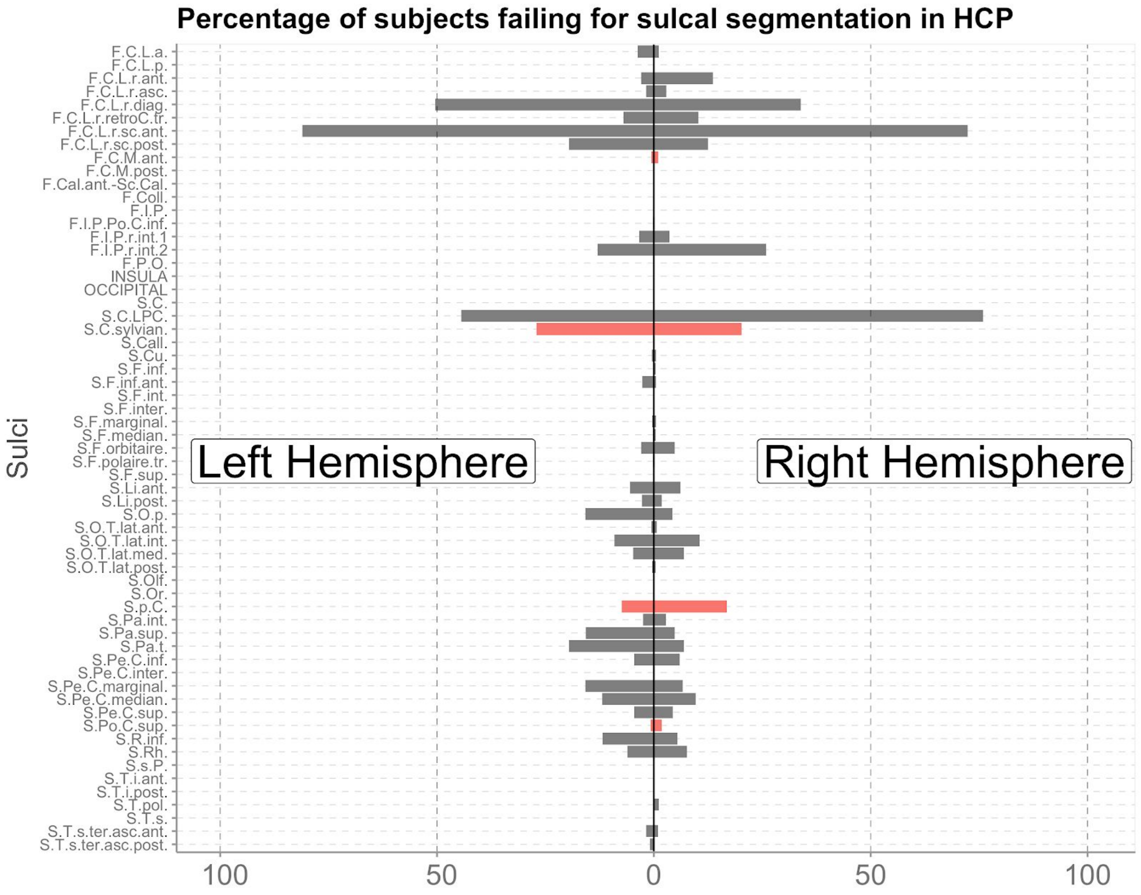

Supplementary ***Figure 26:*** *Percentage of subjects failing for each sulcus in HCP. The primary sulci are highlighted in red.*

For GOBS, data from 1,025 subjects were analyzed and no subject failed for the whole set of sulci. On average, GOBS subjects failed for 5 (st dev. 2) sulci (**Supplementary Figure 27**). **Supplementary Figure 28** reports the number of subjects failing (in percentage of the total population) for left and right hemispheres.

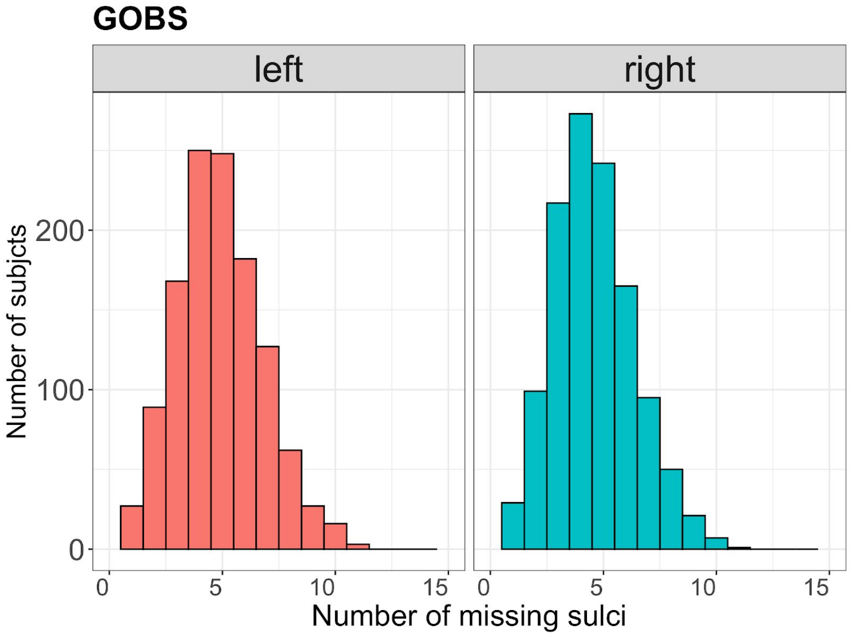

Supplementary *Figure 27:* *Histogram of number of subjects counted for missing left and right sulci, in the GOBS dataset. The missing sulci are removed before outlier detection.*

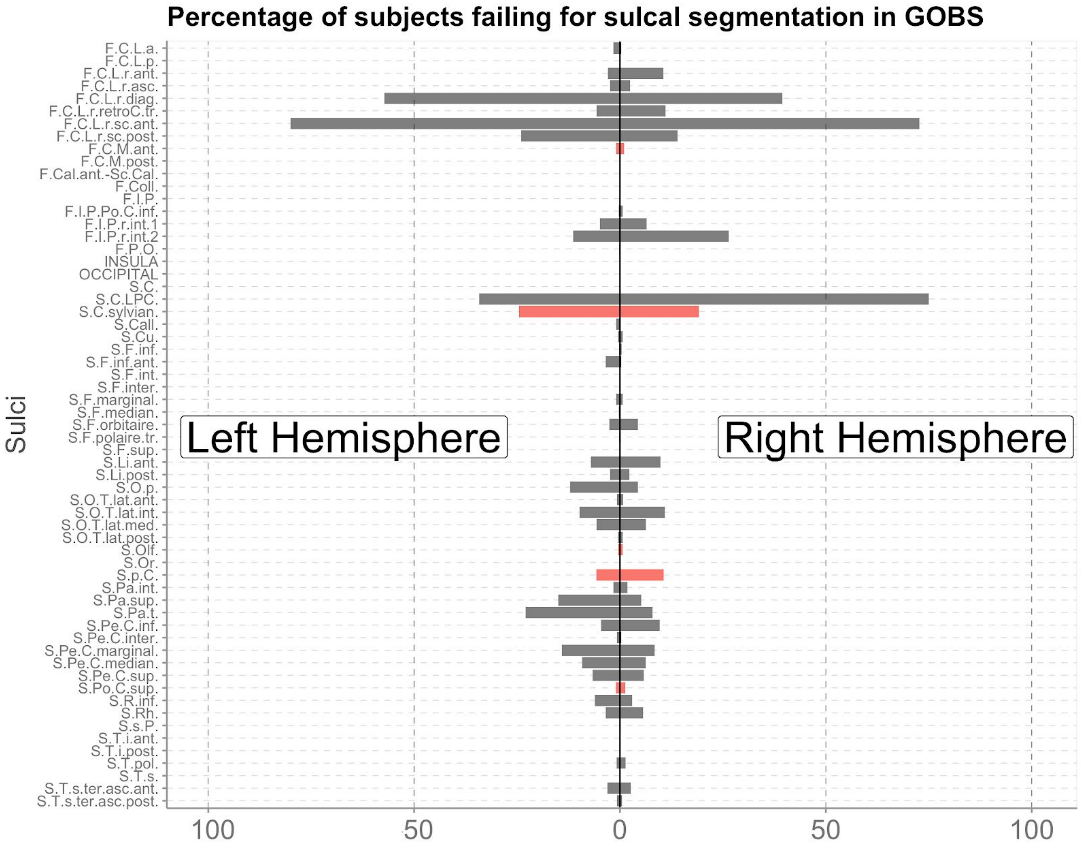

Supplementary *Figure 28:* *Percentage of subjects missing for each sulcus in the GOBS dataset. The primary sulci are highlighted in red.*

For UKBB, we analyzed data from 10,083 subjects. We found that 150 subjects failed for the whole set of sulci.. On average, UKBB subjects failed for 4 sulci (st dev. 2) (**Supplementary Figure 29**). **Supplementary Figure 20** reports the number of subjects failing (in percentage of the total population) for left and right hemispheres.

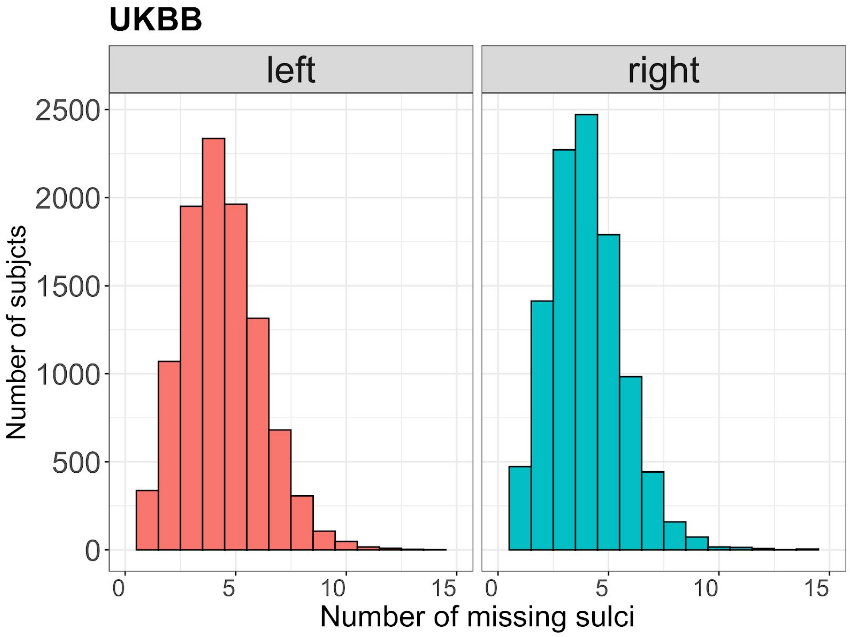

Supplementary ***Figure 29:*** *Histogram of number of subjects counted for the missing left and right sulci, in the UKBB dataset. The missing sulci are removed before outlier detection.*

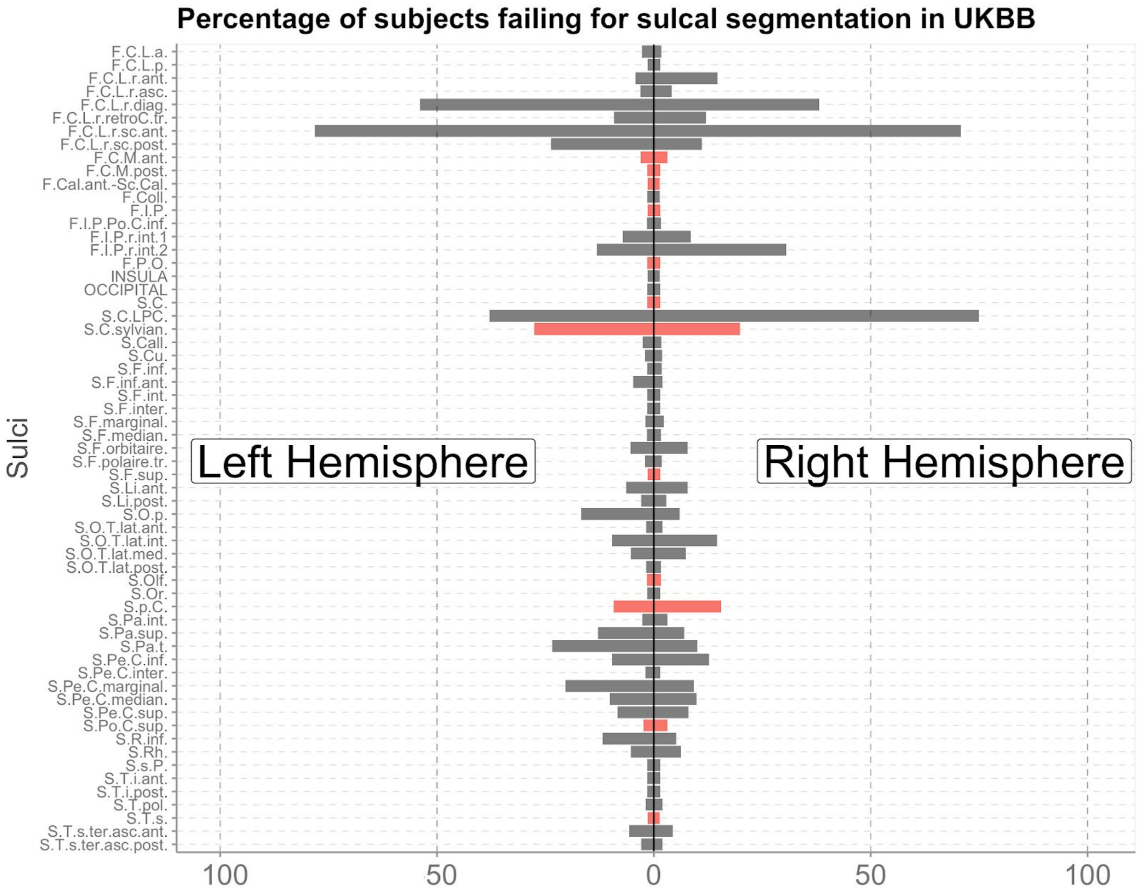

Supplementary *Figure 30:* *Percentage of subjects missing for each sulcus in the UKBB dataset. The primary sulci are highlighted in red.*

We assessed the correlation between the number of failed subjects and the ICC for each bilaterally averaged sulcal measure; we found no correlation between ICC and the number of failed subjects (**Supplementary Figure 31**). **Supplementary Figure 31** shows the correlation between the number of failed subjects and ICC for sulcal width.

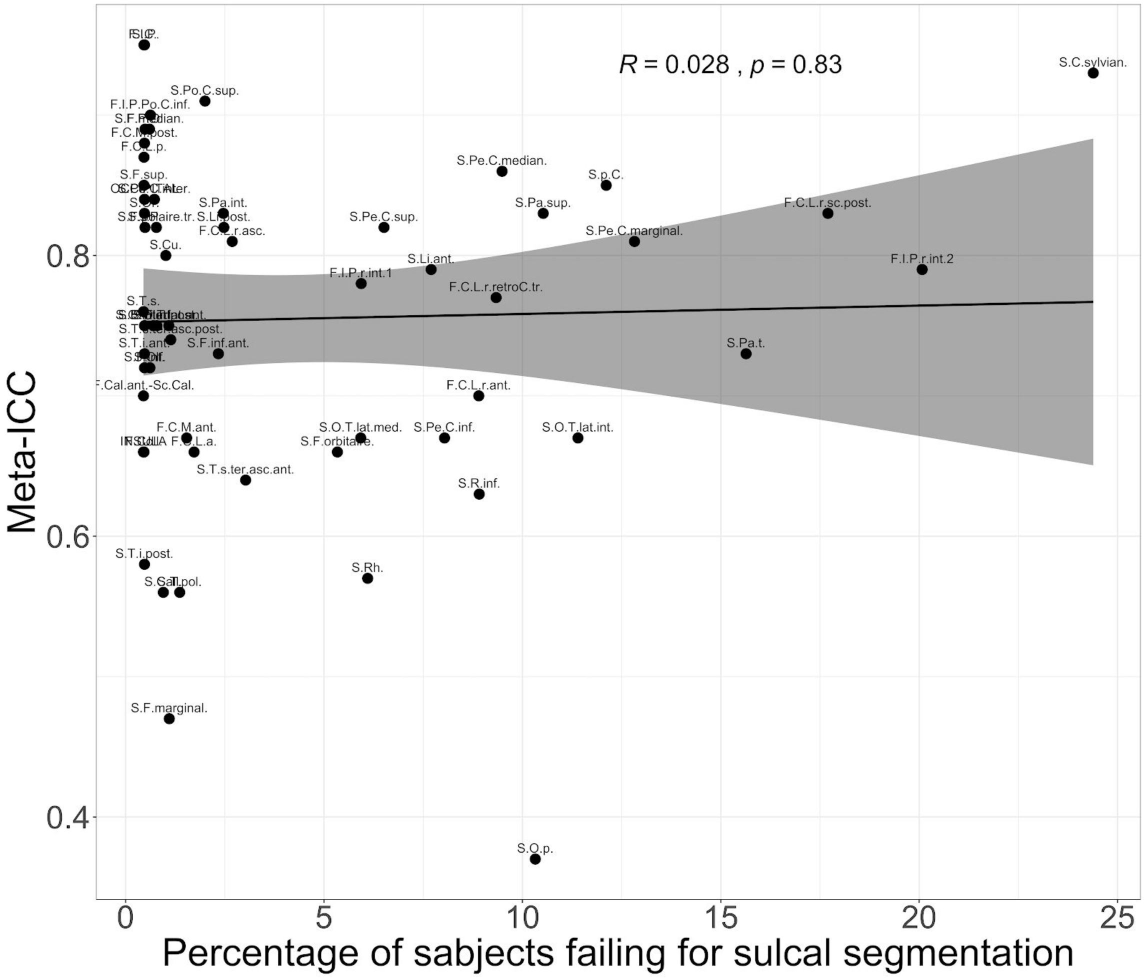

Supplementary ***Figure 31:*** *Correlation between Meta-ICC estimated for sulcal width and the percentage of subjects failing for sulcal segmentation averaged across cohorts (QTIM, HCP, GOBS and UKBB) and hemispheres.*

Bland-Altman plots to visualize the bias in sulcal estimates

For each sulcus the Bland-Altman plots have been generated for the four test-retest cohorts (QTIM, HCP, KKI, OASIS) and for the fours sulcal descriptors (length, mean depth, width and surface area). Such plots are available following this link:
https://drive.google.com/drive/folders/1UrNsBUDXmJpx9-9EDxNqUwtSdrq5_oQK?usp=sharing

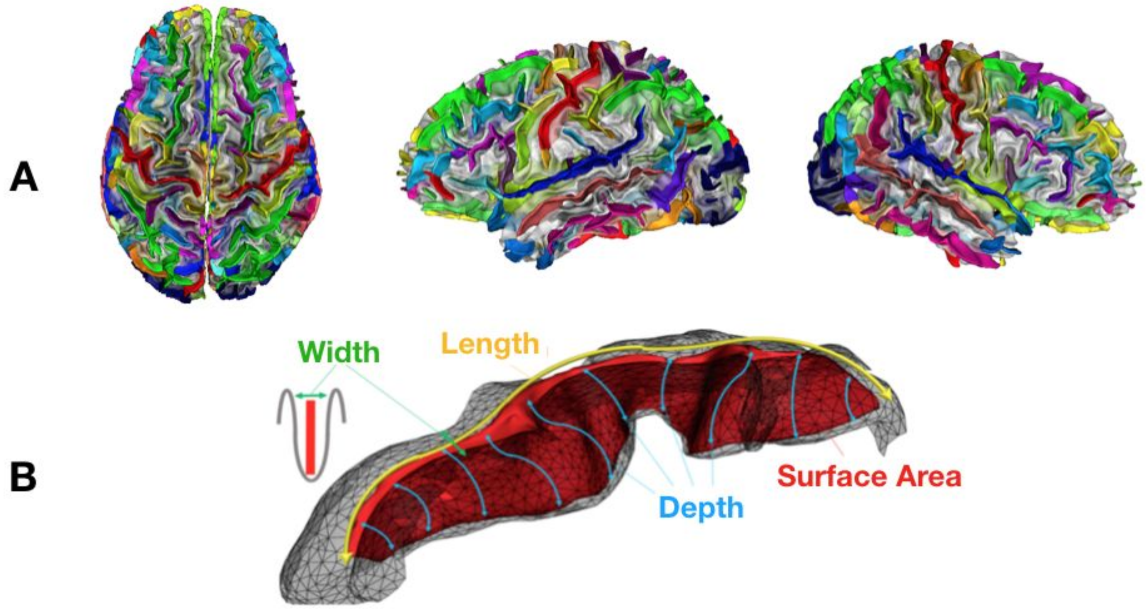

Supplementary Figure 32: A) Surfacic representation of labeled sulci extract from one random subject analyzed here. B) Representation of sulcal measurements (edited from [6]). For a representation of labeled sulci, please refer to the BrainVISA webpage following this link: http://brainvisa.info/web/_static/images/bsa/brainvisa_sulci_atlas_with_table_150dpi-r90.png

CORRELATION BETWEEN SULCAL MEASURES AND ICV

Supplementary Figure 33 shows the Person's correlation between each of the sulcal measures analyzed here and the intracranial cortical volume (ICV) extracted with Freesurfer and used as covariate for estimating the heritability. Length and surface area show the highest (positive) correlation among the descriptors. Sulcal width seems the less correlated measure. Moreover, sulcal width shows weak negative correlation for the precentral sulcus in QTIM, the internal parietal sulcus in HCP and anterior inferior frontal sulcus in GOBS.

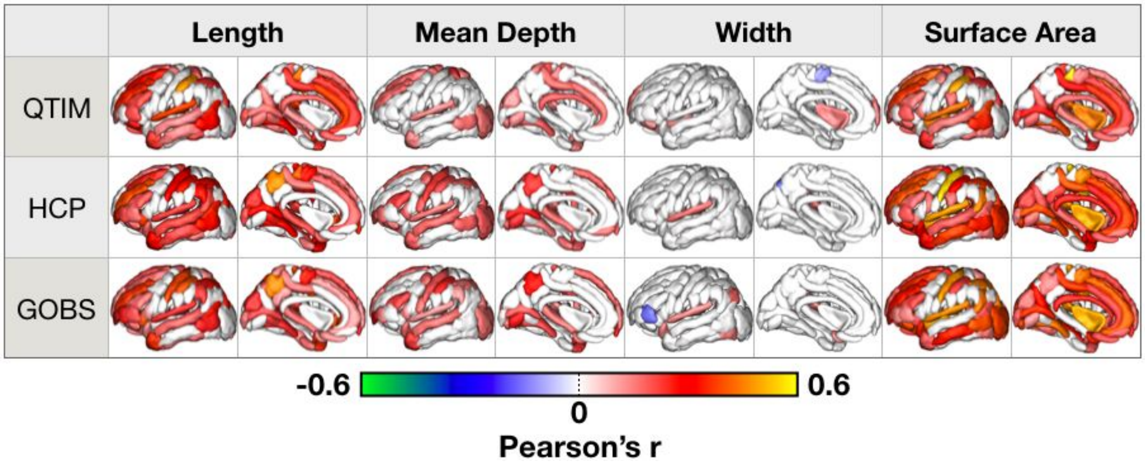

Supplementary Figure 33: *Pearson's correlation between sulca length, mean depth, width and surface area and ICV, computed for QTIM, HCP and GOBS (Significant regions survived Bonferroni correction for multiple comparisons across all bilateral traits and regions (p < 0.05/(61*4)). The average and standard deviation, estimated across sulci, of Person's correlation is tabulated in Supplementary Data*

25-27 for each sulcal descriptor and cohort. Supplementary Data 28 reports the average of the Pearon's correlation between sulcal measurements and ICV.

REFERENCES

1. Shrout, P. E. & Fleiss, J. L. Intraclass correlations: uses in assessing rater reliability. *Psychol. Bull.* **86**, 420–428 (1979).
2. Dubois, J. *et al.* Mapping the early cortical folding process in the preterm newborn brain. *Cereb. Cortex* **18**, 1444–1454 (2008).
3. Desikan, R. S. *et al.* An automated labeling system for subdividing the human cerebral cortex on MRI scans into gyral based regions of interest. *Neuroimage* **31**, 968–980 (2006).
4. Chiarello, C., Vazquez, D., Felton, A. & McDowell, A. Structural asymmetry of the human cerebral cortex: Regional and between-subject variability of surface area, cortical thickness, and local gyrification. *Neuropsychologia* **93**, 365–379 (2016).
5. Koelkebeck, K. *et al.* The contribution of cortical thickness and surface area to gray matter asymmetries in the healthy human brain. *Hum. Brain Mapp.* **35**, 6011–6022 (2014).
6. Pizzagalli, F. *et al.* Genetic analysis of cortical sulci in 1,009 adults. in *2016 IEEE 13th International Symposium on Biomedical Imaging (ISBI)* 833–837 (2016).
