# Supplementary material for: The reliability and heritability of cortical folds and their genetic correlations across hemispheres
Source: Commun Biol. 2020 Sep 15;3:510. doi: 10.1038/s42003-020-01163-1 (PMC7493906; doi:10.1038/s42003-020-01163-1)
Supplement: Supplementary file 2 — Supplementary Data [file 42003_2020_1163_MOESM2_ESM.pdf]

Supplementary Data 1: Sulci nomenclature: BrainVISA sulcal labels are reported along with the anatomical nomenclature and the corresponding brain area.

Supplementary Data 2: ICC for the left hemisphere sulcal descriptors (yellow: ICC > 0.75; orange: ICC > 0.9).

Supplementary Data 3: ICC for the right hemisphere sulcal descriptors (yellow: ICC > 0.75; orange: ICC > 0.9).

Supplementary Data 4: ICC for the bilaterally average sulcal descriptors (yellow: ICC > 0.75; orange: ICC > 0.9).

Supplementary Data 5: Bland-Altman analysis: estimation of bias b (mean difference between test and retest) in % for left, right, and averaged sulcal shape descriptors. Mean (SD) of b across cohorts is reported. b estimates the differences between scan and rescan. While the ICC estimates the relation between within-subject variance and between-subjects variance, b represents a subject-based index. If scan and rescan are perfectly reliable, b should be equal to zero. Here we highlight those cases showing b greater than 0.1 as in 2. Sulcal-based values of b are reported in Supplementary Data_S7-S8 separately for each cohort and sulcal descriptor.

Supplementary Data 6: bias (b) values for the left hemisphere sulcal descriptors (red: abs(b) > 0.1 or NaN; orange: abs(b) > 1).

Supplementary Data 7: bias (b) values for the right hemisphere sulcal descriptors (red: abs(b) > 0.1 or NaN; orange: abs(b) > 1).

Supplementary Data 8: bias (b) values for the bilaterally average sulcal descriptors (red: abs(b) > 0.1 or NaN; orange: abs(b) > 1).

Supplementary Data 9: heritability estimates for left hemisphere sulcal descriptors (yellow: bonferroni corrected for 123*4 comparisons) in QTIM.

Supplementary Data 10: heritability estimates for right hemisphere sulcal descriptors (yellow: bonferroni corrected for 123*4 comparisons) in QTIM.

Supplementary Data 11: heritability estimates for bilaterally average sulcal descriptors (yellow: bonferroni corrected for 61*4 comparisons) in QTIM.

Supplementary Data 12: heritability estimates for left hemisphere sulcal descriptors (yellow: bonferroni corrected for 123*4 comparisons) in HCP.

Supplementary Data 13: heritability estimates for right hemisphere sulcal descriptors (yellow: bonferroni corrected for 123*4 comparisons) in HCP.

Supplementary Data 14: heritability estimates for bilaterally average sulcal descriptors (yellow: bonferroni corrected for 61*4 comparisons) in HCP.

Supplementary Data 15: heritability estimates for left hemisphere sulcal descriptors (yellow: bonferroni corrected for 123*4 comparisons) in GOBS.

Supplementary Data 16: heritability estimates for right hemisphere sulcal descriptors (yellow: bonferroni corrected for 123*4 comparisons) in GOBS.

Supplementary Data 17: heritability estimates for bilaterally average sulcal descriptors (yellow: bonferroni corrected for 61*4 comparisons) in GOBS.

Supplementary Data 18: heritability estimates for bilaterally average sulcal descriptors (yellow: bonferroni corrected for 61*4 comparisons) in UK Biobank.

Supplementary Data 19: Meta-analysis of heritability estimates for bilaterally average sulcal descriptors (yellow: bonferroni corrected for 61*4 comparisons).

Supplementary Data 20: Mega-analysis of heritability estimates for bilaterally average sulcal descriptors (yellow: bonferroni corrected for 61*4 comparisons).

Supplementary Data 21: Bivariate analysis: left-right genetic correlation in QTIM (yellow: Bonferroni corrected; grey: $p < 0.05$).

Supplementary Data 22: Bivariate analysis: left-right genetic correlation in HCP (yellow: Bonferroni corrected; grey: $p < 0.05$).

Supplementary Data 23: Bivariate analysis: left-right genetic correlation in GOBS (yellow: Bonferroni corrected; grey: $p < 0.05$).

Supplementary Data 24: Meta-analysis of left-right genetic correlation (yellow: Bonferroni corrected; grey: $p < 0.05$).

Supplementary Data 25: QTIM; Correlation with ICV (orange: Bonferroni corrected)

Supplementary Data 26: HCP; Correlation with ICV (orange: Bonferroni corrected)

Supplementary Data 27: GOBS; Correlation with ICV (orange: Bonferroni corrected)

Supplementary Data 28: Correlation between intracranial cortical volume (ICV) and sulcal length, mean depth, width and surface area
