# Supplementary material for: The reliability and heritability of cortical folds and their genetic correlations across hemispheres
Source: Commun Biol. 2020 Sep 15;3:510. doi: 10.1038/s42003-020-01163-1 (PMC7493906; doi:10.1038/s42003-020-01163-1)
Supplement: Supplementary file 4 — Reporting Summary [file 42003_2020_1163_MOESM4_ESM.pdf]

nature research

Corresponding author(s): Neda Jahanshad

Last updated by author(s): Oct 30, 2019

Reporting Summary

Nature Research wishes to improve the reproducibility of the work that we publish. This form provides structure for consistency and transparency in reporting. For further information on Nature Research policies, see Authors & Referees and the Editorial Policy Checklist.

Statistics

For all statistical analyses, confirm that the following items are present in the figure legend, table legend, main text, or Methods section.

n/a	Confirmed	
☐	☒	The exact sample size (*n*) for each experimental group/condition, given as a discrete number and unit of measurement
☐	☒	A statement on whether measurements were taken from distinct samples or whether the same sample was measured repeatedly
☐	☒	The statistical test(s) used AND whether they are one- or two-sided<br>*Only common tests should be described solely by name; describe more complex techniques in the Methods section.*
☐	☒	A description of all covariates tested
☐	☒	A description of any assumptions or corrections, such as tests of normality and adjustment for multiple comparisons
☐	☒	A full description of the statistical parameters including central tendency (e.g. means) or other basic estimates (e.g. regression coefficient) AND variation (e.g. standard deviation) or associated estimates of uncertainty (e.g. confidence intervals)
☐	☒	For null hypothesis testing, the test statistic (e.g. *F*, *t*, *r*) with confidence intervals, effect sizes, degrees of freedom and *P* value noted<br>*Give P values as exact values whenever suitable.*
☒	☐	For Bayesian analysis, information on the choice of priors and Markov chain Monte Carlo settings
☒	☐	For hierarchical and complex designs, identification of the appropriate level for tests and full reporting of outcomes
☐	☒	Estimates of effect sizes (e.g. Cohen's *d*, Pearson's *r*), indicating how they were calculated

Our web collection on statistics for biologists contains articles on many of the points above.

Software and code

Policy information about availability of computer code

Data collection	*Provide a description of all commercial, open source and custom code used to collect the data in this study, specifying the version used OR state that no software was used.*
Data analysis	Freesurfer v5.3 and BrainVISA 4.4 were used for MRI data preprocessing. These softwares are publicly available and the preprocessing steps are described in the Method section. R v3.5 scripts were used for statistical analysis and SOLAR-ECLIPSE imaging genetics tools (http://www.nitrc.org/projects/se_linux) was used for heritability and genetic correlation estimation.

For manuscripts utilizing custom algorithms or software that are central to the research but not yet described in published literature, software must be made available to editors/reviewers. We strongly encourage code deposition in a community repository (e.g. GitHub). See the Nature Research guidelines for submitting code & software for further information.

Data

Policy information about availability of data

All manuscripts must include a data availability statement. This statement should provide the following information, where applicable:

- Accession codes, unique identifiers, or web links for publicly available datasets
- A list of figures that have associated raw data
- A description of any restrictions on data availability

- Accession codes, unique identifiers, or web links for publicly available datasets

OASIS: The OASIS data are distributed the greater scientific community under the Creative Commons Attribution 4.0 license. All data is available via www.oasis-brains.org 1 .
KKI (Kennedy Krieger Institute - Multimodal MRI Reproducibility Resource): Open access: https://www.nitrc.org/projects/multimodal/ 2 .

- A list of figures that have associated raw data

Data for all figures, including for example heritability estimates and standard errors, are available in the supplementary tables.

- A description of any restrictions on data availability

QTIM: Data from the QTIM cohort used in this manuscript can be applied for by contacting Dr. Margaret Wright (margie.wright@uq.edu.au). Access to data by qualified investigators are subject to scientific and ethical review. Summary results from cohort QTIM are available as part of the supplementary data 3 .
HCP: Family status and other potentially sensitive information are part of the Restricted Data that is available only to qualified investigators after signing the Restricted Data Use Terms. Open access data (all imaging data and most of the behavioral data) is available to those who register and agree to the Open Access Data Use Terms.
Restricted data elements that could be potentially used to identify subjects include family structure (twin or non-twin status and number of siblings); birth order; age by year; handedness; ethnicity and race; body height, weight, and BMI; and a number of other categories. Each qualified investigator wanting to use restricted data must apply for access and agree to the Restricted Data Use Terms (https://humanconnectome.org/study/hcp-young-adult/data-use-terms) 4 .
GOBS: Data from the GOBS cohort used in this manuscript can be applied for by contacting Prof. David Glahn (david.glahn@childrens.harvard.edu) or Prof. John Blangero (John.Blangero@utrgv.edu). Access to data by qualified investigators are subject to scientific and ethical review and must comply with the European Union General Data Protection Regulations (GDPR)/all relevant guidelines. The completion of a material transfer agreement (MTA) signed by an institutional official will be required. Summary results from cohort GOBS are available as part of the supplementary data .
UKBB: Access to data from the UK Biobank can be obtained by approved scientists through application with UK Biobank (www.ukbiobank.ac.uk/researchers) 6 .

REFERENCES
1. Marcus, D. S. et al. Open Access Series of Imaging Studies (OASIS): Cross-sectional MRI Data in Young, Middle Aged, Nondemented, and Demented Older Adults. Journal of Cognitive Neuroscience 19, 1498–1507 (2007).
2. Landman, B. A. et al. Multi-parametric neuroimaging reproducibility: a 3-T resource study. Neuroimage 54, 2854–2866 (2011).
3. de Zubicaray, G. I. et al. Meeting the Challenges of Neuroimaging Genetics. Brain Imaging Behav. 2, 258–263 (2008).
4. Van Essen, D. C. et al. The Human Connectome Project: a data acquisition perspective. Neuroimage 62, 2222–2231 (2012).
5. Olvera, R. L. et al. Common genetic influences on depression, alcohol, and substance use disorders in Mexican-American families. Am. J. Med. Genet. B Neuropsychiatr. Genet. 156B, 561–568 (2011).
6. Sudlow, C. et al. UK biobank: an open access resource for identifying the causes of a wide range of complex diseases of middle and old age. PLoS Med. 12, e1001779 (2015).

Field-specific reporting

Please select the one below that is the best fit for your research. If you are not sure, read the appropriate sections before making your selection.

☒ Life sciences ☐ Behavioural & social sciences ☐ Ecological, evolutionary & environmental sciences

For a reference copy of the document with all sections, see nature.com/documents/nr-reporting-summary-flat.pdf

Life sciences study design

All studies must disclose on these points even when the disclosure is negative.

Sample size	We performed an extensive reliability (N=110) and heritability (N=13,113) analysis. We analyzed heritability in four independent cohorts, three with a family based design and one using single-nucleotide polymorphism (SNP) based heritability estimates. The cohorts included two twin-based samples (QTIM and HCP), one cohort of extended pedigrees (the Genetics of Brain Structure and Function; GOBS), and another of over 9,000 largely unrelated individuals (the UK Biobank).
Data exclusions	n/a
Replication	We estimated the heritability of sulcal traits in 4 independent cohorts which show high degree of reproducibility as reported in the manuscript.
Randomization	Reliability was estimated in repeated scan (delay < 90 days) of same subjects to estimate the errors in sulcal traits measurements. For heritability estimations we included as convariates: intracranial volume, sex, age, age2, age × sex interaction, age2 × sex interaction) to calculate heritability and its significance (p-value) for accounting for a component of each trait's variance within this population.
Blinding	Blinding was not necessary; we used statistical methods, not related to the operator.

Reporting for specific materials, systems and methods

We require information from authors about some types of materials, experimental systems and methods used in many studies. Here, indicate whether each material, system or method listed is relevant to your study. If you are not sure if a list item applies to your research, read the appropriate section before selecting a response.

Materials & experimental systems

n/a	Involved in the study
☒	☐ Antibodies
☒	☐ Eukaryotic cell lines
☒	☐ Palaeontology
☒	☐ Animals and other organisms
☒	☐ Human research participants
☐	☒ Clinical data

Methods

n/a	Involved in the study
☒	☐ ChIP-seq
☒	☐ Flow cytometry
☐	☒ MRI-based neuroimaging

Clinical data

Policy information about clinical studies

All manuscripts should comply with the ICMJE guidelines for publication of clinical research and a completed CONSORT checklist must be included with all submissions.

Clinical trial registration: *Provide the trial registration number from ClinicalTrials.gov or an equivalent agency.*

Study protocol: *Note where the full trial protocol can be accessed OR if not available, explain why.*

Data collection: *Describe the settings and locales of data collection, noting the time periods of recruitment and data collection.*

Outcomes: *Describe how you pre-defined primary and secondary outcome measures and how you assessed these measures.*

Magnetic resonance imaging

Experimental design

Design type: *Indicate task or resting state; event-related or block design.*

Design specifications: *Specify the number of blocks, trials or experimental units per session and/or subject, and specify the length of each trial or block (if trials are blocked) and interval between trials.*

Behavioral performance measures: *State number and/or type of variables recorded (e.g. correct button press, response time) and what statistics were used to establish that the subjects were performing the task as expected (e.g. mean, range, and/or standard deviation across subjects).*

Acquisition

Imaging type(s): Structural MRI (T1w)

Field strength: OASIS:1.5T, KKI: 3T, HCP:3T, QTIM:4T, UKBB: 3T

Sequence & imaging parameters:

QTIM: T1-weighted images were acquired on a 4 T Bruker Medspec scanner with an inversion recovery rapid gradient echo sequence. Acquisition parameters were: inversion/repetition/echo time (TI/TR/TE) = 700/1500/3.35 ms; flip angle = 8 degrees; with an acquisition matrix of 256 × 256; voxel size= 0.94 x 0.90 x 0.94 mm3.

HCP: T1-weighted images were acquired using a 3T Siemens scanner. MRI parameters: (TI/TR/TE) = 1000/2400/2.14 ms; flip angle = 8 degrees; voxel size = 0.7 mm isotropic, acquisition matrix = 224 × 224. The subset of test-retest scans includes all right-handed subjects.

GOBS: Imaging data were acquired at the UTHSCSA Research Imaging Center on a Siemens 3 T Trio scanner (Siemens, Erlangen, Germany). Isotropic (800 μm) 3D Turbo-flash T1-weighted images were acquired with the following parameters: TE/TR/TI = 3.04/2100/785 ms, flip angle = 13 degrees. Seven images were acquired consecutively using this protocol for each subject and the images were then co-registered and averaged to increase the signal-to-noise ratio and reduce motion artifacts 84.

UK Biobank: T1-weidghted acquisitions were conducted on a Siemens Skyra 3 tesla scanner. Voxel matrix: 1.0x1.0x1.0 mm - acquisition matrix: 208x256x256. 3D MP-RAGE, TI/TR=880/2000 ms, sagittal orientation, in-plane acceleration factor=2. Raw MRI data were processed using the ENIGMA FreeSurfer and sulcal analysis protocols. Following processing, all images were visually inspected for FreeSurfer quality control of grey/white matter classifications. The central sulcus segmented and labeled by BrainVISA was also visually controlled for labeling quality for all subjects.

KKI: All data were acquired using a 3 T MRI scanner (Achieva, Philips Healthcare, Best, The Netherlands) with body coil excitation and an eight-channel phased array SENSitivity Encoding (SENSE) head-coil for reception. All scans were completed during a 2-week interval. The resulting dataset consisted of 42 “1-h” sessions of 21 individuals. MP-RAGE T1-weighted scans were acquired with a 3D inversion recovery sequence: (TR/TE/TI = 6.7/3.1/842 ms) with a 1.0 × 1.0 × 1.2 mm3 resolution over a field of view of 240 × 204 × 256 mm acquired in the sagittal plane. The SENSE acceleration factor was 2 in the right–left direction. Multi-shot fast gradient echo (TFE factor = 240) was used with a 3-s shot interval and

the turbo direction being in the slice direction (right–left). The flip angle was 8 degrees. No fat saturation was employed 87, https://www.nitrc.org/projects/multimodal/.

OASIS: MPRAGE T1-weighted scans were acquired on a 1.5-T Vision scanner (Siemens, Erlangen, Germany): (TR/TE/TI = 9.7/4.0/20 ms) with an in-plane resolution of 1.0 × 1.0 × mm2 resolution over a FOV of 256 × 256 mm acquired in the sagittal plane. Thickness/gap= 1.25/0 mm; flip angle = 10 degrees. (https://www.oasis-brains.org/) 88.

Area of acquisition: Whole brain

Diffusion MRI: ☐ Used ☒ Not used

Preprocessing

Preprocessing software: Freesurfer v5.3 and BrainVISA 4.4 were used for MRI data preprocessing. These softwares are publicly available and the preprocessing steps are described in the Method section. R v3.5 scripts were used for statistical analysis and SOLAR-ECLIPSE imaging genetics tools (http://www.nitrc.org/projects/se_linux) was used for heritability and genetic correlation estimation.

Normalization: *If data were normalized/standardized, describe the approach(es): specify linear or non-linear and define image types used for transformation OR indicate that data were not normalized and explain rationale for lack of normalization.*

Normalization template: *Describe the template used for normalization/transformation, specifying subject space or group standardized space (e.g. original Talairach, MNI305, ICBM152) OR indicate that the data were not normalized.*

Noise and artifact removal: *Describe your procedure(s) for artifact and structured noise removal, specifying motion parameters, tissue signals and physiological signals (heart rate, respiration).*

Volume censoring: *Define your software and/or method and criteria for volume censoring, and state the extent of such censoring.*

Statistical modeling & inference

Model type and settings: Reliability was estimated using the intra-class correlation coefficient (ICC). Heritability was estimated using a univariate model for each trait estimation and a bi-variate model for left-right hemisphere genetic correlation.

Effect(s) tested: *Define precise effect in terms of the task or stimulus conditions instead of psychological concepts and indicate whether ANOVA or factorial designs were used.*

Specify type of analysis: ☐ Whole brain ☐ ROI-based ☒ Both

Anatomical location(s): Probabilisti atlas implemente in BrainVISA was used to automatically segment and label 123 sulci across the whole brain.

Statistic type for inference (See Eklund et al. 2016): *Specify voxel-wise or cluster-wise and report all relevant parameters for cluster-wise methods.*

Correction: Bonferroni correction was used for all the statistical analyses. Detailed information are reported in the manuscript when the results are presented.

Models & analysis

n/a	Involved in the study
☒	☐ Functional and/or effective connectivity
☒	☐ Graph analysis
☒	☐ Multivariate modeling or predictive analysis
