# Supplementary material for: The reliability and heritability of cortical folds and their genetic correlations across hemispheres
Source: Commun Biol. 2020 Sep 15;3:510. doi: 10.1038/s42003-020-01163-1 (PMC7493906; doi:10.1038/s42003-020-01163-1)
Supplement: Supplementary file 5 — Description of Additional Supplementary Files [file 42003_2020_1163_MOESM5_ESM.pdf]

Reviewers' comments:

Reviewer #1 (Remarks to the Author):

Pizzagalli and colleagues present an in-depth look at the reliability and heritability of four different sulcal measures across 61 unique sulci: length, depth, width, and surface area. There have been many imaging papers using such sulcal measures and their relationship with human behavior and cognition, as well as extensive applications to disease. Thus, the authors propose highly relevant analyses to better understand the genetic architecture of this strongly neurodevelopmentally-mediated brain metric. The methodology underlying the authors' hypotheses is also very strong. I am enthusiastic about these results being published in Communications Biology - however, I have some questions and suggestions that can help improve the paper.

Intro

1)Paragraph 2 of intro: We have a lot of evidence that other parts of the brain apart from the cortex (white matter, cerebellum, subcortical structures) play a role in human cognition and behavior, so it is a bit misleading to highlight only the brain's cortical gray matter's involvement in this. This sentence could be reframed to state that cortical gray matter plays an important role in cognitive function and behavior. Thickness and area, which are highlighted by the authors a few sentences after, are also not simply indices of cortical gray matter.

Methods

2) The authors state that cortical thickness and surface area are "largely genetically correlated" which is not what is often described when comparing these two measures (Strike et al, Cerebral Cortex 2018; Grasby et al., biorxiv 2019 [https://www.biorxiv.org/content/10.1101/399402v3])

3) The description of what cortical thickness represents could be improved from simply "determined by the horizontal layers in the cortical mantle", I would mention number of neurons as well

4) Please define CSF in the neuroimaging section of the methods

5) Having a different sample per sulcus makes it hard to harmonize the interpretation of results. It would be helpful if authors included the number of subjects dropped per sulcus, and to provide a sense of how many subjects failed for 2 or more sulci.

6) It would be helpful to see a figure that labels the 61 different sulci in the study on brain surfaces. It would also be great to add a schematic of the four different sulcal measures defined.

7) Many sulci can be hard to measure across individuals or may not even be present. Was visual quality control of these sulci done at all? For instance, this could be feasible to do for the test-retest reliability cohorts with fewer individuals. The authors discuss an automated check for outliers, but certain hard-to-measure sulci may be giving consistently biased estimates or highly heterogeneous estimates where simply dropping an outlier doesn't mean the retained subjects have anatomically accurate labels of these sulci. Which sulcus had the highest number of dropped subjects? Does it correspond to those with the lowest ICC as well?

Results

8) Bland-Altman plots to visualize the bias in sulcal estimates, rather than in table format, would be helpful for readers.

9) The authors note that sulcal length heritability was more often significant when NOT adjusting for ICV. Can the authors provide correlations between ICV and sulcal estimates?

10) As the authors note, and as expected, there are significant correlations between heritability and reliability. Following this point, it may be worthwhile to covary for ICC in the heritability analyses.

11) The authors include many measures of ICC, both within and between-subjects, but these reliability measures are highly dependent on the gold standard or atlas used: it is highly recommended to show and discuss the atlas upon which the sulcal estimates were derived.

Discussion
12) The authors describe that "earlier forming sulci" are more heritable, while still acknowledging that heritability estimates are largely impacted by reliability. To add to this interpretation, one could argue that ICC seems to be highest for sulci that are considered landmarks in the brain and are more invariant between individuals. I would suggest adding to this interpretation that it is also possible that these sulci are larger and easier to define (such as the central sulcus, lateral fissure), likely have lower ICC, and thus also higher heritability.

13) The authors conclude that there is very little lateralization of heritability estimates across hemispheres - however, some of the samples included seemed to include only right-handed individuals. This may have impacted results - for instance, QTIM included only right-handed individuals and the authors noted that this sample had higher heritability for right hemi sulci. Thus it should be added somewhere to the discussion or limitations section that including more individuals to approximate what is seen in true populations in terms of handedness (e.g. left-handed individuals, ambidexterity) might change these results.

Reviewer #2 (Remarks to the Author):

This study examined the reliability and heritability of sulcal folding features including length, depth, width and surface area that were extracted from large-scale in vivo brain MRI using BrainVisa. The study is methodologically well-designed, and the manuscript is well-written in general. However, I have two major comments.

First, although various results were generated and reported, it's little hard to capture the difference and originality of this study when compared to prior studies. In introduction, some description is too general. It needs to be more specific, and more relevant studies have to be introduced and reviewed. Is the originality of this study using the large multi-center dataset? first analysis of the reliability and heritability of "sulcal morphometric features"? more sophisticated genetic correlation analysis? or lateralization of genetic effects on the sulcal folding? It should be clearer by comparing with more prior studies.

Another comment is about interpretation of the results. What's the interpretation for different results of the different sulcal features? What is the different biological meaning between sulcal length, depth, width and surface area in these results? In discussion, many descriptions are repetition of the results. Instead, the discussion should be more focused on the interpretation of the results and original findings of this study should be clarified and supported by better discussion.

Reviewer #3 (Remarks to the Author):

The authors have assessed the reliability of cortical sulci features extraction using the Brainvisa suite. They first demonstrated a reasonable reliability of extraction for these sulcal metrics. Then they pursued by estimating the heritability of these features. They present their results by mapping their ICC and h2 on interesting sulci brain map, showing high regional variability in both reliability and heritability estimates.
The results obtained go further here than a previous publication (1), which had only reported the heritability on part of the sulcal metrics in the UK Biobank. However, the authors failed to compare their result with this publication.

1. eQTL of KCNK2 regionally influences the brain sulcal widening: evidence from 15,597 UK Biobank participants with neuroimaging data

They also did not emphasize the large difference between SNP and pedigree base heritability.
The authors only wrote "Overall, the pattern of heritability estimates were largely coherent between the family based and large-scale population studies."

For example on Figure 2, the width of the central sulcus seems to have a Family based h2 of 0.7, while SNP based h2 is 0.2-0.3. These important differences will make it difficult for future works to settle on which level of heritability to cite for these sulcal metrics.

What could explain such large difference in heritability estimate between UK Biobank SNP-based estimate and the Family-based estimate? Methods? Age difference between cohorts? Less environmental influence in some cohorts? Measurement errors?
The authors write "SNP-based heritability estimated in the UK Biobank shows a similar pattern (Figure S20) across the brain, but with lower h2 values compared to the family based cohorts. This might be due to the "missing heritability" effect arising in the SNP-based heritability estimation 71."
(2) and (3) below seem to contradict this hypothesis by using both methods in HCP and obtaining similar estimates for both methods. Please comment.

2. Genomic kinship construction to enhance genetic analyses in the human connectome project data
3. Heritability of surface area and cortical thickness: a comparison between the Human Connectome Project and the UK Biobank dataset

The authors also emphasize an interesting result, showing a positive correlation between the reliability of extraction and the heritability. They appropriately discuss this important confirmatory result: "Indeed, measurement error could lead to ceiling effect on heritability estimates, and genetic correlations. Highly heritable traits can be detected only if the traits are robustly measured 50 and low reliability could lead to an underestimation of the true heritability 51."

I agree with the authors that it is necessary to demonstrate both the reliability and heritability of a trait before deeming it worth to analyze in a genome-wide association study in ENIGMA like consortia. Overall, the authors demonstrated the reliability and heritability for most sulci of the metrics under consideration. Thus, this work lays the foundation to promote such large-scale meta-analysis of these sulcal metrics.

Last, the authors pinpoint a strong genetic correlation between contralateral measures for a subset of sulci, while also identifying a subset with unique genetic influence in one hemisphere. The later group may reveal insights into the brain lateralization and thus promotes further genetic study of these regions analyzing left and right hemispheres separately.

Why Supplementary Tables numbering starts at S3, and includes an SA?

COMMSBIO-19-1499-B

Notes:

The submitted manuscript has been edited in the Abstract, from:

The structure of the brain's cortical folds varies considerably in human populations. Specific patterns of cortical variation arise with development and aging, and cortical traits are partially influenced by genetic factors. The degree to which genetic factors affect cortical folding patterning remains unknown, yet may be estimated with large-scale *in-vivo* brain MRI. Using multiple MRI datasets from around the world, we estimated the reliability and heritability of sulcal morphometric characteristics including length, depth, width, and surface area, for 61 sulci per hemisphere of the human brain. Reliability was assessed across four distinct test-retest datasets. We meta-analyzed the heritability across three independent family-based cohorts (N > 3,000), and one cohort of largely unrelated individuals (N~9,000) to examine the robustness of our findings. Reliability was high (interquartile range for ICC: 0.65-0.85) for sulcal metrics. Most sulcal measures were moderately to highly heritable (0.3-0.7). These genetic influences vary regionally, with the earlier forming sulci having higher heritability estimates. The central sulcus, the subcallosal and the collateral fissure were the most highly heritable regions. For some frontal and temporal sulci, left and right genetic influences did not completely overlap, suggesting some lateralization of genetic effects on the cortex.

To:

Cortical folds help drive the parcellation of the human cortex into functionally specific regions. Variations in the length, depth, width, and surface area of these sulcal landmarks have been associated with disease, and may be genetically mediated. Before estimating the heritability of sulcal variation, the extent to which these metrics can be reliably extracted from *in-vivo* MRI must be established. Using four independent test-retest datasets, we found high reliability across the brain (intraclass correlation interquartile range: 0.65-0.85). Heritability estimates were derived for three family-based cohorts using variance components analysis and pooled (total N > 3000); the overall sulcal

heritability pattern was correlated to that derived for a large population cohort (N > 9000) calculated using genomic complex trait analysis. Overall, sulcal width was the most heritable metric, and earlier forming sulci showed higher heritability. The inter-hemispheric genetic correlations were high, yet select sulci showed incomplete pleiotropy, suggesting hemisphere-specific genetic influences.

in the Results section, we changed the following text, from:

[...] the GOBS cohort shows lower heritability, (h^2 = 0.3±0.1), compared to QTIM (h^2 = 0.4±0.1) and HCP (h^2 = 0.4±0.1), as might be expected for an extended pedigree design when compared to twin designs (ref. 49).

To

[...] the GOBS cohort shows lower heritability, (h2 = 0.3±0.1), compared to QTIM (h2 = 0.4±0.1) and HCP (h2 = 0.4±0.1); GOBS is a cohort with an extended pedigree design and a wide age range (18-85 years of age), while both HCP and QTIM are twin based cohorts of young adults aged 25-35 years and 20-30 years, respectively.

and in the Acknowledgments section, we changed the text from:

ACKNOWLEDGMENTS. This research was funded in part by NIH ENIGMA Center grant U54 EB020403, supported by the Big Data to Knowledge (BD2K) Centers of Excellence program funded by a cross-NIH. QTIM was supported by NIH R01 HD050735, and the NHMRC 486682, Australia; GOBS: Financial support for this study was provided by the National Institute of Mental Health grants MH078143 (PI: DC Glahn), MH078111 (PI: J Blangero), and MH083824 (PI: DC Glahn & J Blangero); HCP data were provided [in part] by the Human Connectome Project, WU-Minn Consortium (Principal Investigators: David Van Essen and Kamil Ugurbil; 1U54MH091657) funded by the 16 NIH Institutes and Centers that support the NIH Blueprint for Neuroscience Research; and by the McDonnell Center for Systems Neuroscience at Washington University; UK Biobank: This research has been conducted using the UK Biobank Resource under Application Number '11559'; BrainVISA's Morphologist software development has received funding from the European Union's Horizon 2020 Framework Programme for Research and Innovation under Grant Agreement No 720270 & 785907 (Human Brain ProjectSGA1 & SGA2), and by the FRM DIC20161236445. OASIS: Cross-Sectional: Principal Investigators: D. Marcus, R, Buckner, J, Csernansky J. Morris; P50 AG05681, P01 AG03991, P01 AG026276, R01 AG021910, P20 MH071616, U24 RR021382. KKI was supported by NIH grants NCRR P41RR015241 (Peter C.M. Zijl), 1R01NS056307 (Jerry Prince), 1R21NS064534-01A109 (Bennett A. Landman/Jerry L. Prince), 1R03EB012461-01 (Bennett A. Landman).

To:

ACKNOWLEDGMENTS. This research was funded in part by NIH ENIGMA Center grant U54 EB020403, supported by the Big Data to Knowledge (BD2K) Centers of Excellence program funded by a cross-NIH initiative. Additional grant support was provided by: R01 AG059874, R01 MH117601, R01 MH121246, and P41 EB015922. QTIM was supported by NIH R01 HD050735, and the NHMRC 486682, Australia; GOBS: Financial support for this study was provided by the National Institute of Mental Health grants MH078143 (PI: DC Glahn), MH078111 (PI: J Blangero), and MH083824 (PI: DC Glahn & J Blangero); HCP data were provided [in part] by the Human Connectome Project, WU-Minn Consortium (Principal Investigators: David Van Essen and Kamil Ugurbil; 1U54MH091657) funded by the 16 NIH Institutes and Centers that support the NIH Blueprint for Neuroscience Research; and by the McDonnell Center for Systems Neuroscience at Washington University; UK Biobank: This research was conducted using the UK Biobank Resource under Application Number '11559'; BrainVISA's Morphologist software development received funding from the European Union's Horizon 2020 Framework Programme for Research and Innovation under Grant Agreement No 720270 & 785907 (Human Brain ProjectSGA1 & SGA2), and by the FRM DIC20161236445. OASIS: Cross-Sectional: Principal Investigators: D. Marcus, R. Buckner, J. Csernansky J. Morris; P50 AG05681, P01 AG03991, P01 AG026276, R01 AG021910, P20 MH071616, U24 RR021382. KKI was supported by NIH grants NCRR P41 RR015241 (Peter C.M. van Zijl), 1R01NS056307 (Jerry Prince), 1R21NS064534-01A109 (Bennett A. Landman/Jerry L. Prince), 1R03EB012461-01

(Bennett A. Landman). Neda Jahanshad and Paul Thompson are MPIs of a research project grant from Biogen, Inc. (PO 969323).
We thank Anderson M. Winkler for comments and corrections on our initial biorxiv preprint, and Alessandra Griffa for helping to map the FreeSurfer results.

Reviewers' comments:

Reviewer #1 (Remarks to the Author):

Pizzagalli and colleagues present an in-depth look at the reliability and heritability of four different sulcal measures across 61 unique sulci: length, depth, width, and surface area. There have been many imaging papers using such sulcal measures and their relationship with human behavior and cognition, as well as extensive applications to disease. Thus, the authors propose highly relevant analyses to better understand the genetic architecture of this strongly neurodevelopmentally-mediated brain metric. The methodology underlying the authors' hypotheses is also very strong. I am enthusiastic about these results being published in Communications Biology - however, I have some questions and suggestions that can help improve the paper.

The authors thank the reviewer for the positive comments.

Intro

1) *Paragraph 2 of intro: We have a lot of evidence that other parts of the brain apart from the cortex (white matter, cerebellum, subcortical structures) play a role in human cognition and behavior, so it is a bit misleading to highlight only the brain's cortical gray matter's involvement in this. This sentence could be reframed to state that cortical gray matter plays an important role in cognitive function and behavior. Thickness and area, which are highlighted by the authors a few sentences after, are also not simply indices of cortical gray matter.*

We agree with the reviewer and we have rephrased the paragraph as follows:

Enriched in neuronal cell bodies, the cortical gray matter plays an important role in human cognitive functions and behavior, including sensory perception and motor control [1]. Macroscale anatomical features of the human cortex can be reliably extracted from structural MRI scans, and among the most common are regional thickness and surface area measures. These MRI-based features show robust alterations in several neurological, neurodevelopmental, and psychiatric disorders [2], influenced by both environmental and genetic variation [3–5].

Methods

2) The authors state that cortical thickness and surface area are "largely genetically correlated" which is not what is often described when comparing these two measures (Strike et al, Cerebral Cortex 2018; Grasby et al., biorxiv 2019 [https://www.biorxiv.org/content/10.1101/399402v3])

We thank the reviewer for pointing out this typo. We edited that sentence as follows:

Cortical thickness and surface area have both been found to be moderately to highly heritable, yet with largely independent and even negatively correlated genetic influences [3,6,7].

3) The description of what cortical thickness represents could be improved from simply "determined by the horizontal layers in the cortical mantle", I would mention number of neurons as well

We have integrated this suggestion as follows:

In particular, cortical thickness represents the laminar organization of the cerebral cortex, which contains about 14 billion neurons [8]. Each of the layers forming the cortex [9] has a different cellular organization, mostly distinguished on the basis of pyramidal cells in the various laminae [9]. Surface area may reflect the number of radial columns perpendicular to the pial surface[10] and sulcal morphometry may additionally relate to the microstructure of the neuronal sheets and to the local axonal connectivity within a cortical region, which may influence the degree of folding[11].

4) Please define CSF in the neuroimaging section of the methods

CSF is now defined as:

The enclosed cerebrospinal fluid (CSF) volume divided by the sulcal [...]

5) Having a different sample per sulcus makes it hard to harmonize the interpretation of results. It would be helpful if authors included the number of subjects dropped per sulcus, and to provide a sense of how many subjects failed for 2 or more sulci.

We thank the reviewer for this highly relevant comment.

Sulcal segmentation and labelling was performed by importing Freesurfer <u>quality controlled</u> data into the BrainVISA pipeline. As reported in the Methods section:

[...] However, to improve sulcal extraction and build on current protocols used by hundreds of collaborators within ENIGMA, quality controlled FreeSurfer outputs (orig.mgz, ribbon.mgz, and talairach.auto) were directly imported into the pipeline to avoid re-computing several steps, including intensity inhomogeneity correction and gray/white matter classification [...]

We then filtered the sulcal measures by statistically controlling for outliers, as reported in the main text:

To further quality control the extracted sulcal measures and identify subjects whose sulci were not optimally identified, we consider as outliers those subjects showing abnormal values for at least one of the descriptors for each sulcus. For a given sulcus, the z-score across subjects within a cohort is

computed for each descriptor. The set of subjects showing an absolute z-score greater than 2.5 for one or more descriptors were discarded from further analysis [12]. Therefore, if the length of the central sulcus for a given subject was an outlier but width and depth were not, that subject's central sulcus was removed from further evaluation; this ensured that the same set of subjects were used for all analyses across descriptors. This led to discarding ~ 3% of subjects for each sulcus.
and, as reported previously, this method well detects outliers [13].

The sulcal recognition rate of the method used has been reported equal to 85.78% and 86.52% for the left and the right hemispheres, respectively [14]. As the reviewer suggested, we have estimated how many subjects failed for 2 or more sulci, separately for QTIM, HCP, GOBS and UKBB. If BrainVISA fails to identify a sulcus, no values are output for the sulcus, and we consider them "missing". On average, without considering the z-score filtering, four sulci are missing for the majority of subjects. QTIM had a higher percent of sulcal segmentation errors (missing sulci) than either HCP,GOBS or UKBB, possibly due to the fact that it was collected at 4 tesla. This is consistent with previous work showing lower reliability for Freesurfer cortical measures in QTIM than HCP[6]. A detailed description of the quality control findings are presented in the Supplementary Figures S23-S30.

Here the details:

For QTIM, we analyzed data from 1,009 subjects, none of which failed entirely (no BrainVISA output). On average 4 sulci (st dev. 2) failed for each subject in QTIM, (**Figure S23**). **Figure S24** reports the number of subjects failing (in percentage of the total population) for left and right hemispheres.

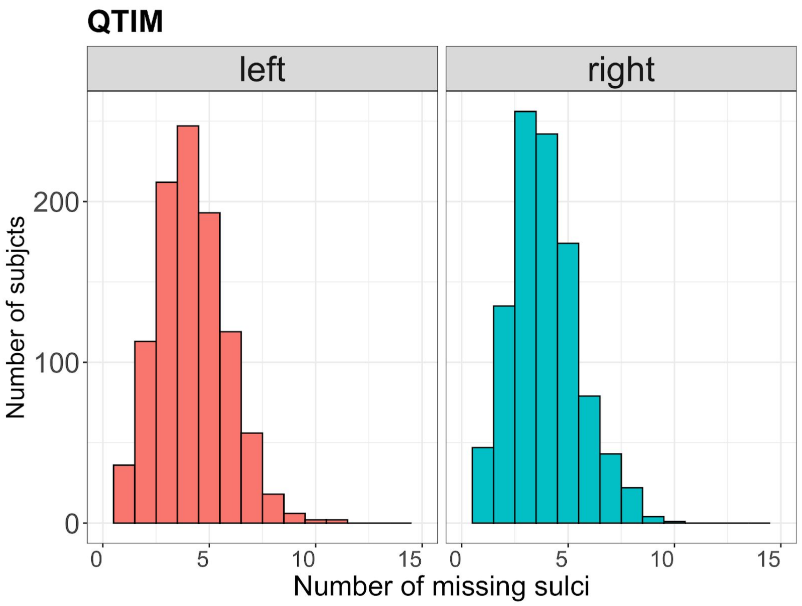

Figure S23: Histogram of number of subjects counted for the missing left and right sulci, in QTIM. The missing sulci are removed before outlier detection.

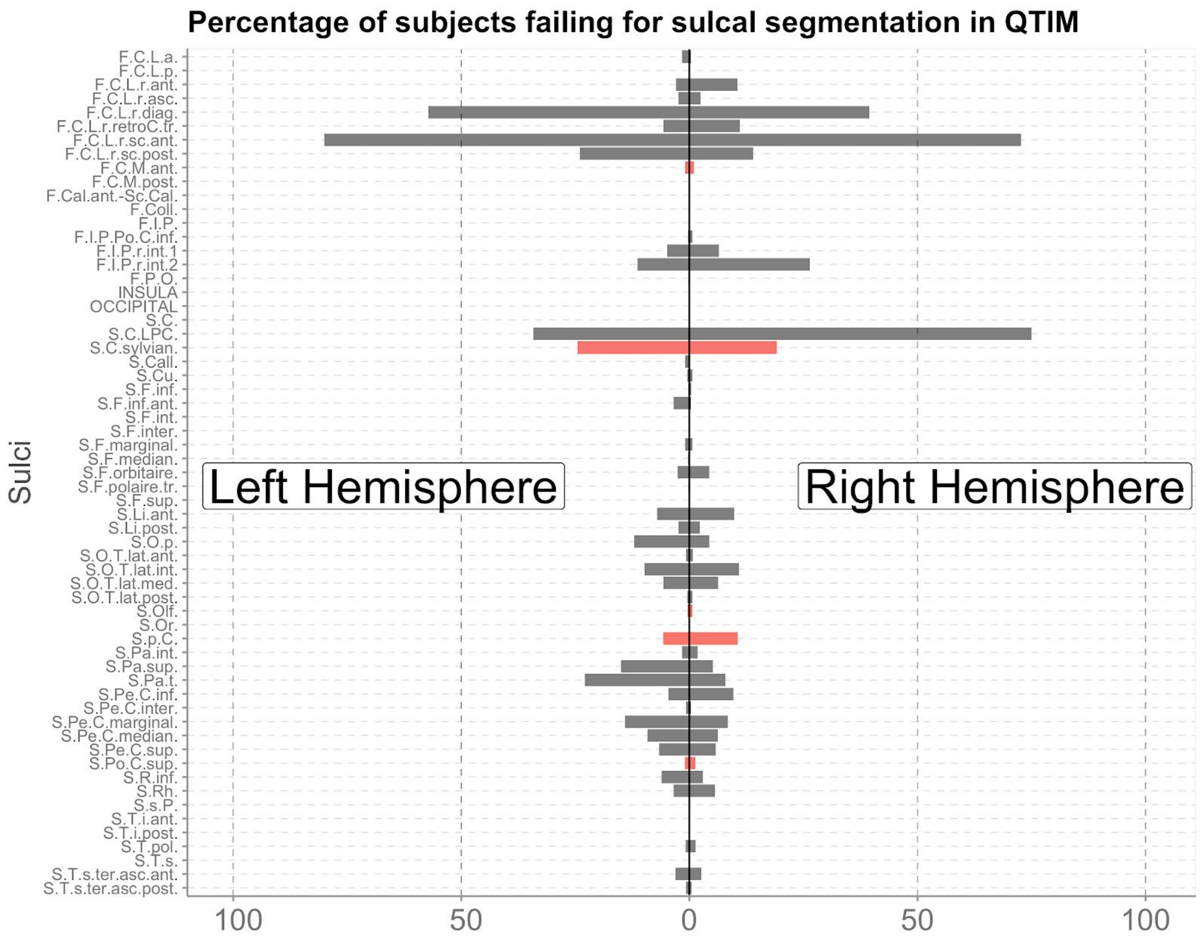

Figure S24: Percentage of subjects failing for each sulcus in QTIM. The primary sulci are highlighted in red.

For HCP, 816 subjects were analyzed and no subject failed for the whole set of sulci. On average, HCP subjects failed for 4 sulci (st dev. 2) (**Figure S24**). **Figure S26** reports the number of subjects failing (in percentage of the total population) for left and right hemispheres.

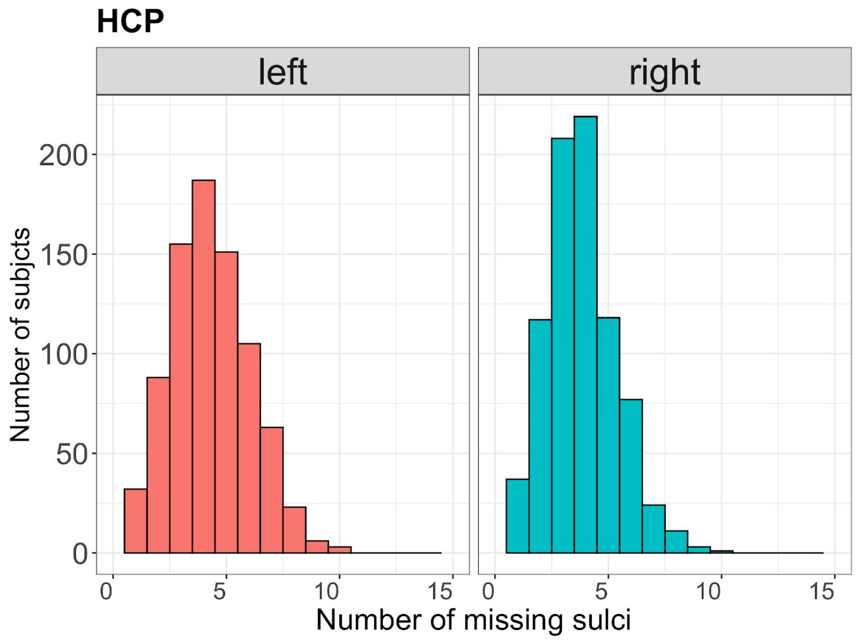

Figure S25: Histogram of number of subjects counted for missing left and right sulci, in the HCP dataset. The missing sulci are removed before outlier detection.

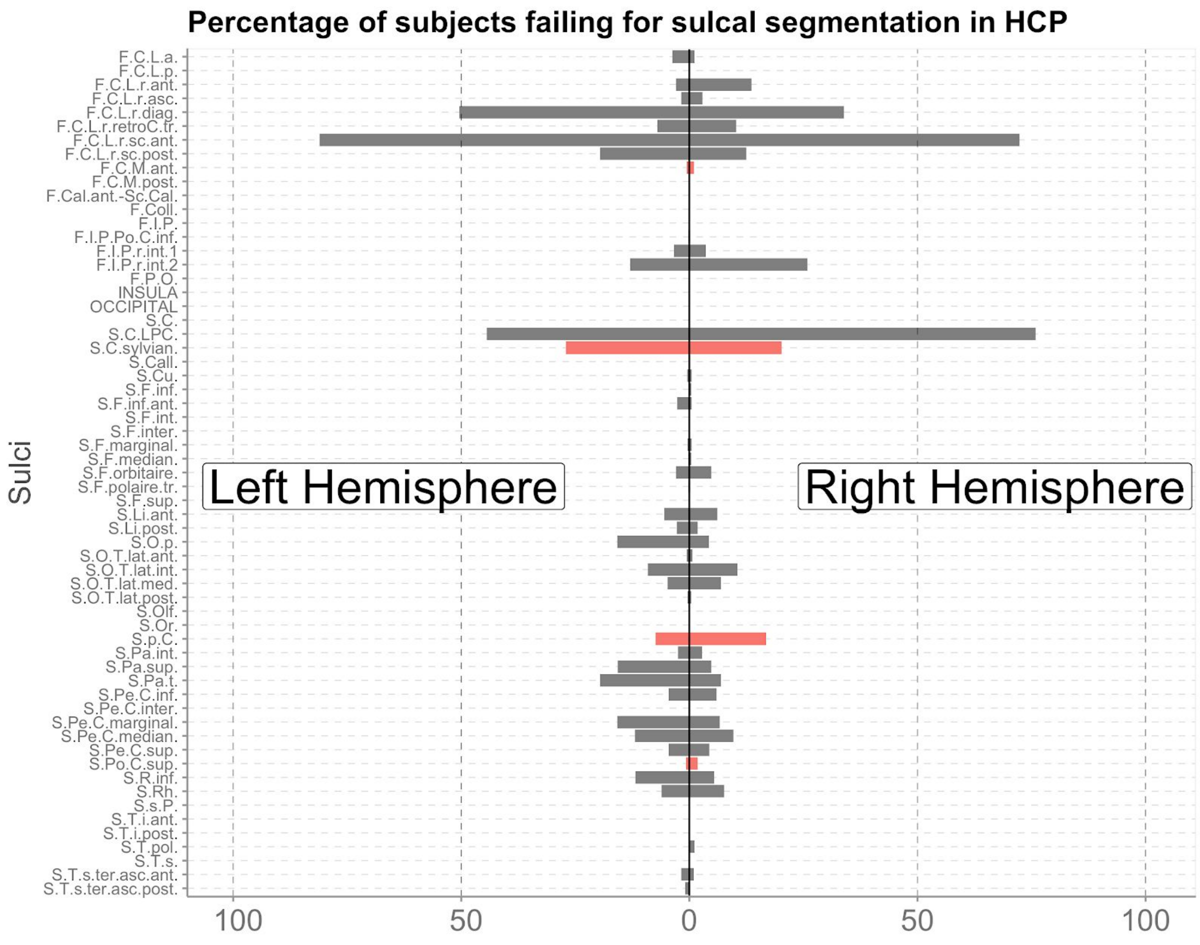

Figure S26: Percentage of subjects failing for each sulcus in HCP. The primary sulci are highlighted in red.

For GOBS, data from 1,025 subjects were analyzed and no subject failed for the whole set of sulci. On average, GOBS subjects failed for 5 (st dev. 2) sulci (**Figure S27**). **Figure S28** reports the number of subjects failing (in percentage of the total population) for left and right hemispheres.

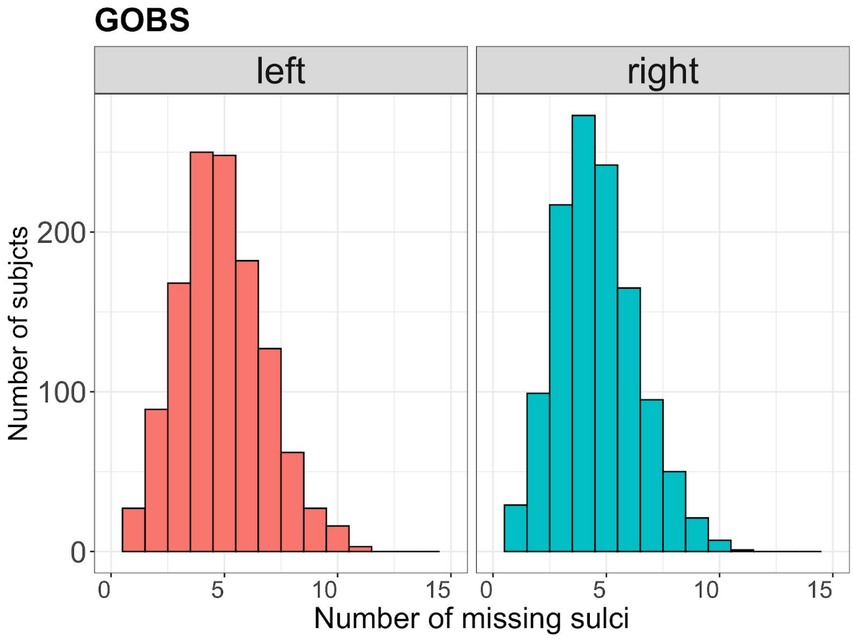

Figure S27: Histogram of number of subjects counted for missing left and right sulci, in the GOBS dataset. The missing sulci are removed before outlier detection.

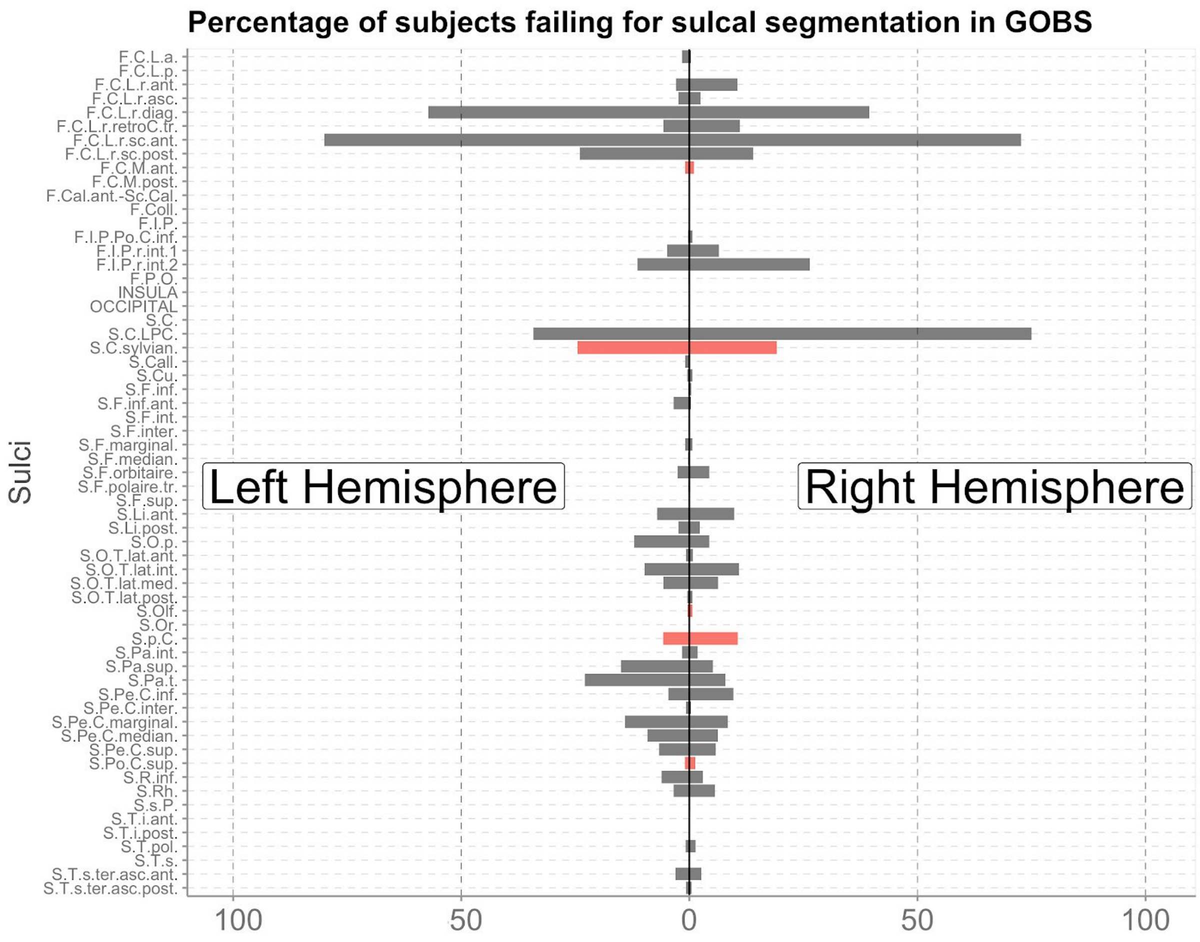

Figure S28: Percentage of subjects missing for each sulcus in the GOBS dataset. The primary sulci are highlighted in red.

For UKBB, we analyzed data from 10,083 subjects. We found that 150 subjects failed for the whole set of sulci. On average, UKBB subjects failed for 4 sulci (st dev. 2) (**Figure S29**). **Figure S30** reports the number of subjects failing (in percentage of the total population) for left and right hemispheres.

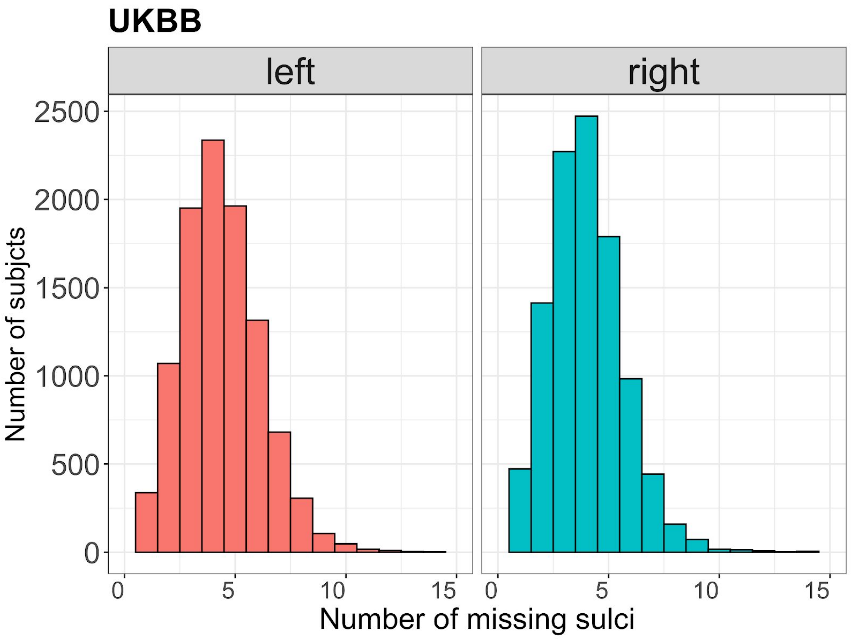

Figure S29: Histogram of number of subjects counted for the missing left and right sulci, in the UKBB dataset. The missing sulci are removed before outlier detection.

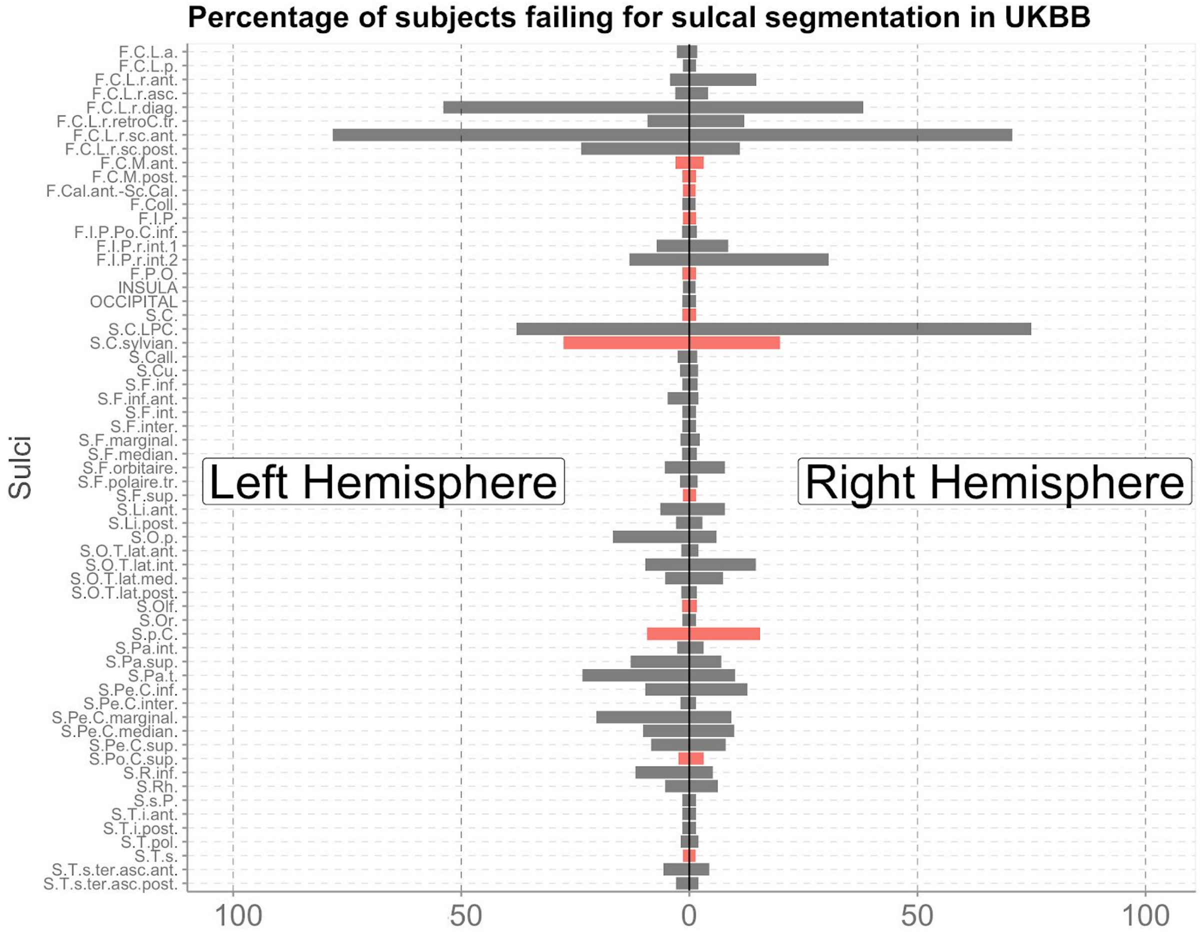

Figure S30: Percentage of subjects missing for each sulcus in the UKBB dataset. The primary sulci are highlighted in red.

6) It would be helpful to see a figure that labels the 61 different sulci in the study on brain surfaces. It would also be great to add a schematic of the four different sulcal measures defined.

For a representation of labeled sulci, please refer to the BrainVISA webpage following this link:
http://brainvisa.info/web/_static/images/bsa/brainvisa_sulci_atlas_with_table_150dpi-r90.png

We included **Figure S32** in the Supplementary Material showing a surface representation of labeled sulci extracted from a random KKI subject and a representation of the four measures (length, mean depth, surface area, width) extracted from each sulcus[13] .

A

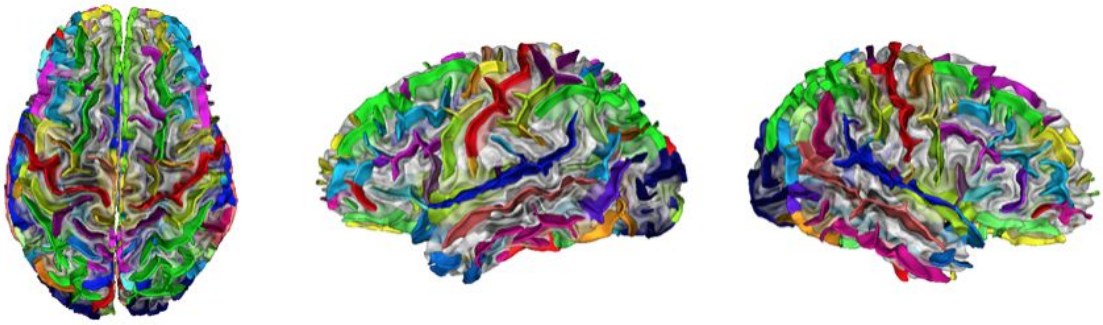

B

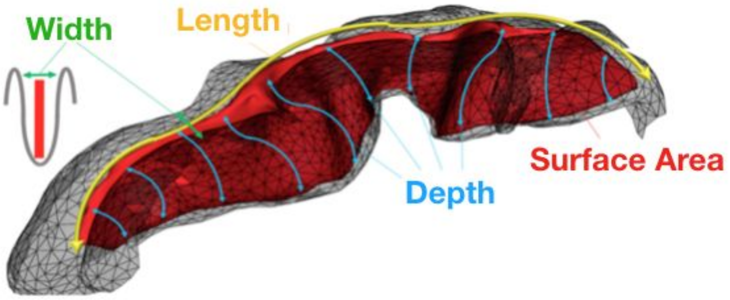

Figure RS32: A) Surface representation of labeled sulci extract from one randomly selected subject analyzed here. B) Representation of sulcal measurements (edited from [13]). For a representation of labeled sulci, please refer to the BrainVISA webpage following this link: http://brainvisa.info/web/_static/images/bsa/brainvisa_sulci_atlas_with_table_150dpi-r90.png

7) Many sulci can be hard to measure across individuals or may not even be present. Was visual quality control of these sulci done at all? For instance, this could be feasible to do for the test-retest reliability cohorts with fewer individuals. The authors discuss an automated check for outliers, but certain hard-to-measure sulci may be giving consistently biased estimates or highly heterogeneous estimates where simply dropping an outlier doesn't mean the retained subjects have anatomically accurate labels of these sulci. Which sulcus had the highest number of dropped subjects? Does it correspond to those with the lowest ICC as well?

Please see our response to question 5 regarding subjects failing for sulcal segmentation. We agree with the reviewer that dropping an outlier doesn't mean that the retained subjects have automatically accurate labels. However, due to the high anatomical inter-subject variability, and the fact that an accurate label may not necessary ensure properly defined morphometric features, visually inspecting the sulcal labels in large datasets would not necessarily ensure a robust and accurate representation of sulcal labels, see e.g. Anticevic et al., Neuroimage 2012[15] for an illustration of operator biases with less variable sulci.

The test-retest reliability analysis of labelled sulci gives a measure of the robustness of the measurements, which was evaluated on four independent test-retest cohorts. While we spot checked many subjects visually for each dataset, we did not ensure the anatomical validity of the sulcus labeling at large. We now include this as a limitation of our study.

We assessed the correlation between the number of failed subjects and the ICC for each bilaterally averaged sulcal measure; we found no correlation between ICC and the number of failed subjects (**Figure S31**). **Figure S31** shows the correlation between the number of failed subjects and ICC for sulcal width.

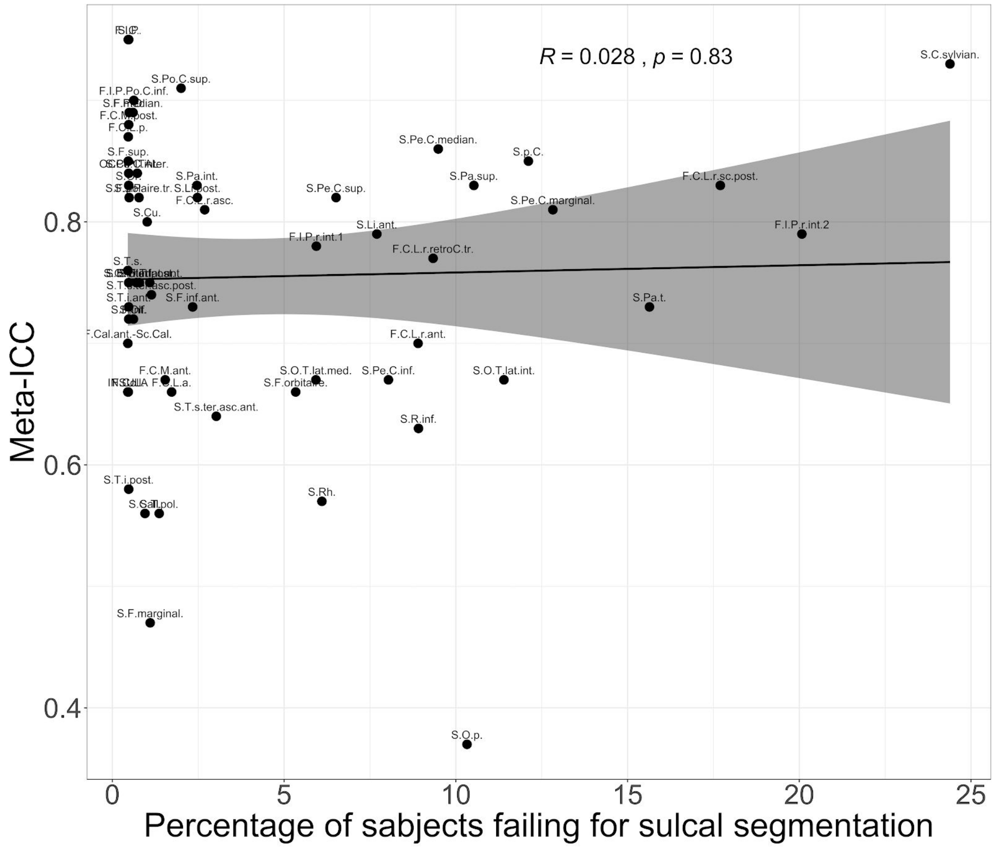

Figure S31: Correlation between Meta-ICC estimated for sulcal width and the percentage of subjects failing for sulcal segmentation averaged across cohorts (QTIM, HCP, GOBS and UKBB) and hemispheres.

Results
8) Bland-Altman plots to visualize the bias in sulcal estimates, rather than in table format, would be helpful for readers.

Thank you for this suggestion. We generated such plots
https://drive.google.com/drive/folders/1UrNsBUDXmJpx9-9EDxNqUwtSdrq5_oQK?usp=sharing

We will make this link available in the final version of the manuscript.

9) The authors note that sulcal length heritability was more often significant when NOT adjusting for ICV. Can the authors provide correlations between ICV and sulcal estimates?

Figure S33 now shows the Pearson's correlation between each of the sulcal measures analyzed here and the intracranial volume (ICV) extracted with Freesurfer and used as covariate for estimating the heritability. Length and surface area show the highest (positive) correlation among the sulci and ICV, while width showed minimal correlations..

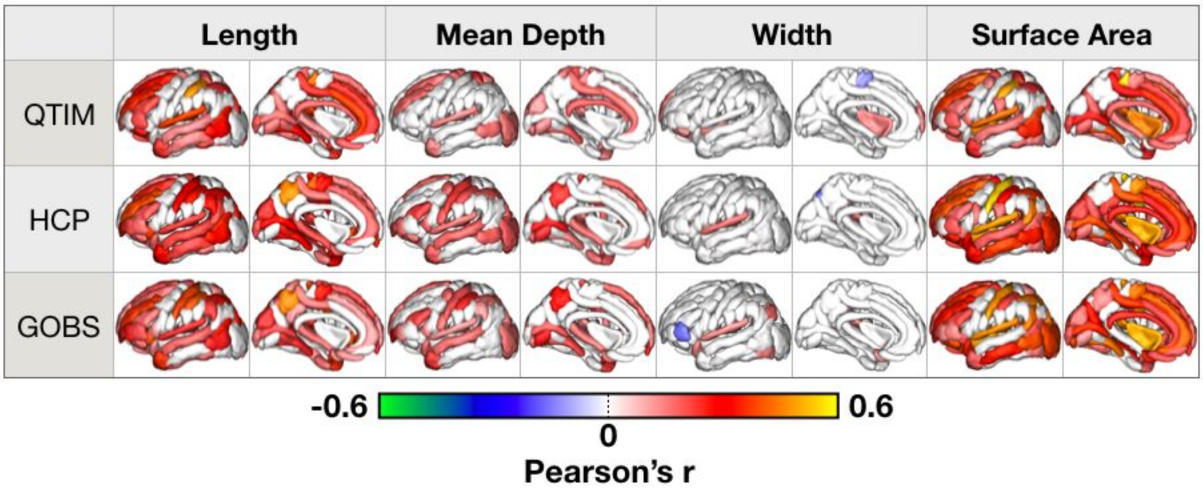

Figure S33: Pearson's correlation between sulca length, mean depth, width and surface area and ICV, computed for QTIM, HCP and GOBS (Significant regions survived Bonferroni correction for multiple comparisons across all bilateral traits and regions ($p < 0.05/(61*4)$). The average and standard deviation, estimated across sulci, of Person's correlation is tabulated in Table S28 for each sulcal descriptor and cohort.

Figure S33 is reported now in Supplementary Material and the correlation coefficients are reported in Supplementary Tables S25, S26 and S27 for QTIM, HCP and GOBS respectively. Table S28, also reported here, shows the average Pearson's correlation computed between sulcal measures and ICV..

Table S28: Average and standard deviation of the correlation between sulcal descriptors and ICV in QTIM, HCP and GOBS (*only 2 sulci survived Bonferroni correction).

**Correlation with ICV**	**QTIM**		**HCP**		**GOBS**	
	**Average**	**sd**	**Average**	**sd**	**Average**	**sd**
**Length**	0.24	0.09	0.26	0.07	0.23	0.09
**Mean Depth**	0.17	0.02	0.20	0.03	0.19	0.05
**Width**	0.07	0.14	0.1*	*	0.08	0.14
**Surface Area**	0.25	0.11	0.28	0.11	0.28	0.11

10) As the authors note, and as expected, there are significant correlations between heritability and reliability. Following this point, it may be worthwhile to covary for ICC in the heritability analyses.

We thank the reviewer for this comment, which is in line with Figure 2 where we reported the heritability for those sulci showing ICC > 0.75 only.
The heritability is performed for each sulcus with a univariate variance component analysis. As the ICC is computed for each sulcus, rather than each subject, including the ICC as covariate would add a constant in the regression model for estimating h^2 , without changing the estimated heritability. Therefore, while we do show the correlations between reliability and heritability, we focus our genetic analysis on the most reliable regions.

11) The authors include many measures of ICC, both within and between-subjects, but these reliability measures are highly dependent on the gold standard or atlas used: it is highly recommended to show and discuss the atlas upon which the sulcal estimates were derived.

The following paragraph has been included in the methods section in the main text:

MRI image processing and sulcal extraction.
Sulcal labelling has been performed using BrainVISA suite which implements the algorithm fully described in the cited paper by Perrot et al. 2011 [14]. Briefly, it relies on a probabilistic atlas of sulci. The sulcal recognition is made by combining localization and shape information. The atlas is described in details and freely accessible here: http://brainvisa.info/web/morphologist.html and can be visualized online here: http://brainvisa.info/web/webgl_demo/webgl.html.

Discussion

12) The authors describe that "earlier forming sulci" are more heritable, while still acknowledging that heritability estimates are largely impacted by reliability. To add to this interpretation, one could argue that ICC seems to be highest for sulci that are considered landmarks in the brain and are more invariant between individuals. I would suggest adding to this interpretation that it is also possible that these sulci are larger and easier to define (such as the central sulcus, lateral fissure), likely have lower ICC, and thus also higher heritability.

We thank the reviewer for this comment. In the Discussion session we note that:
"Our results show variability of sulcal-based heritability patterns across sulcal descriptors in regions with "excellent" reliability (ICC > 0.9), confirming that not all the reliable traits are necessarily heritable[16]. "

We also integrated the Discussion as following:

Here we show that most of the reliable sulcal shape descriptors are also under strong genetic control. This trend might be due to the lower variance across subjects of more robust anatomical regions, such as the central sulcus, which are easier to identify with automated image processing pipelines and less prone to segmentation errors.

13) The authors conclude that there is very little lateralization of heritability estimates across hemispheres - however, some of the samples included seemed to include only right-handed individuals. This may have impacted results - for instance, QTIM included only right-handed individuals and the authors noted that this sample had higher heritability for right hemi sulci. Thus it should be added somewhere to the discussion or limitations section that including more individuals to approximate what is seen in true populations in terms of handedness (e.g. left-handed individuals, ambidexterity) might change these results.

We thank the reviewer for this comment. We added the following considerations:

This suggests that sulcal descriptors could offer additional insights into brain lateralization, beyond more commonly collected metrics. However, our results may be biased as a large portion of our population contains only right-handed individuals; it has been shown that for brain volumes the cerebral asymmetry decreases for non-right-handed twins[17] and future investigations focusing on brain gyrification across handedness are needed to confirm these findings.

Reviewer #2 (Remarks to the Author):

This study examined the reliability and heritability of sulcal folding features including length, depth, width and surface area that were extracted from large-scale in vivo brain MRI using

BrainVisa. The study is methodologically well-designed, and the manuscript is well-written in general.

We thank the reviewer for the positive comments.

However, I have two major comments.

First, although various results were generated and reported, it's little hard to capture the difference and originality of this study when compared to prior studies. In introduction, some description is too general. It needs to be more specific, and more relevant studies have to be introduced and reviewed. Is the originality of this study using the large multi-center dataset? first analysis of the reliability and heritability of "sulcal morphometric features"? more sophisticated genetic correlation analysis? or lateralization of genetic effects on the sulcal folding? It should be clearer by comparing with more prior studies.

We agree with the reviewer and have now edited the introduction:

Effects on sulcal patterns have been reported being partially independent of those on cortical thickness or surface area [4,18].
Previous studies have investigated the genetics of cortical folds, but not across the full brain, and without ensuring the reliability of the measures themselves, or the heritability estimates. Kochunov et al. analyzed the effects of age on sulcal shape descriptors in a subset of sulci confirming gray matter loss in the human brain across adulthood [19]. The central sulcus has been the focus of many earlier publications [20–23]. Its depth has been reported to be highly heritable, with the degree of the heritability varying along its profile [23]. The heritability of the depth, length and surface area of primary sulci has been studied in baboons[24] , however the study could not confirm the hypothesis proposed by [25] as to whether the earlier forming sulci have higher heritability.
In recent work, La Guen et al studied the heritability of sulcal pits[26] in HCP and the genetic correlation of sulcal width across 10 sulci in the UK Biobank[27]. However, the reliability of the findings across populations, and the extent to which they depend on the reliability of the measures, has yet to be investigated.

Here we: a) estimate the reliability of the four sulcal shape descriptors extracted across the full brain; b) evaluate heritability of these measures across 4 independent cohorts (three family-based cohorts and one cohort of unrelated participants) to ensure the reproducibility of our results across populations; c) determine the extent to which the heritability estimates are dependent on the measurement reliability; and d) provide additional biological insights into the relationship between early forming sulci and higher heritability as well as cortical lateralization.

Another comment is about interpretation of the results. What's the interpretation for different results of the different sulcal features? What is the different biological meaning between sulcal length, depth, width and surface area in these results? In discussion, many descriptions are repetition of the results. Instead, the discussion should be more focused on the interpretation of the results and original findings of this study should be clarified and supported by better discussion.

Interpreting the interaction across morphometric (including sulcal) features is closely related to understanding the biology, biomechanics and dynamics of cortical folding during brain development.
This topic is the focus of many recent papers and discussions around deciphering the biological processes that are more likely to drive cortical folding from a biomechanical perspective.
However, as pointed in recent review papers such as Borrell, V. (2018) [28], crucial aspects of cortical folding and patterning remain unclear.
We extended the discussion as detailed below, remaining cautious not to over interpret our results.
The following paragraph has been added to the discussion:

Brain sulci may serve as prominent landmarks for identifying homologous functional regions across subjects [21,22]. BrainVISA offers the ability to automatically characterize the brain sulci at high spatial resolution, by segmenting and automatically labelling 123 sulci across the whole brain. Previous studies have reported the effects of age on sulcal shape descriptors in healthy subjects [19] and disease [29]. Here we analyzed four sulcal shape descriptors: length, mean depth, width and surface area. Sulcal length has been associated with abnormal neurodevelopmental processes [30–32] while sulcal depth and width have been correlated with neurodegenerative processes [19,33,34]. Sulcal surface area represents the combination of depth and length features.

Reviewer #3 (Remarks to the Author):

The authors have assessed the reliability of cortical sulci features extraction using the Brainvisa suite. They first demonstrated a reasonable reliability of extraction for these sulcal metrics. Then they pursued by estimating the heritability of these features. They present their results by mapping their ICC and h2 on interesting sulci brain map, showing high regional variability in both reliability and heritability estimates.
The results obtained go further here than a previous publication (1), which had only reported the heritability on part of the sulcal metrics in the UK Biobank.

We thank the reviewer for this positive comment.

However, the authors failed to compare their result with this publication.

1. eQTL of KCNK2 regionally influences the brain sulcal widening: evidence from 15,597 UK Biobank participants with neuroimaging data

Thank you for this reference. We have included this reference and others to a response to reviewer 2’s first comment, and refer this reviewer to that response.

They also did not emphasize the large difference between SNP and pedigree base heritability.
The authors only wrote “Overall, the pattern of heritability estimates were largely coherent between the family based and large-scale population studies.”

For example on Figure 2, the width of the central sulcus seems to have a Family based h2 of 0.7, while SNP based h2 is 0.2-0.3. These important differences will make it difficult for future works to settle on which level of heritability to cite for these sulcal metrics.

What could explain such large difference in heritability estimate between UK Biobank SNP-based estimate and the Family-based estimate? Methods? Age difference between cohorts? Less environmental influence in some cohorts? Measurement errors?
The authors write “SNP-based heritability estimated in the UK Biobank shows a similar pattern (Figure S20) across the brain, but with lower h2 values compared to the family based cohorts. This might be due to the “missing heritability” effect arising in the SNP-based heritability estimation 71.”
(2) and (3) below seem to contradict this hypothesis by using both methods in HCP and obtaining similar estimates for both methods. Please comment.

2. Genomic kinship construction to enhance genetic analyses in the human connectome project data
3. Heritability of surface area and cortical thickness: a comparison between the Human Connectome Project and the UK Biobank dataset

We thank the reviewer for this comment.
We agree that ideally heritability should be similar, yet we note here that a key difference between our work and those mentioned above is that we did not use an empirical kinship design for HCP as suggested by the above mentioned works, but used the approximated sibling and twin-based relationships for the kinship matrix. Furthermore, our work was conducted using variance components analysis for the twin and family models, including HCP. We agree future works comparing the empirical kinship design in family based models across all these cohorts would be of interest. When creating empirical kinship matrices, it is important to take into account ancestral differences in the HCP population, as stated in the Kochunov et al paper this will significantly influence heritability estimates, and may be confounded with some common environments. We also note that here, GCTA was conducted in UK Biobank on under 10,000 individuals, and may represent a more underpowered analysis than that presented in the Le Geun et al publication with nearly twice the sample size.

We now add the following sentence to the paragraph:

We note that recent work have found less discrepancy between twin and family heritability estimates and those derived from large-scale population studies[35], therefore, larger population samples may be required to better power our SNP-based heritability estimates and help determine the true extent of the missing heritability.

The authors also emphasize an interesting result, showing a positive correlation between the reliability of extraction and the heritability. They appropriately discuss this important confirmatory result: "Indeed, measurement error could lead to ceiling effect on heritability estimates, and genetic correlations. Highly heritable traits can be detected only if the traits are robustly measured 50 and low reliability could lead to an underestimation of the true heritability 51."

I agree with the authors that it is necessary to demonstrate both the reliability and heritability of a trait before deeming it worth to analyze in a genome-wide association study in ENIGMA like consortia. Overall, the authors demonstrated the reliability and heritability for most sulci of the metrics under consideration. Thus, this work lays the foundation to promote such large-scale meta-analysis of these sulcal metrics.

We thank the reviewer for acknowledging our considerable efforts.

Last, the authors pinpoint a strong genetic correlation between contralateral measures for a subset of sulci, while also identifying a subset with unique genetic influence in one hemisphere. The later group may reveal insights into the brain lateralization and thus promotes further genetic study of these regions analyzing left and right hemispheres separately.

We thank the reviewer for this positive comment.

Why Supplementary Tables numbering starts at S3, and includes an SA?

The enumeration of the tables has been corrected.

REFERENCES

1. Kanai, R. & Rees, G. The structural basis of inter-individual differences in human behaviour and cognition. *Nat. Rev. Neurosci.* **12**, 231–242 (2011).
2. Schmaal, L. *et al.* Cortical abnormalities in adults and adolescents with major depression based on brain scans from 20 cohorts worldwide in the ENIGMA Major Depressive Disorder Working Group. *Mol. Psychiatry* **22**, 900–909 (2017).
3. Winkler, A. M. *et al.* Cortical thickness or grey matter volume? The importance of selecting the phenotype for imaging genetics studies. *NeuroImage* vol. 53 1135–1146

(2010).

4. Panizzon, M. S. *et al.* Distinct genetic influences on cortical surface area and cortical thickness. *Cereb. Cortex* **19**, 2728–2735 (2009).

5. Tallinen, T., Chung, J. Y., Biggins, J. S. & Mahadevan, L. Gyrification from constrained cortical expansion. *Proc. Natl. Acad. Sci. U. S. A.* **111**, 12667–12672 (2014).

6. Strike, L. T. *et al.* Genetic Complexity of Cortical Structure: Differences in Genetic and Environmental Factors Influencing Cortical Surface Area and Thickness. *Cereb. Cortex* **29**, 952–962 (2019).

7. Grasby, K. L. *et al.* The genetic architecture of the human cerebral cortex. *bioRxiv* 399402 (2018) doi:10.1101/399402.

8. Meldrum, B. Core Text of Neuroanatomy. (2nd edition). By M. B. Carpenter. Baltimore: Williams and Wilkins. 1978. Pp 354. $13.95. *British Journal of Psychiatry* vol. 134 122–122 (1979).

9. BRAAK & H. Architectonics as seen by lipofuscin stains. *Cellular Components of the Cerebral Cortex* 59–104 (1984).

10. Rakic, P. Evolution of the neocortex: a perspective from developmental biology. *Nat. Rev. Neurosci.* **10**, 724 (2009).

11. Rogers, J. *et al.* On the genetic architecture of cortical folding and brain volume in primates. *Neuroimage* **53**, 1103–1108 (2010).

12. Pizzagalli, F. *et al.* The core genetic network underlying sulcal morphometry. in *12th International Symposium on Medical Information Processing and Analysis* vol. 10160 101600C (International Society for Optics and Photonics, 2017).

13. Pizzagalli, F., Auzias, G. & Kochunov, P. Genetic analysis of cortical sulci in 1,009 adults. *2016 IEEE 13th* (2016).

14. Perrot, M., Rivière, D. & Mangin, J.-F. Cortical sulci recognition and spatial normalization. *Med. Image Anal.* **15**, 529–550 (2011).

15. Anticevic, A. *et al.* Automated landmark identification for human cortical surface-based registration. *Neuroimage* **59**, 2539–2547 (2012).

16. Elliott, M. L. *et al.* General functional connectivity: Shared features of resting-state and task fMRI drive reliable and heritable individual differences in functional brain networks. *Neuroimage* **189**, 516–532 (2019).

17. Geschwind, D. H., Miller, B. L., DeCarli, C. & Carmelli, D. Heritability of lobar brain volumes in twins supports genetic models of cerebral laterality and handedness. *Proc. Natl. Acad. Sci. U. S. A.* **99**, 3176–3181 (2002).

18. Alemán-Gómez, Y. *et al.* The human cerebral cortex flattens during adolescence. *J. Neurosci.* **33**, 15004–15010 (2013).

19. Kochunov, P. *et al.* Age-related morphology trends of cortical sulci. *Hum. Brain Mapp.* **26**, 210–220 (2005).

20. Sun, Z. Y. *et al.* The effect of handedness on the shape of the central sulcus. *Neuroimage* **60**, 332–339 (2012).

21. Pizzagalli, F., Auzias, G., Delon-Martin, C. & Dojat, M. Local landmark alignment for high-resolution fMRI group studies: toward a fine cortical investigation of hand movements in human. *J. Neurosci. Methods* **218**, 83–95 (2013).

22. Coulon, O. *et al.* Two new stable anatomical landmarks on the Central Sulcus: definition, automatic detection, and their relationship with primary motor functions of the hand. *Conf. Proc. IEEE Eng. Med. Biol. Soc.* **2011**, 7795–7798 (2011).

23. McKay, D. R. *et al.* Sulcal depth-position profile is a genetically mediated neuroscientific trait: description and characterization in the central sulcus. *J. Neurosci.* **33**, 15618–15625 (2013).

24. Kochunov, P. *et al.* Genetics of primary cerebral gyrification: Heritability of length, depth and area of primary sulci in an extended pedigree of Papio baboons. *Neuroimage* **53**, 1126–1134 (2010).

25. Lohmann, G., von Cramon, D. Y. & Steinmetz, H. Sulcal variability of twins. *Cereb. Cortex* **9**, 754–763 (1999).
26. Le Guen, Y. *et al.* Genetic Influence on the Sulcal Pits: On the Origin of the First Cortical Folds. *Cereb. Cortex* **28**, 1922–1933 (2018).
27. Le Guen, Y. *et al.* eQTL of KCNK2 regionally influences the brain sulcal widening: evidence from 15,597 UK Biobank participants with neuroimaging data. *Brain Struct. Funct.* **224**, 847–857 (2019).
28. Borrell, V. How Cells Fold the Cerebral Cortex. *J. Neurosci.* **38**, 776–783 (2018).
29. Liu, T. *et al.* Longitudinal changes in sulcal morphology associated with late-life aging and MCI. *Neuroimage* **74**, 337–342 (2013).
30. Auzias, G. *et al.* Atypical sulcal anatomy in young children with autism spectrum disorder. *Neuroimage Clin* **4**, 593–603 (2014).
31. Cachia, A. *et al.* The shape of the ACC contributes to cognitive control efficiency in preschoolers. *J. Cogn. Neurosci.* **26**, 96–106 (2014).
32. Muellner, J. *et al.* Altered structure of cortical sulci in gilles de la Tourette syndrome: Further support for abnormal brain development. *Mov. Disord.* **30**, 655–661 (2015).
33. Reiner, P. *et al.* Sulcal span in Azheimer's disease, amnestic mild cognitive impairment, and healthy controls. *J. Alzheimers. Dis.* **29**, 605–613 (2012).
34. Hamelin, L., Bertoux, M., Bottlaender, M. & Corne, H. Sulcal morphology as a new imaging marker for the diagnosis of early onset Alzheimer's disease. *Neurobiology of* (2015).
35. Le Guen, Y. *et al.* Heritability of surface area and cortical thickness: a comparison between the Human Connectome Project and the UK Biobank dataset. in *2019 IEEE 16th International Symposium on Biomedical Imaging (ISBI 2019)* 1887–1890 (2019).

REVIEWERS' COMMENTS:

Reviewer #1 (Remarks to the Author):

The authors have done an excellent job addressing my comments. I have nothing else to comment.

Reviewer #3 (Remarks to the Author):

The authors appropriately addressed all my comments.

Regarding other reviewers comments, I agree with the authors that their approach appears to be the most adequate and less biased to extract the brain sulci and estimate their heritability in such a large sample.
As the authors already pointed out some sulci cannot be reliably and consistently extracted, thus they only estimated the heritability of the sulci with ICC > 0.75. However, even if reliability of extraction is a pre-requisite to estimate the heritability (otherwise the phenotypic variance includes a large proportion of measurement errors), it does not imply that every sulci reliably extracted will have high heritability as the authors emphasized.
